# Halogen‐Free π‐Conjugated Polymers Based on Thienobenzobisthiazole for Efficient Nonfullerene Organic Solar Cells: Rational Design for Achieving High Backbone Order and High Solubility

**DOI:** 10.1002/advs.202205682

**Published:** 2022-12-18

**Authors:** Naoya Nakao, Masahiko Saito, Tsubasa Mikie, Takumi Ishikawa, Jihun Jeon, Hyung Do Kim, Hideo Ohkita, Akinori Saeki, Itaru Osaka

**Affiliations:** ^1^ Applied Chemistry Program Graduate School of Advanced Science and Engineering Hiroshima University 1‐4‐1 Kagamiyama Higashi‐Hiroshima Hiroshima 739‐8527 Japan; ^2^ Department of Polymer Chemistry Graduate School of Engineering Kyoto University Kyoto 615‐8510 Japan; ^3^ Department of Applied Chemistry Graduate School of Engineering Osaka University 2‐1 Yamadaoka Suita Osaka 565‐0871 Japan

**Keywords:** halogen, nonfullerene, organic photovoltaics, thiazole, π‐conjugated polymer

## Abstract

In π‐conjugated polymers, a highly ordered backbone structure and solubility are always in a trade‐off relationship that must be overcome to realize highly efficient and solution‐processable organic photovoltaics (OPVs). Here, it is shown that a π‐conjugated polymer based on a novel thiazole‐fused ring, thieno[2′,3′:5,6]benzo[1,2‐*d*:4,3‐*d*′]bisthiazole (TBTz) achieves both high backbone order and high solubility due to the structural feature of TBTz such as the noncovalent interlocking of the thiazole moiety, the rigid and bent‐shaped structure, and the fused alkylthiophene ring. Furthermore, based on the electron‐deficient nature of these thiazole‐fused rings, the polymer exhibits deep HOMO energy levels, which lead to high open‐circuit voltages (*V*_OC_s) in OPV cells, even without halogen substituents that are commonly introduced into high‐performance polymers. As a result, when the polymer is combined with a typical nonfullerene acceptor Y6, power conversion efficiencies of reaching 16% and *V*_OC_s of more than 0.84 V are observed, both of which are among the top values reported so far for “halogen‐free” polymers. This study will serve as an important reference for designing π‐conjugated polymers to achieve highly efficient and solution‐processable OPVs.

## Graphical abstract

A halogen‐free π‐conjugated polymer based on a novel thiazole‐based fused ring, thieno[2′,3′:5,6]benzo[1,2‐*d*:4,3‐*d*′]bisthiazole (TBTz), is designed and synthesized to have high backbone order and high solubility. The polymer shows a high efficiency of approximately 16% in nonfullerene organic solar cells, which is among the highest values for halogen‐free π‐conjugated polymers.

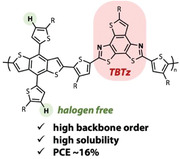

## 1 Introduction

Tremendous efforts have been devoted to the development of π‐conjugated polymers for use as p‐type and n‐type (i.e., donor and acceptor) semiconducting materials toward the goal of improving the performance of organic photovoltaics (OPVs) in the last few decades.^[^ ^1^, ^2^, ^3^, ^4^ ^]^ With the emergence of nonfullerene acceptors in place of fullerene acceptors,^[^ ^5^, ^6^, ^7^ ^]^ OPVs that use π‐conjugated polymers as the electron donor have recently been shown to demonstrate power conversion efficiencies (PCEs) in excess of 18%.^[^ ^8^, ^9^, ^10^ ^]^ In general, the device performance of OPVs relies on the structural order of the polymers, thus the high backbone coplanarity and/or order is one of the important design strategies. However, enhanced backbone coplanarity typically lowers the solubility of the polymers, which limits solution processability and fine control of the morphology/phase separation. This trade‐off must be overcome in order to achieve highly efficient and solution‐processable OPVs.

**Figure 1:**
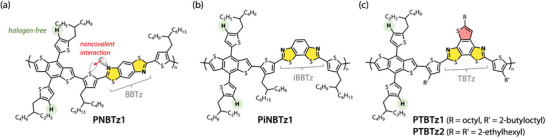
Chemical structures of the π‐conjugated polymers based on a) benzo[1,2‐*d*:4,5‐*d* *′*]bisthiazole (BBTz) PNBTz1, b) benzo[1,2‐*d*:4,3‐*d* *′*]bisthiazole (iBBTz) PiNBTz1, and c) thieno[2′,3′:5,6]benzo[1,2‐*d*:4,3‐*d* *′*]bisthiazole (TBTz) PTBTz1 and PTBTz2.

The use of fused rings as a building unit is a familiar strategy for the design of π‐conjugated polymers with high backbone order.^[^ ^11^, ^12^, ^13^ ^]^ In parallel, the use of thiazole rings can further increase the coplanarity of the backbone to achieve high backbone order.^[^ ^14^, ^15^, ^16^ ^]^ Linking a thiazole moiety with thiophene at the 2‐position is considered to induce a noncovalent interaction between the thiazole nitrogen and thiophene sulfur due to the donation of a nitrogen lone pair to the antibonding orbital of the C—S bond, which in turn interlocks the linkage and prevents torsion (**Figure** **1**a).^[^ ^17^ ^]^ For example, we previously reported that a π‐conjugated polymer, named PNBTz1 (Figure 1a), which incorporates benzo[1,2‐*d*:4,5‐*d*′]bisthiazole (BBTz) in combination with benzo[1,2‐*b*:4,5‐*b*′]dithiophene (BDT) showed reasonably high photovoltaic performance likely due to the high backbone order originating in the rigid‐rod structure.^[^ ^18^ ^]^ Moreover, it is important to note that because of the electron‐deficient nature of the BBTz unit, PNBTz1 provided deep highest occupied molecular orbital (HOMO) energy levels and thereby high open‐circuit voltages (*V*_OC_s) despite the fact that it possesses no halogen groups (halogen groups are typically introduced to the BDT unit as alkylthiophene substituents in most high‐performance polymers to ensure deep HOMO energy levels).^[^ ^19^, ^20^, ^21^ ^]^ These findings suggest that thiazole‐based fused rings have great advantages over other building units for donor polymers, as halogenation, particularly fluorination, requires additional synthetic steps under severe cryogenic conditions with relatively low reaction yields, resulting in higher synthetic costs.^[^ ^19^, ^22^ ^]^ Indeed, several groups have recently reported on the importance of halogen‐free π‐conjugated polymers.^[^ ^23^, ^24^, ^25^, ^26^ ^]^

As we continued our study, however, we found that the solubility of PNBTz1 significantly decreased with increasing molecular weight, which significantly deteriorated its solution processability. As high‐molecular‐weight polymers have great advantages over their low‐molecular‐weight counterparts in terms of film forming property and the ability to form extended polymer networks in the film, which are crucial for achieving high‐performance π‐conjugated polymers for OPVs, it is desirable that the solubility is kept high even when the molecular weight is increased. Here, we designed a series of new π‐conjugated polymers, named PiNBTz1 (Figure 1b), PTBTz1 and PTBTz2 (Figure 1c), that are based on benzo[1,2‐*d*:4,3‐*d* *′*]bisthiazole (iBBTz) and thieno[2′,3′:5,6]benzo[1,2‐*d*:4,3‐*d* *′*]bisthiazole (TBTz), respectively. It should be noted that iBBTz has only been used as a building unit for small molecules in organic semiconductors^[^ ^27^ ^]^ and, to the best of our knowledge, TBTz is a novel fused ring system. The bent shape of iBBTz and TBTz and the additionally fused alkylthiophene moiety of TBTz made these new polymers significantly more soluble than PNBTz1. Importantly, PTBTz2 with the optimized side chains achieved high backbone order at the same time. As a result, PTBTz2 exhibited improved photovoltaic performance with PCEs as high as 15.9% by using Y6 as the acceptor material. We also note that these polymers showed *V*_OC_s of more than 0.84 V. These PCEs as well as *V*_OC_s are among the top values reported so far for halogen‐free polymers.^[^ ^23^, ^24^, ^25^, ^26^ ^]^ This study demonstrates that TBTz is a promising building unit for high‐performance π‐conjugated polymers for OPVs, and that careful molecular design can achieve polymers with both high backbone order and high solubility.

## 2 Results and Discussion

### 2.1 Polymer Synthesis

**Scheme 1:**
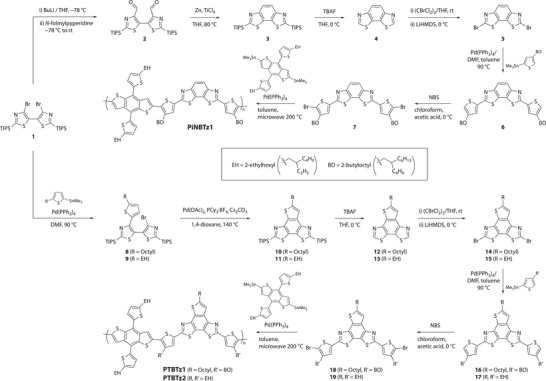
Synthesis of PiNBTz1, PTBTz1, and PTBTz2.

The synthesis of PiNBTz1 is illustrated in **Scheme** **1** (upper panel). Although synthetic methodologies for iBBTz have been reported,^[^ ^27^, ^28^, ^29^ ^]^ they require a relatively large number of synthetic steps and/or expensive reagents. We thus designed a new synthetic route to iBBTz using 4,4′‐dibromo‐2,2′‐bis(triisopropylsilyl)‐5,5′‐bithiazole (**1**),^[^ ^30^ ^]^ which was diformylated to give **2**, followed by cyclization via the McMurry reaction to afford disilylated iBBTz derivative (**3**). After desilylation (**4**) and subsequent dibromination (**5**), the iBBTz derivative was cross‐coupled with stannylated 3‐(2‐butyloctyl)thiophene via the Stille reaction to give **6**, which was further reacted with NBS to form iBBTz monomer **7**. PiNBTz1 was finally synthesized from **7** with a benzodithiophene monomer having 2‐ethylhexyl groups as thiophene substituents. Note that the alkyl groups used for PiNBTz1 were exactly the same as those used for PNBTz1.

PTBTzs were also synthesized from common intermediate **1** (Scheme 1, lower panel). The Stille reaction of **1** with stannylated 5‐alkylthiophene having either an octyl group or a 2‐ethylhexyl group as the alkyl substituent (R) provided alkylthiophene‐substituted bithiazole derivatives **8** and **9**, respectively. The cyclization of **8** and **9** via the intramolecular C—H activation reaction afforded disilylated TBTz derivatives **10** and **11**, respectively, which were then desilylated (**12** and **13**) and dibrominated (**14** and **15**). Cross‐coupling of **14** and **15** with stannylated thiophene having either a 2‐butyloctyl group or a 2‐ethylhexyl group as the substituent R′ yielded **16** and **17**, respectively, which were dibrominated to give TBTz monomers **18** and **19**, respectively. Polymerization of **18** and **19** with benzodithiophene monomers yielded PTBTz1 (R = octyl, R′ = 2‐butyloctyl) and PTBTz2 (R = R′ = 2‐ethylhexyl), respectively. It should be noted that, given the unsymmetrical structure of TBTz and the absence of regioselectivity in the TBTz monomers (**18** and **19**), neither PTBTz1 nor PTBTz2 backbone has a regioregular structure.

Number‐average and weight‐average molecular weights (*M*_n_ and *M*_w_, respectively) of PiNBTz1 were 81 300 and 153 000, respectively, with a dispersity (*Ð*) of 1.88 (Figure S1, Supporting Information). The *M*_n_ and *M*_w_ of PTBTz1 were 73 600 and 166 000 (*Ð* = 2.26), respectively, and those of PTBTz2 were 74 100 and 162 300 (*Ð* = 2.19), respectively. These molecular weights were all higher than or similar to those of PNBTz1 mainly used in this study for comparison (*M*_n_ = 56 000, *M*_w_ = 133 000, *Ð* = 2.38). Differential scanning calorimetry (DSC) measurements revealed no phase transition peak in the range of 20–350 °C for PiNBTz1 and PTBTz2, whereas PTBTz1 showed a phase transition peak that appeared to correspond to a melting point at 328 °C (Figure S2, Supporting Information). All the polymers were thus considered thermally stable.

We tested the solubility of the polymers in chloroform (CF) at 50 °C. The maximum concentration for PNBTz1 was approximately 12 g L^−1^. However, the solubility was significantly decreased to 6 g L^−1^ when the molecular weight was increased (*M*_n_ = 78 500), which was detrimental to the device fabrication. On the other hand, the maximum concentration for PiNBTz1, PTBTz1, and PTBTz2 was approximately 14, 18, and 12 g L^−1^, which were more than twice higher than PNBTz1 with the similar molecular weight.

### 2.2 Polymer Properties and Structures

**Figure 2:**
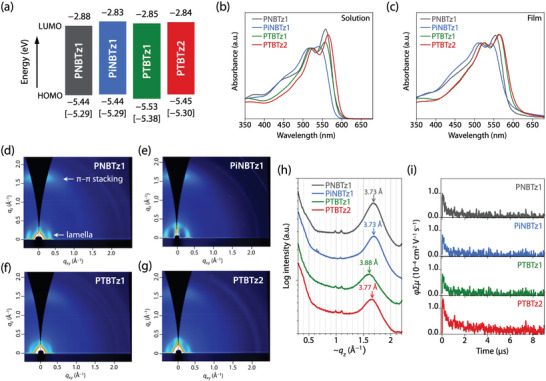
a) Energy diagrams of the polymers. HOMO and LUMO energy levels were determined by the cyclic voltammetry. The HOMO energy levels in the square brackets were the value obtained by the photoemission yield spectroscopy. b,c) UV–vis absorption spectra of the polymers in b) CF solution and c) film. d–g) 2D GIXD patterns of the polymer neat films: d) PNBTz1, e) PiNBTz1, f) PTBTz1, and g) PTBTz2. h) Cross‐sectional profiles along the quasi‐*q_z_* axis of 2D GIXD patterns. i) Laser‐flash TRMC kinetic traces of the polymer neat films (*λ*_ex_ = 355 nm).

HOMO and lowest unoccupied molecular orbital (LUMO) energy levels were estimated by cyclic voltammetry (CV) measurements of polymer thin films (**Figure** **2**a and Figure S4, Supporting Information). PiNBTz1 and PTBTz2 had HOMO energy levels in the range of −5.45 to −5.44 eV, which were similar to those of PNBTz1, which were consistent with the computation (Figure S5, Supporting Information). However, PTBTz1 had a slightly deeper HOMO energy level value of −5.53 eV, possibly due to its less ordered backbone structure as will be discussed later. The LUMO energy levels were estimated to be around −2.85 eV for all the polymers. The HOMO energy levels were also estimated using photoemission yield spectroscopy, in which the trends across the polymers were mostly the same (Figure S6, Supporting Information and Figure 2a). Importantly, the HOMO energy levels of the polymers were comparable to those of a benchmark polymer PM6 having electron‐withdrawing fluorine groups on the BDT unit (Figure S7, Supporting Information). We also note that the optimized geometry of the model for all the polymers showed that dihedral angles between the alkylthiophene and thiazole of the BBTz, iBBTz, and TBTz rings were negligible, indicating that these fused rings can indeed significantly coplanarize the linkage.

Figure 2b shows the UV–vis absorption spectra of the polymers in CF solution. All polymers showed an absorption band covering the wavelength range of 300 to 600 nm with a vibrational structure; the absorption maxima (*λ*_max_) corresponding to 0–0 and 0–1 transitions appeared at 550–560 nm and 515–525 nm, respectively. Note that the spectra were normalized at the 0–1 transition peak (see Figure S8a, Supporting Information for the spectra with the absorption coefficient). The intensity of the 0–0 band with respect to the 0–1 band (*I*_0–0_/*I*_0–1_) was lower for PiNBTz1 (1.02) and PTBTz1 (1.09), both having a bent‐shaped core, than for PNBTz1 (1.27), having a linear‐shaped core. These findings suggest lower backbone order for PiNBTz1 and PTBTz1 than for PNBTz1. On the other hand, PTBTz2 had an *I*_0–0_/*I*_0–1_ ratio of 1.19, which was much higher than that of PTBTz1, despite that the two polymers have the same backbone structure, but was close to that of PNBTz1. Thus, the backbone order of PTBTz2 was considered to be as high as that of PNBTz1. Figure 2c shows the absorption spectra of the polymer thin films, which was also normalized at the 0–1 transition peak (see Figure S8b, Supporting Information for the spectra with the absorption coefficient). In the thin film, PiNBTz1 showed a lower *I*_0–0_/*I*_0–1_ ratio (0.99) than PNBTz1 (1.10) as is the case in the solution. However, PTBTz1 showed an *I*_0–0_/*I*_0–1_ ratio of 1.11, which is almost the same as that for PNBTz1, suggesting that the backbone order was enhanced by aggregation. PTBTz2 also had the same *I*_0–0_/*I*_0–1_ ratio of 1.11. Therefore, the backbone order of PTBTzs is similar to that of PNBTz1, whereas that of PiNBTz1 is lower.

The polymer packing order was also investigated by the grazing incidence wide‐angle X‐ray diffraction (GIXD) measurements of polymer neat films (Figure 2d–g). All the polymers showed two‐dimensional (2D) GIXD patterns assignable to the face‐on orientation as the diffractions corresponding to the lamella structure and π–π stacking were observed along the *q_xy_* and quasi‐*q_z_* axes, respectively. The relatively broad and weak diffractions indicate that the polymers have low crystallinity (Figure 2h and Figure S8, Supporting Information). Although the four polymers showed similar crystallite coherence lengths, some differences were noted in π–π stacking distance ($d_{\pi }$) (Table S1, Supporting Information). The $d_{\pi }$ value was the same for PiNBTz1 and PNBTz1, at 3.73 Å. As for PTBTzs, PTBTz2 also had a similar $d_{\pi }$ of 3.77 Å, whereas PTBTz1 had a $d_{\pi }$ of 3.88 Å. We also conducted the GIXD measurements for polymer/Y6 blend films (Figure S9, Supporting Information), which similarly exhibited diffraction patterns assignable to the face‐on orientation. Notably, however, the $d_{\pi }$ value was ≈3.6 Å in all cases, which differed from the values obtained for the polymer neat films but was rather similar to that for the Y6 neat film (Figure S10 and Table S2, Supporting Information). Thus, the diffraction observed along the quasi‐*q_z_* axis in the blend films can be assigned to the molecular order of Y6, specifically, its π–π stacking. The polymer diffraction may be absent or too weak to be detected (i.e., hidden by the Y6 diffraction), suggesting that the polymers form an amorphous‐like structure.

We conducted the time‐resolved microwave conductivity (TRMC) measurements, which estimate the short‐range charge transport.^[^ ^31^ ^]^ The charge transport property is quantified by *φ*Σ*µ*, where *φ* is the quantum yield of charge generation by laser irradiation in the measurement, and Σ*µ* is the sum of hole and electron charge carrier mobilities (Figure 2i). In the polymers examined in the present study, the hole should be the dominant carrier. PiNBTz1 and PTBTz1 exhibited *φ*Σ*µ* of 0.68 × 10^−4^ cm^2^ V^−1^ s^−1^ and 0.65 × 10^−4^ cm^2^ V^−1^ s^−1^, respectively, both of which were lower than that of PNBTz1 (0.96 × 10^−4^ cm^2^ V^−1^ s^−1^). PTBTz2 exhibited *φ*Σ*µ* of 1.3 × 10^−4^ cm^2^ V^−1^ s^−1^, which was slightly higher than that of PNBTz1. We presume that the lower value for PiNBTz1 is attributed to its lower backbone order, whereas the lower value for PTBTz1 is attributed to its wider π–π stacking. The higher value for PTBTz2 compared with PNBTz1, despite that the backbone and π–π stacking orders were similar, could be due to the larger π‐system of the TBTz unit compared with the BBTz unit, which possibly led to the enhanced interchain charge transport via enhanced interchain π–π overlap.

### 2.3 Photovoltaic Performance

**Figure 3:**
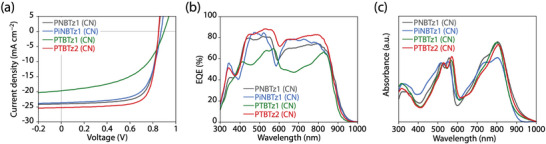
a) *J*–*V* curves and b) EQE spectra of the polymer/Y6 cells, and c) UV–vis absorption spectra of the polymer/Y6 blend films.

We fabricated OPV cells with a conventional structure (ITO/PEDOT:PSS/active layer/PDINO/Ag), in which a blend of polymer and Y6 with a weight ratio of 1:1.2 was spun from CF solution containing 0.5 vol% of 1‐chloronaphthalene (CN) as the additive (**Figure** **3**a,b and Table 1). For more details of the OPV cells with different polymer:Y6 ratios, solvent additive, posttreatments, and active layer thicknesses, see Figures S11–S20 and Tables S3–S11 (Supporting Information). PiNBTz1 showed a short‐circuit current density (*J*_SC_) of 23.6 mA cm^−2^, *V*_OC_ of 0.87 V, and a fill factor (FF) of 0.66, resulting in a PCE of 13.9%, which was lower than that for PNBTz1 (PCE = 14.7%, *J*_SC_ = 24.0 mA cm^−2^, *V*_OC_ = 0.85 V, FF = 0.72). In particular, the FF of PiNBTz1 was significantly lower than that of PNBTz1, which may be due to the lower backbone order of PiNBTz1. However, PiNBTz1 consistently showed a higher *V*_OC_ than that of PNBTz1, even though both had almost the same HOMO energy level. The lower FF and higher *V*_OC_ of PiNBTz1 compared with PNBTz1 may be explained by the difference in structural order at the polymer/Y6 interface between these polymers, which originates from the difference in backbone order.^[^ ^18^, ^32^ ^]^ Here, we show the absorption spectra of the polymer/Y6 blend films, which is normalized at the 0–1 transition peak of the polymer (see Figure S8c, Supporting Information for the spectra with the absorption coefficient). In fact, the *I*_0–0_/*I*_0–1_ ratio of PiNBTz1 (1.02) was lower than that of PNBTz1 (1.09) in the blend films (Figure 3c, Table 1). Moreover, the absorption band of Y6 in the PiNBTz1/Y6 blend film largely differed from that observed in the other polymer blend films: it showed a relatively large shoulder at around 700 nm with respect to the peak at around 800 nm. This finding suggests that the difference in Y6 packing may also account for the difference in photovoltaic performance.

**Table 1:** Photovoltaic properties of the polymer/Y6 cells

Polymer	*J*_SC_ [*J*_SC_^EQE^] [mA cm^−2^]^a)^	*V*_OC_ [V]	FF	PCE [PCE_ave_] [%]^b)^	*I*_0–0_/*I*_0–1_
PNBTz1	24.0 [24.1]	0.85	0.72	14.7 [14.3]	1.09
PiNBTz1	23.6 [23.6]	0.87	0.66	13.9 [13.3]	1.02
PTBTz1	19.6 [18.4]	0.89	0.51	9.0 [8.5]	1.05
PTBTz2	26.3 [26.4]	0.84	0.72	15.8 [15.4]	1.11
	25.2 [25.3]^c)^	0.85^c)^	0.75^c)^	15.9 [15.6]^c)^	

^a)^ *J*_SC_^EQE^: *J*_SC_ calculated from the EQE spectrum;

^b)^ PCE: maximum power conversion efficiency. PCE_ave_: average power conversion efficiency from more than 10 different cells with the optimized conditions;

^c)^ PNDIT‐F3N‐Br was used as the electron‐transporting layer instead of PDINO.

PTBTz1 showed lower performance with a PCE of 9.0% (*J*_SC_ = 19.6 mA cm^−2^, *V*_OC_ = 0.89 V, FF = 0.51) than PNBTz1. This is in agreement with the fact that the *I*_0–0_/*I*_0–1_ ratio of PTBTz1 (1.05) was lower than that of PNBTz1 (1.09) in the blend film, whereas that was similar in the neat film. Furthermore, although the polymer diffraction was absent or hidden in the 2D GIXD pattern of the blend film, PTBTz1 should have a wider $d_{\pi }$ than PNBTz1, as observed in the neat film; this is also detrimental to its photovoltaic performance. On the other hand, PTBTz2 showed significantly higher OPV performance than PNBTz1, with a PCE of 15.8% (*J*_SC_ = 26.3 mA cm^−2^, *V*_OC_ = 0.84 V, FF = 0.72). This was consistent with the highest *I*_0–0_/*I*_0–1_ ratio (1.11) among these polymers. In addition, the PTBTz2 cell exhibited a slightly improved PCE of 15.9%, where the FF was significantly increased, by changing the electron transporting layer from PDINO to PNDIT‐F3N‐Br (Figure S21, Supporting Information and Table 1). The PCEs of reaching 16% is among the highest values reported so far for the OPVs based on halogen‐free polymers (Table S13, Supporting Information).

Here, it is important to note that the observed *V*_OC_ values (0.84–0.89 V) are mostly the same as or higher than those of the cells using polymers with halogen groups as strong electron‐withdrawing substituents (e.g., PM6) (Figure S22 and Table S12, Supporting Information) and an acceptor material having a similar bandgap with Y6. Further, the *V*_OC_ values are also significantly higher than those for most of the halogen‐free polymers reported so far, which typically give *V*_OC_s of below 0.83 V (Table S13, Supporting Information). This demonstrates the great ability of the thiazole‐fused rings examined in the present study to provide deeper HOMO energy levels, and hence, higher *V*_OC_ values.

To further understand the difference in the photovoltaic performance, we studied the photoluminescence quenching efficiency of the blend films (Figure S23, Supporting Information). The quenching efficiency of the PiNBTz1 blend was approximately 90% when excited with 800 nm, which was similar to that of the PNBTz1 blend (≈90%). For PTBTzs, whereas the quenching efficiency of was ≈70%, that of the PTBTz2 blend was ≈100%. Subsequently, we evaluated the ratio of *J*_SC_ to the reverse saturation photocurrent density (*J*_ph,sat_) (Figure S24, Supporting Information). Here, *J*_ph,sat_ was estimated by using the Hecht equation.^[^ ^33^ ^]^ Typically, it can be assumed to be the charge collection efficiency,^[^ ^34^, ^35^, ^36^, ^37^ ^]^ which is, however, often discussed as a charge dissociation probability.^[^ ^38^, ^39^ ^]^ The charge collection efficiency was 95% for the PiNBTz1 cell, which was slightly lower than the PNBTz1 cell (97%). On the other hand, the PTBTz2 cell showed a slightly higher value of 96% while the PTBTz1 cell showed a significantly lower value of 88%. The results are well‐correlated with the photovoltaic performance. We also studied the bimolecular recombination by plotting the *J*_SC_ values as a function of light intensity (Figure S25, Supporting Information). However, the difference was very small and therefore the recombination may not be the critical factor for the difference in the photovoltaic performances.

The differences in photovoltaic performance can be adequately explained by considering the short‐range charge transport evaluated by the TRMC method as described above as well as the long‐range bulk charge transport that can be evaluated on the basis of the space charge limited current (SCLC) model. The hole mobility according to the SCLC model, which was estimated by measurements on the hole‐only device using polymer/Y6 blend films (Figure S26, Supporting Information), was 0.63 × 10^−4^ cm^2^ V^−1^ s^−1^ for the PiNBTz1 blend film and 0.96 × 10^−4^ cm^2^ V^−1^ s^−1^ for the PTBTz1 blend film. These values were lower than that of the PNBTz1 blend film (4.5 × 10^−4^ cm^2^ V^−1^ s^−1^). On the other hand, the PTBTz2 blend film exhibited a similar hole mobility (4.2 × 10^−4^ cm^2^ V^−1^ s^−1^) to that of the PNBTz1 blend film. The electron mobilities of these blend films were almost the same: 1.7 × 10^−4^ cm^2^ V^−1^ s^−1^ for the PNBTz1/Y6 film, 1.9 × 10^−4^ cm^2^ V^−1^ s^−1^ for the PiNBTz1/Y6 film, 1.7 × 10^−4^ cm^2^ V^−1^ s^−1^ for the PTBTz1/Y6 film, and 1.8 × 10^−4^ cm^2^ V^−1^ s^−1^ for the PTBTz2/Y6 film. In addition, the morphology of the blend films observed in the atomic force microscopy (Figure S27, Supporting Information) and the transmission electron microscopy (Figure S28, Supporting Information) did not provide significant differences in these polymer systems. This might be consistent with the fact that there was no significant difference in the surface energy of each material (Figure S29, Supporting Information).

### 2.4 Discussion on the Differences in Structural Order

**Figure 4:**
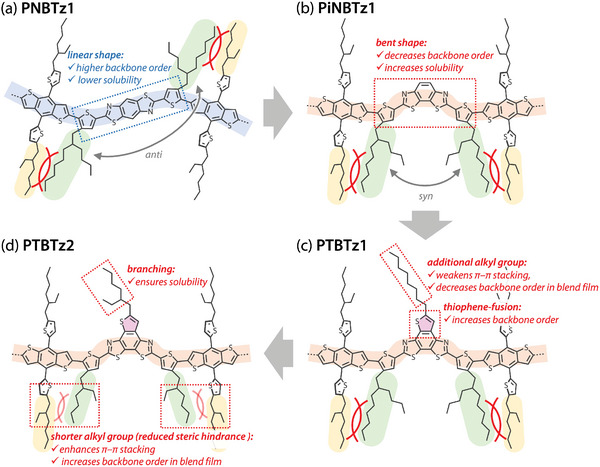
Schematic illustration of a) PNBTz1, b) PiNBTz1, c) PTBTz1, and d) PTBTz2 showing how structural differences determine backbone order.

As described above, the differences in polymer backbone order as well as π–π stacking orders are key factors that affect photovoltaic performance of the polymers. Here we discuss the correlation between the molecular structure and these structural orders. Although some steric hindrance between the side chains is present, PNBTz1 should have relatively high backbone order and/or coplanarity as discussed in the previous report,^[^ ^18^ ^]^ given the rigid, planar, and linear shape of the BDT and BBTz units (**Figure** **4**a). As for PiNBTz1, the backbone order is likely to be reduced as compared with PNBTz1 because the *I*_0–0_/*I*_0–1_ ratio is lower. This can be attributed to the bent‐shaped structure as well as the *C*_2v_ symmetry of the iBBTz unit.^[^ ^40^, ^41^, ^42^, ^43^ ^]^ Although iBBTz is also a rigid and planar fused ring, such structural differences can change the stereoregularity of the flanking alkylthiophenes (Figure 4b), i.e., the conformation of the two alkyl groups in PiNBTz1 in the most stable form becomes *syn*, whereas that in PNBTz1 is *anti*. This can lower the overall rigidity of the polymer, thereby causing torsion in the backbone. These structural features are correlated with the increased solubility of PiNBTz1 as compared with PNBTz1.

Regarding PTBTz1, despite the fact that its basic molecular structure is similar to that of PiNBTz1, the *I*_0–0_/*I*_0–1_ ratio is higher for PTBTz1 than for PiNBTz1, and is similar between PTBTz1 and PNBTz1. These findings suggest that the backbone order of PTBTz1 is relatively high, as in the case of PNBTz1. This may be because the thiophene ring fused to the iBBTz unit (i.e., TBTz unit) enhances the overall backbone rigidity (Figure 4c). However, the additional alkyl (octyl) group in the TBTz unit hinders the intermolecular interaction, as evidenced by the wider π–π stacking. Notably, however, the backbone order is somewhat reduced as a result of blending with the acceptor material. Regarding PTBTz2, the *I*_0–0_/*I*_0–1_ ratio of is similar to that of PTBTz1 in the neat film but is larger in the blend film. We presume that because the size of the alkyl groups on the thiophene rings neighboring the TBTz unit is smaller (i.e., 2‐butyloctyl for PTBTz1 and 2‐ethylhexyl for PTBTz2), the steric hindrance between the substituents is significantly reduced, thereby allowing the backbone to preserve high order even in the blend film. In addition, it is likely that the reduced steric hindrance also enhances π–π stacking in the blend film. The branched 2‐ethylhexyl group attached to the TBTz unit in PTBTz2, instead of the linear octyl group in PTBTz1, may compensate for the reduced size of the alkyl groups on the thiophene rings in the backbone and ensure the solubility of the polymer (Figure 4d).

## 3 Conclusion

In this study, we rationally designed and synthesized new π‐conjugated polymers incorporating π‐extended thiazole‐based fused rings. These polymers showed deep HOMO energy levels even without halogen groups that are often introduced as substituents in high‐performance polymers, which resulted in high *V*_OC_s of more than 0.84 V in the OPV cell combined with Y6 as the acceptor. Further, a TBTz‐based polymer PTBTz2 had high backbone order and high solubility, which led to, PCEs of as high as 15.9%. These *V*_OC_ and PCE values are among the top values observed for “halogen‐free” π‐conjugated polymers so far. Our results clearly demonstrate that a careful molecular design can provide high‐performance polymers having both high backbone order and high solubility, which are always in a trade‐off relationship. We believe that this study will serve as an important reference for designing π‐conjugated polymers to achieve highly efficient OPVs.

## Conflict of Interest

The authors declare no conflict of interest.

## Supporting information

Supporting Information

## Data Availability

The data that support the findings of this study are available in the supplementary material of this article.
