# Supplementary material for: Halogen‐Free π‐Conjugated Polymers Based on Thienobenzobisthiazole for Efficient Nonfullerene Organic Solar Cells: Rational Design for Achieving High Backbone Order and High Solubility
Source: Adv Sci (Weinh). 2022 Dec 18;10(5):2205682. doi: 10.1002/advs.202205682 (PMC9929271; doi:10.1002/advs.202205682)
Supplement: Supplementary file 1 — Supporting Information [file ADVS-10-2205682-s001.pdf]

ADVANCED SCIENCE
Open Access

Supporting Information

for *Adv. Sci.*, DOI 10.1002/advs.202205682

Halogen-Free π-Conjugated Polymers Based on Thienobenzobisthiazole for Efficient Nonfullerene Organic Solar Cells: Rational Design for Achieving High Backbone Order and High Solubility

*Naoya Nakao, Masahiko Saito, Tsubasa Mikie, Takumi Ishikawa, Jihun Jeon, Hyung Do Kim, Hideo Ohkita, Akinori Saeki and Itaru Osaka**

Supporting Information

Halogen-Free π-Conjugated Polymers Based on Thienobenzobisthiazole for Efficient Nonfullerene Solar Cells: Rational Design for Achieving High Backbone Order and High Solubility

*Naoya Nakao, Masahiko Saito, Tsubasa Mikie, Takumi Ishikawa, Jihun Jeon, Hyung Do Kim, Hideo Ohkita, Akinori Saeki, Itaru Osaka**

Materials

All chemicals were used as purchased. 4,4′-Dibromo-2,2′-bis(triisopropylsilyl)-5,5′-bithiazole (**1**),[S1] 3-(2-butyloctyl)-5-trimethylstannylthiophene,[S2] 3-(2-ethylhexyl)-5-trimethylstannyl-thiophene,[S2] 2-octyl-5-trimethylstannylthiophene,[S3] 2-(2-ethylhexyl)-5-trimethylstannyl-thiophene[S3] were synthesized according to the reported procedures. 2,6-Bis(trimethytin)-4,8-bis(5-(2-ethylhexyl)thiophen-2-yl)benzo[1,2-*b*:4,5-*b*′]dithiophene was purchased from Ossila Ltd. Polymerization was carried out using a microwave reactor, Biotage Initiator. Molecular weights of the polymers were evaluated by a high-temperature GPC (140 °C), TOSOH HLC-8121GPC/HT, using *o*-dichlorobenzene (DCB) as the eluent and calibrated with polystyrene standard. NMR spectra were measured in deuterated chloroform with a Varian System 400. Y6, PDINO, and PNDIT-F3N-Br were purchased from eFlexPV, Ltd., 1-Material, Luminescence Technology Corp., respectively.

For the solubility test of the polymers, 2 mg of a polymer sample was prepared in a vial. 0.1 mL of chloroform (CF) was first added and stirred at 50 °C, and then CF was further added until the polymer sample was completely dissolved.

Synthesis

2,2′-Bis(triisopropylsilyl)-[5,5′-bithiazole]-4,4′-dicarbaldehyde (2)

To a solution of **1** (3.80 g, 6.00 mmol) in THF (72 mL), BuLi (1.6 M THF solution, 11.2 mL, 18.0 mmol) was added dropwise at −78 °C, and the reaction solution was stirred for 2 h. 1-Formylpiperidine (2.13 mL, 19.2 mmol) was then added at −78 °C, and the solution was further stirred for 3 h, during which time the solution was warmed to room temperature. The reaction was quenched with water, and the resulting mixture was extracted with hexane. The organic layer was washed with water three times and was dried over anhydrous sodium sulfate. After the solvent was evaporated under a reduced pressure, the crude product was purified by column chromatography on silica gel with dichloromethane to give **2** as yellow oil (2.70 g, 84%). HRMS Calcd for $C_{26}H_{44}N_2S_2Si_2$ $[M+H]^+$: 537.23827. Found: 537.24585. ^{1}H-NMR (400 MHz, $CDCl_3$, TMS) δ: 10.5 (s, 2H), 1.49 (m, 6H), 1.19 (d, 36H) ^{13}C-NMR (400 MHz, CDCl3, TMS) δ: 185.10, 173.63, 152.50, 135.15, 31.58, 18.44, 11.60.

2,7-Bis(triisopropylsilyl)benzo[1,2-*d*:4,3-*d*′]bis(thiazole) (3)

To a mixture of zinc (3.90 g, 60.3 mmol) and THF (200 mL), titanium(IV) chloride (3.30 mL, 30.0 mmol) was added dropwise at 0 °C. The reaction mixture was then stirred at 80 °C for 3 h. **2** (2.70 g, 5.00 mmol) in THF (100 mL) was added dropwise, and the reaction mixture was stirred at 80 °C for 24 h. After cooling to room temperature, aqueous sodium sulfate was added and the resulting mixture was extracted with hexane. The organic layer was washed with water three times and was dried over anhydrous sodium sulfate. After the solvent was evaporated under a reduced pressure, the crude product was purified by column chromatography on silica gel with dichloromethane to give **3** as yellow solid (1.77 g, 70%). HRMS Calcd for $C_{26}H_{44}N_2S_2Si_2$ $[M+H]^+$: 505.24844. Found: 505.25586. ^{1}H-NMR (400 MHz, $CDCl_3$, TMS) δ: 8.25 (s, 2H), 1.55 (m, 6H), 1.20 (d, 36H) ^{13}C-NMR (400 MHz, $CDCl_3$, TMS) δ: 172.13, 154.09, 128.98, 121.20, 18.50, 11.76.

Benzo[1,2-*d*:4,3-*d*′]bisthiazole (4)

To a solution of **3** (1.77 g, 3.50 mmol) in THF (30 mL), tetrabutylammonium fluoride (10.5 mL, 10.5 mmol) was added dropwise at 0 °C. The reaction solution was then stirred at 0 °C for 1 h. The reaction was quenched with water, and then extracted with ethyl acetate. The organic layer was washed with water and dried over anhydrous sodium sulfate. After the solvent was evaporated under a reduced pressure, the residue was purified by column chromatography on silica gel from chloroform (CF):ethyl acetate (7:1) to give **4** as white solid (505 mg, 75%).

HRMS Calcd for $C_8H_4N_2S_2$ $[M+H]^+$:192.98159. Found:192.98859.^{1}H-NMR (400 MHz, $CDCl_3$, TMS) δ: 9.06 (s, 2H), 8.26 (s, 2H) ^{13}C-NMR (400 MHz, $CDCl_3$, TMS) δ: 153.04, 151.85, 126.94, 122.00.

2,7-Dibromobenzo[1,2-*d*:4,3-*d'*]bisthiazole (5)

To a solution of **4** (100 mg, 0.52 mmol) and 1,2-dibromo-1,1,2,2-tetracholoroethane (390 mg, 1.20 mmol) in THF (12 mL), lithium hexamethyldisilazide (LiHMDS) (1.0 M THF solution, 1.04 mL, 1.04 mmol) was added dropwise at 0 °C. The reaction mixture was then stirred for 30 min at 0 °C. The reaction was quenched with water, and the resulting precipitate was collected by filtration and washed with ethanol to give **5** as white solid (168 mg, 92%). HRMS Calcd for $C_8H_2Br_2N_2S_2$ $[M+H]^+$:348.80261. Found:348.81021.^{1}H-NMR (400 MHz, $CDCl_3$, TMS) δ: 8.07 (s, 2H) ^{13}C-NMR (400 MHz, $CDCl_3$, TMS) δ: 150.68, 137.80, 129.08, 121.50.

2,7-Bis(4-(2-butyloctyl)thiophen-2-yl)benzo[1,2-*d*:4,3-*d'*]bisthiazole (6)

A solution of **5** (350 mg, 1.00 mmol), 3-(2-butyloctyl)-5-trimethylstannylthiophene (955 mg, 2.30 mmol) and tetrakis(triphenylphosphine)palladium(0) (50.8 mg, 0.044 mmol) in 13 mL of a mixed solvent of toluene and DMF (1:1) was stirred at 90 °C for 5 h. After cooling to room temperature, the reaction was quenched with water, and the resulting mixture was extracted with hexane. The organic layer was washed with water three times and dried over anhydrous sodium sulfate. After the solvent was evaporated under a reduced pressure, the crude product was purified by column chromatography on silica gel with hexane:ethyl acetate (10:1) to give **6** as yellow oil (600 mg, 87%). HRMS Calcd for $C_{40}H_{56}N_2S_4$ $[M+H]^+$:693.33263. Found:693.34033. ^{1}H-NMR (400 MHz, $CDCl_3$, TMS) δ: 8.06 (s, 2H), 7.48 (d, 2H), 7.09 (d, 2H), 2.59 (d, 4H), 1.65 (m, 2H), 1.30 (m, 32H), 0.88 (m, 12H) ^{1}C-NMR (400 MHz, $CDCl_3$, TMS) δ: 160.80, 151.91, 143.38, 136.24, 130.35, 126.92, 125.36, 121.41, 38.39, 34.89, 33.27, 32.95, 31.89, 29.67, 28.84, 26.58, 23.03, 22.69, 14.15, 14.13.

2,7-Bis(5-bromo-4-(2-butyloctyl)thiophen-2-yl)benzo[1,2-*d*:4,3-*d'*]bisthiazole (7)

To a solution of **6** (580 mg, 0.84 mmol) in chloroform (17 mL) and acetic acid (11 mL), *N*-bromo succinimide (NBS) (330 mg, 1.84 mmol) was added at 0 °C. The reaction solution was then stirred at 0 °C for 5 h, and water was added. The resulting mixture was extracted with chloroform. The organic layer was washed with water three times, and was dried over anhydrous sodium sulfate. After the solvent was evaporated under a reduced pressure, the crude product was purified by column chromatography on silica gel with hexane:chloroform (1:2)

and recrystallized from hexane:ethanol (5:1) to give **7** as yellow solid (600 mg, 84%). HRMS Calcd for $C_{40}H_{54}Br_2N_2S_4$ $[M+H]^+$:849.15366. Found:849.16156. ^{1}H-NMR (400 MHz, $CDCl_3$, TMS) δ: 8.04 (s, 2H), 7.30 (s, 2H), 2.54 (d, 4H), 1.70 (m, 2H), 1.30 (m, 32H), 0.88 (m, 12H) ^{13}C-NMR (400 MHz, $CDCl_3$, TMS) δ: 159.71, 151.88, 142.76, 135.94, 129.56, 126.79, 121.56, 115.02, 38.53, 34.24, 33.30, 33.00, 31.89, 29.67, 28.75, 26.50, 23.04, 22.69, 14.14.

4-Bromo-4′-(5-octylthiophen-2-yl)-2,2′-bis(triisopropylsilyl)-5,5′-bithiazole (8)

To a solution of **1** (780 mg, 1.23 mmol) in DMF (26 mL), 2-octyl-5-trimethylstannylthiophene (572 g, 1.59 mmol) and tetrakis(triphenylphosphine)palladium(0) (32 mg, 0.027 mmol) were added. The reaction mixture was stirred at 90 °C for 12 h. After cooling to room temperature, the reaction was quenched with water, and the resulting mixture was extracted with hexane. The organic layer was washed with water three times and was dried over anhydrous sodium sulfate. After the solvent was evaporated under a reduced pressure, the crude product was purified by column chromatography on silica gel with hexane:dichloromethane (2:1) to give **8** as yellow oil (620 mg, 67%). HRMS Calcd for $C_{36}H_{61}BrN_2S_3Si_2$ $[M+H]^+$: 753.27188. Found: 753.27936. ^{1}H-NMR (400 MHz, $CDCl_3$, TMS) δ: 7.00 (d, 1H), 6.62 (d, 1H), 2.72 (t, 2H), 1.60 (m, 2H), 1.47 (m, 6H), 1.25–1.30 (m, 10H), 1.20 (d, 18H), 1.17 (d, 18H), 0.87 (t, 3H) ^{13}C-NMR (400 MHz, $CDCl_3$, TMS) δ:174.11, 171.80, 151.43, 147.50, 134.77, 131.44, 126.28, 126.02, 124.28, 118.51, 31.82, 31.56, 30.11, 29.27, 29.17, 29.10, 22.60, 18.53, 18.44, 14.01, 11.68, 11.57, 11.54.

4-(5-(2-(2λ^3-Ethyl)hexyl)thiophen-2-yl)-4′-bromo-2,2′-bis(triisopropylsilyl)-5,5′-bithiazole (9)

To a solution of **1** (2.50 g, 3.93 mmol) in DMF (80 mL), 2-(2-ethylhexyl)-5-trimethylstannylthiophene (1.45 g, 5.89 mmol) and tetrakis(triphenylphosphine)pallaium(0) (100 mg, 0.086 mmol) were added. The reaction mixture was then stirred at 90 °C for 12 h. After cooling to room temperature, the reaction was quenched with water, and the resulting mixture was extracted with hexane. The organic layer was washed with water three times and was dried over anhydrous sodium sulfate. After the solvent was evaporated under a reduced pressure, the crude product was purified by column chromatography on silica gel with hexane:dichloromethane (3:1) to give **9** as yellow oil (2.10 g, 71%). HRMS Calcd for $C_{36}H_{61}BrN_2S_3Si_2$ $[M+H]^+$: 753.27188. Found: 448.13245. ^{1}H-NMR (400 MHz, $CDCl_3$, TMS) δ: 7.05 (d, 1H), 6.60 (d, 1H), 2.65 (d, 2H), 1.45 (m, 7H), 1.26 (m, 8H), 1.20 (d, 18H), 1.17 (d, 18H), 0.85 (m, 6H) ^{13}C-NMR (400 MHz, $CDCl_3$, TMS) δ: 174.07, 171.80, 151.45 146.18,

134.92, 131.43, 126.38, 125.99, 125.28, 118.52, 41.41, 34.19, 32.36, 28.80, 25.53, 22.95, 18.52, 18.43, 14.02, 11.68, 11.53, 10.71.

8-Octyl-2,5-bis(triisopropylsilyl)thieno[2′,3′:5,6]benzo[1,2-*d*:4,3-*d*′]bisthiazole (10)

To a solution of **8** (1.90 g, 2.51 mmol) in 1,4-dioxane (30 mL), palladium(II) acetate (14 mg, 0.063 mmol), tricyclohexylphosphine tetrafluoroborate (46 mg, 0.13 mmol), and cesium carbonate (1.22 g, 3.76 mmol) were added. The reaction mixture was then stirred at 140 °C for 4 h. After cooling to room temperature, the reaction mixture was quenched with water, and then extracted with dichloromethane. The organic layer was washed with water and dried over anhydrous sodium sulfate. After the solvent was evaporated under a reduced pressure, the residue was purified by column chromatography on silica gel from hexane:dichloromethane (3:1) to give **10** as yellow oil (1.47 g, 87%). HRMS Calcd for $C_{36}H_{60}N_2S_3Si_2$ $[M+H]^+$: 673.34572. Found: 673.35101. ^{1}H-NMR (400 MHz, $CDCl_3$, TMS) δ: 7.82 (s, 1H), 3.05 (t, 2H), 1.86 (m, 2H), 1.55 (m, 6H), 1.25–1.35 (m, 10H), 1.23 (d, 18H), 1.21 (d, 18H), 0.87 (t, 3H) ^{13}C-NMR (400 MHz, $CDCl_3$, TMS) δ: 171.94, 170.78, 149.12, 148.40, 147.13, 133.68, 131.42, 125.77, 124.48, 120.02, 31.84, 31.66, 31.55, 30.90, 29.34, 29.22, 29.18, 22.61, 18.58, 14.02, 11.85, 11.81.

8-(2-Ethylhexyl)-2,5-bis(triisopropylsilyl)thieno[2′,3′:5,6]benzo[1,2-*d*:4,3-*d*′]bisthiazole (11)

To a solution of **9** (2.10 g, 2.65 mmol) in 1,4-dioxane (30 mL), palladium(II) acetate (30 mg, 0.13 mmol), tricyclohexylphosphine tetrafluoroborate (98 mg, 0.27 mmol), and cesium carbonate (1.70 g, 5.30 mmol) were added. The reaction mixture was then stirred at 140 °C for 4 h. After cooling to room temperature, the reaction mixture was quenched by water, and then extracted with dichloromethane. The organic layer was washed with water and dried over anhydrous sodium sulfate. After the solvent was evaporated under a reduced pressure, the residue was purified by column chromatography on silica gel from hexane:dichloromethane (3:1) to give **11** as yellow oil (1.70 g, 95%). HRMS Calcd for $C_{36}H_{60}N_2S_3Si_2$ $[M+H]^+$: 673.34572. Found: 673.35339. ^{1}H-NMR (400 MHz, $CDCl_3$, TMS) δ: 7.81 (s, 1H), 2.99 (d, 2H), 1.81 (m, 1H), 1.50–1.60 (m, 8H), 1.24 (d, 18H), 1.22 (d, 18H), 0.80–1.00 (m, 6H) ^{13}C-NMR (400 MHz, $CDCl_3$, TMS) δ:171.93, 170.78, 149.06, 148.36, 145.98, 133.63, 131.59, 125.75, 124.47, 121.00, 41.59, 35.08, 32.55, 28.88, 25.71, 22.97, 18.56, 18.54, 14.03, 11.84, 11.80, 10.81.

8-Octylthieno[2′,3′:5,6]benzo[1,2-*d*:4,3-*d*′]bisthiazole (12)

To a solution of **10** (420 mg, 0.62 mmol) in THF (6.0 mL), tetrabutylammonium fluoride (1.56 mL, 1.56 mmol) was added dropwise at 0 °C. The reaction mixture was then stirred at 0 °C for 1 h. The reaction was quenched with water, and then extracted with dichloromethane. The organic layer was washed with water and dried over anhydrous sodium sulfate. After the solvent was evaporated under a reduced pressure, the residue was purified by column chromatography on silica gel from dichloromethane to give **12** as yellow solid (260 mg, 98%). HRMS Calcd for $C_{18}H_{20}N_2S_3$ $[M+H]^+$: 361.07886. Found: 361.08563. ^{1}H-NMR (400 MHz, $CDCl_3$, TMS) δ: 9.09 (s, 1H), 9.06 (s, 1H), 7.79 (s, 1H), 3.06 (t, 2H), 1.86 (m, 2H), 1.20–1.50 (m, 10H), 0.88 (t, 3H) ^{13}C-NMR (400 MHz, $CDCl_3$, TMS) δ: 152.88, 152.85, 151.97, 151.94, 148.18, 148.18, 146.81, 146.35, 134.00, 131.90, 123.27, 121.80, 119.34, 31.81, 31.28, 30.73, 29.31, 29.16, 29.09, 22.60, 17.68, 14.02, 12.33.

8-(2-Ethylhexyl)thieno[2′,3′:5,6]benzo[1,2-*d*:4,3-*d*′]bisthiazole (13)

11 (1.20 g, 1.78 mmol) was dissolved in THF (15 mL) and cooled to 0 °C. Tetrabutylammonium fluoride (7.12 mL, 7.12 mmol) was added dropwise, and the reaction mixture was stirred for 1 h. The reaction was quenched with water, and then extracted with ethyl acetate. The organic layer was washed with water and dried over anhydrous Na_2SO_4. After the solvent was evaporated under a reduced pressure, the residue was purified by column chromatography on silica gel from hexane:ethyl acetate (3:1) to give **13** as yellow solid (490 mg, 76%). HRMS Calcd for $C_{18}H_{20}N_2S_3$ $[M+H]^+$: 361.07886. Found 361.08615. ^{1}H-NMR (400 MHz, $CDCl_3$, TMS) δ: 9.05 (s, 1H), 9.03 (s, 1H), 7.75 (s, 1H), 2.99 (d, 2H), 1.78 (m, 1H), 1.39 (m, 8H), 0.91 (m, 6H) ^{13}C-NMR (400 MHz, $CDCl_3$, TMS) δ: 152.94, 152.91, 152.02, 151.99, 147.04, 134.02, 132.16, 123.34, 121.87, 120.40, 41.50, 34.84, 32.58, 28.94, 25.69, 22.94, 14.05, 10.85.

2,5-Dibromo-8-octylthieno[2′,3′:5,6]benzo[1,2-*d*:4,3-*d*′]bisthiazole (14)

To a solution of **12** (460 mg, 1.27 mmol) and 1,2-dibromo-1,1,2,2-tetracholoroethane (955 mg, 2.93 mmol) in THF (25 mL), LiHMDS (1.0 M THF solution, 2.55 mL, 2.55 mmol) was added dropwise at 0 °C. The reaction solution was stirred at −78 °C for 30 min. The reaction was quenched with water, and the resulting mixture was extracted with chloroform. The organic layer washed with water three times and was dried over anhydrous sodium sulfate. After the solvent was evaporated under a reduced pressure, the residue was purified by column chromatography on silica gel from hexane:chloroform (1:1) to give **14** as white solid (615 mg,

93%). HRMS Calcd for $C_{18}H_{18}Br_2N_2S_3$ [M+H]$^+$: 516.89989. Found: 516.90759. ^{1}H-NMR (400 MHz, $CDCl_3$, TMS) δ: 7.70 (s, 1H), 2.96 (d, 2H), 3.02 (t, 2H), 1.83 (m, 2H), 1.20–1.50 (m, 10H), 0.87 (t, 3H) ^{13}C-NMR (400 MHz, $CDCl_3$, TMS) δ: 149.09, 145.32, 145.05, 137.59, 136.53, 133.41, 131.25, 125.12, 123.52, 119.04, 31.85, 31.12, 30.72, 29.33, 29.20, 29.07, 22.66, 14.11.

2,5-Dibromo-8-(2-ethylhexyl)thieno[2′,3′:5,6]benzo[1,2-*d*:4,3-*d*′]bisthiazole (15)

To a solution of **13** (200 mg, 0.55 mmol) and 1,2-dibromo-1,1,2,2-tetracholoroethane (465 mg, 1.43 mmol) in THF (10 mL), LiHMDS (1.0 M THF solution, 1.10 mL, 1.10 mmol) was added dropwise at −78 °C. The reaction mixture was stirred at 0 °C for 30 min. The reaction was quenched with water, and the resulting mixture was extracted with chloroform. The organic layer washed with water three times and was dried over anhydrous sodium sulfate. After the solvent was evaporated under a reduced pressure, the residue was purified by column chromatography on silica gel from hexane:chloroform (1:1) to give **15** as white solid (248 mg, 87%). HRMS Calcd for $C_{18}H_{18}Br_2N_2S_3$ [M+H]$^+$: 516.89989. Found: 516.90753. ^{1}H-NMR (400 MHz, $CDCl_3$, TMS) δ: 7.69 (s, 1H), 2.96 (d, 2H), 1.74 (m, 1H), 1.35 (m, 8H), 0.91 (m, 6H) ^{13}C-NMR (400 MHz, $CDCl_3$, TMS) δ: 147.97, 145.47, 145.20, 137.53, 136.46, 133.51, 131.60, 125.24, 123.63, 120.13, 41.49, 34.83, 32.58, 28.92, 25.68, 22.91, 14.02, 10.82.

2,5-Bis(4-(2-butyloctyl)thiophen-2-yl)-8-octylthieno[2′,3′:5,6]benzo[1,2-*d*:4,3-*d*′]bisthiazole (16)

A solution of **14** (300 mg, 0.58 mmol), 3-(2-butyloctyl)-5-trimethylstannylthiophene (598 mg, 1.44 mmol) and tetrakis(triphenylphosphine)palladium(0) (29 mg, 0.026 mmol) in 13 mL of a mixed solvent of toluene and DMF (1:1) was stirred at 90 °C for 4 h. After cooling to room temperature, the reaction was quenched with water and extracted with hexane. The organic layer was washed with water three times and was dried over anhydrous sodium sulfate. After the solvent was evaporated under a reduced pressure, the crude product was purified by column chromatography on silica gel with hexane:ethyl acetate (30:1) to give **16** as yellow oil (490 mg, 98%). HRMS Calcd for $C_{50}H_{72}N_2S_5$ [M+H]$^+$:861.42990. Found: 861.43561. ^{1}H-NMR (400 MHz, $CDCl_3$, TMS) δ: 7.77 (s, 1H), 7.50 (dd, 2H), 7.08 (s, 2H), 3.04 (t, 2H), 2.59 (d, 4H), 1.85 (m, 2H), 1.66 (m, 2H), 1.20–1.50 (m, 42H), 0.80–0.95 (m, 15H) ^{13}C-NMR (400 MHz, $CDCl_3$, TMS) δ: 160.81, 159.94, 147.69, 146.77, 146.27, 143.27, 136.67, 136.42, 133.77, 131.42, 129.96, 129.81, 125.15, 124.86, 123.22, 121.80, 119.71, 38.89, 34.92, 33.29, 32.96, 31.91,

31.88, 31.40, 30.81, 29.70, 29.40, 29.24, 29.14, 28.85, 26.59, 23.05, 22.70, 22.68, 14.16, 14.14, 14.12.

8-(2-Ethylhexyl)-2,5-bis(4-(2-ethylhexyl)thiophen-2-yl)thieno[2′,3′:5,6]benzo[1,2-*d*:4,3-*d*′]bisthiazole (17)

A solution of **15** (248 mg, 0.48 mmol), 3-(2-ethylhexyl)-5-trimethylstannylthiophene (433 mg, 1.20 mmol) and tetrakis(triphenylphosphine)palladium(0) (24 mg, 0.021 mmol) in 10 mL of a mixed solvent of toluene and DMF (1:1) was stirred at 90 °C for 4 h. After cooling to room temperature, the reaction was quenched with water and was extracted with hexane. The organic layer was washed with water three times and was dried over anhydrous sodium sulfate. After the solvent was evaporated under a reduced pressure, the crude product was purified by column chromatography on silica gel with hexane:ethyl acetate (30:1) to give **17** as yellow oil (220 mg, 62%). HRMS Calcd for $C_{42}H_{56}N_2S_5$ $[M+H]^+$: 749.3047. Found: 749.31244. ^{1}H-NMR (400 MHz, $CDCl_3$, TMS) δ: 7.75 (s, 1H), 7.51 (dd, 2H), 7.09 (s, 2H), 2.98 (d, 2H), 2.59 (d, 4H), 1.79 (m, 1H), 1.61 (m, 2H), 1.35–1.45 (m, 24H), 0.80–1.00 (m, 18H) ^{13}C-NMR (400 MHz, $CDCl_3$, TMS) δ: 160.82, 159.93, 146.72, 146.59, 146.22, 143.28, 136.67, 136.43, 133.71, 131.60, 129.93, 129.79, 125.14, 124.84, 123.22, 121.79, 120.74, 41.63, 40.32, 34.97, 34.50, 32.60, 32.44, 31.60, 29.00, 28.87, 25.61, 23.05, 23.02, 22.66, 14.16, 14.13, 10.87, 10.85.

2,5-Bis(5-bromo-4-(2-butyloctyl)thiophen-2-yl)-8-octylthieno[2′,3′:5,6]benzo[1,2-*d*:4,3-*d*′]bisthiazole (18)

To a solution of **16** (490 mg, 0.56 mmol) in chloroform (20 mL), NBS (200 mg, 1.13 mmol) was added at 0 °C. The reaction mixture was then stirred at 0 °C for 2 h, and water was added. The mixture was extracted with chloroform, and the organic layer was washed with water three times. The organic layer was dried over anhydrous sodium sulfate, and the solvent was evaporated under a reduced pressure. The crude product was purified by column chromatography on silica gel with hexane:chloroform (3:1) to give **18** as yellow solid (450 mg, 80%). HRMS Calcd for $C_{50}H_{70}Br_2N_2S_5$ $[M+H]^+$: 1017.25093. Found 1017.25745. ^{1}H-NMR (400 MHz, $CDCl_3$, TMS) δ: 7.70 (s, 1H), 7.29 (s, 1H), 7.28 (s, 1H), 3.03 (t, 2H), 2.54 (d, 4H), 1.85 (m, 2H), 1.72 (m, 2H), 1.20–1.50 (m, 42H), 0.85–0.95 (m, 15H) ^{13}C-NMR (400 MHz, $CDCl_3$, TMS) δ: 159.69, 158.84, 148.03, 146.72, 146.23, 136.41, 136.16, 133.81, 131.47, 129.11, 128.96, 123.05, 121.61, 119.60, 114.77, 114.43, 38.55, 34.26, 33.33, 33.02, 31.89, 31.88, 31.38, 30.82, 29.69, 29.40, 29.24, 29.17, 28.76, 26.51, 23.05, 22.69, 22.67, 14.13, 14.11.

2,5-Bis(5-bromo-4-(2-ethylhexyl)thiophen-2-yl)-8-(2-ethylhexyl)thieno[2′,3′:5,6]benzo[1,2-*d*:4,3-*d*′]bisthiazole (19)

To a solution of **19** (100 mg, 0.13 mmol) in chloroform (6.0 mL), NBS (48 mg, 0.26 mmol) was added at 0 °C. The reaction mixture stirred for 2 h, and water was added. The mixture was extracted with chloroform, and the organic layer was washed with water three times. The organic layer was dried over anhydrous sodium sulfate, and the solvent was evaporated under a reduced pressure. The crude product was purified by column chromatography on silica gel with hexane:chloroform (3:1) and recrystallized from hexane:ethanol (5:1) to give **19** as yellow solid (70 mg, 62%). HRMS Calcd for $C_{42}H_{54}Br_2N_2S_5$ $[M+H]^+$: 905.12573. Found: 905.13324. ^{1}H-NMR (400 MHz, $CDCl_3$, TMS) δ: 7.71 (s, 1H), 7.33 (s, 1H), 7.32 (s, 1H), 2.98 (d, 2H), 2.55 (d, 4H), 1.78 (m, 1H), 1.68 (m, 2H), 1.30–1.50 (m, 24H), 0.88–0.98 (m, 18H) ^{13}C-NMR (400 MHz, $CDCl_3$, TMS) δ:159.67, 158.81, 146.89, 146.63, 146.15, 142.62, 136.41, 136.17, 133.73, 131.64, 129.07, 128.93, 123.02, 121.57, 120.64, 114.78, 114.43, 41.61, 39.97, 34.96, 33.86, 32.59, 32.45, 28.98, 28.78, 25.67, 25.63, 23.06, 23.03, 14.18, 14.15, 10.88, 10.84.

PiNBTz1

To a reaction tube equipped with a stirring bar, **7** (42.6 mg, 0.05 mmol), 2,6-bis(trimethytin)-4,8-bis(5-(2-ethylhexyl)thiophen-2-yl)benzo[1,2-*b*:4,5-*b*′]dithiophene (45.3 mg, 0.05 mmol), tetrakis(triphenylphosphine)palladium(0) (1.1 mg, 0.001 mmol), and toluene (2.0 mL) were added. The tube was purged with argon and sealed, and then was heated at 200 °C for 2 h in a microwave reactor. After cooling room temperature, the reaction solution was poured into 100 mL of methanol containing 5 mL of hydrochloric acid, and stirred for 5 h. Then the precipitated solid was subjected to the sequential Soxhlet extraction with methanol, hexane, dichloromethane to remove low molecular weight fractions. The residue was then extracted with chloroform, and reprecipitated in 100 mL of methanol to yield dark red solids (60 mg, 95%). Anal. Calcd for $C_{74}H_{96}N_2S_8$: C, 69.98; H, 7.62; N, 2.21. Found: C, 69.86; H, 6.95; N, 2.27.

PTBTz1

To a reaction tube equipped with a stirring bar, **18** (50.9 mg, 0.05 mmol), 2,6-bis(trimethytin)-4,8-bis(5-(2-ethylhexyl)thiophen-2-yl)benzo[1,2-*b*:4,5-*b*′]dithiophene (45.3 mg, 0.05 mmol), tetrakis(triphenylphosphine)palladium(0) (1.1 mg, 0.001 mmol), and toluene (2.0 mL) were added. The tube was purged with argon and sealed, and then was heated at 200 °C for 2 h in microwave reactor. After cooling room temperature, the reaction solution was poured

into 100 mL of methanol containing 5.0 mL of hydrochloric acid, and stirred for 5 h. Then the precipitated solid was subjected to the sequential Soxhlet extraction with methanol, hexane, dichloromethane to remove low molecular weight fractions. The residue was then extracted with chloroform, and reprecipitated in 100 mL of methanol to yield dark red solids (67 mg, 93%). Anal. Calcd for $C_{84}H_{112}N_2S_9$: C, 70.43, H, 7.85, N, 1.95. Found: C, 70.45, H, 7.82, N, 2.01.

PTBTz2

To a reaction tube equipped with a stirring bar, **19** (45.3 mg, 0.05 mmol), 2,6-bis(trimethytin)-4,8-bis(5-(2-ethylhexyl)thiophen-2-yl)benzo[1,2-*b*:4,5-*b'*]dithiophene (45.3 mg, 0.05 mmol), tetrakis(triphenylphosphine)palladium(0) (1.1 mg, 0.001 mmol), and toluene (2.0 mL) were added. The tube was purged with argon and sealed, and then was heated at 200 °C for 2 h in microwave reactor. After cooling room temperature, the reaction solution was poured into 100 mL of methanol containing 5.0 mL of hydrochloric acid, and stirred for 5 h. Then the precipitated solid was subjected to the sequential Soxhlet extraction with methanol, hexane, dichloromethane to remove low molecular weight fractions. The residue was then extracted with chloroform, and reprecipitated in 100 mL of methanol to yield dark red solids (59 mg, 89%). Anal. Calcd for $C_{78}H_{100}N_2S_9$: C, 69.18, H, 7.74, N 2.07. Found: C, 68.00, H, 7.21, N, 2.20.

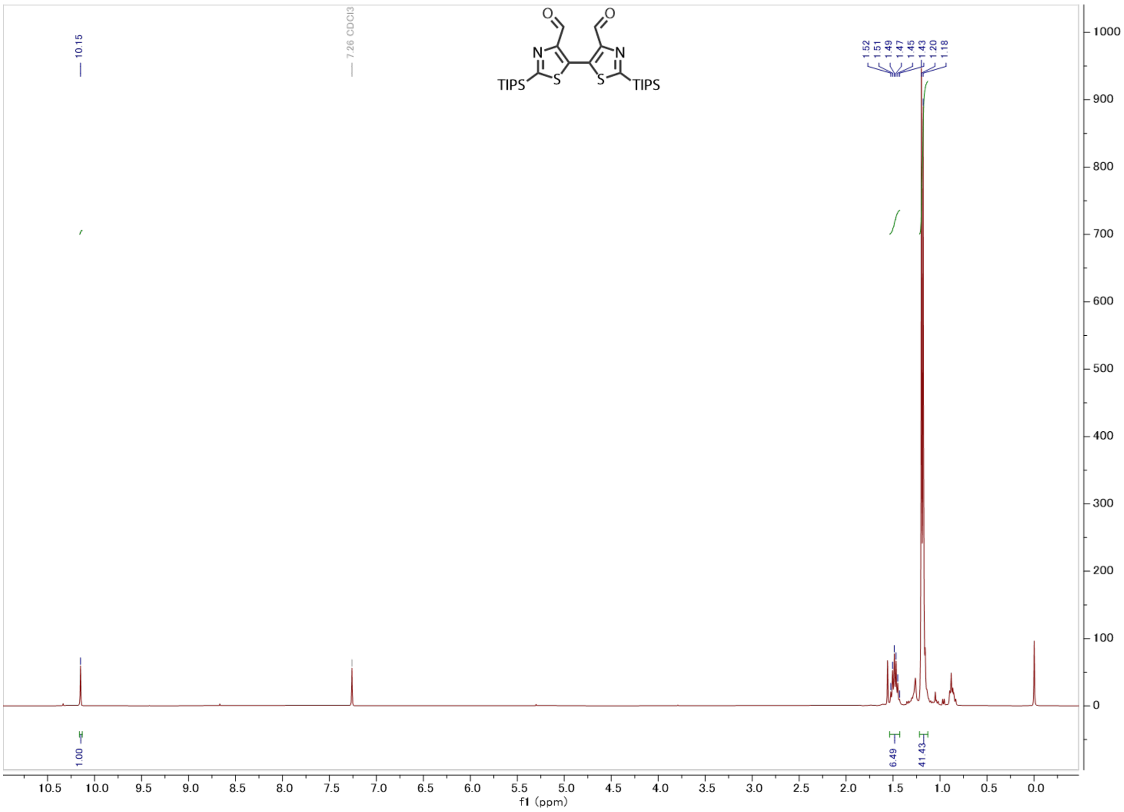

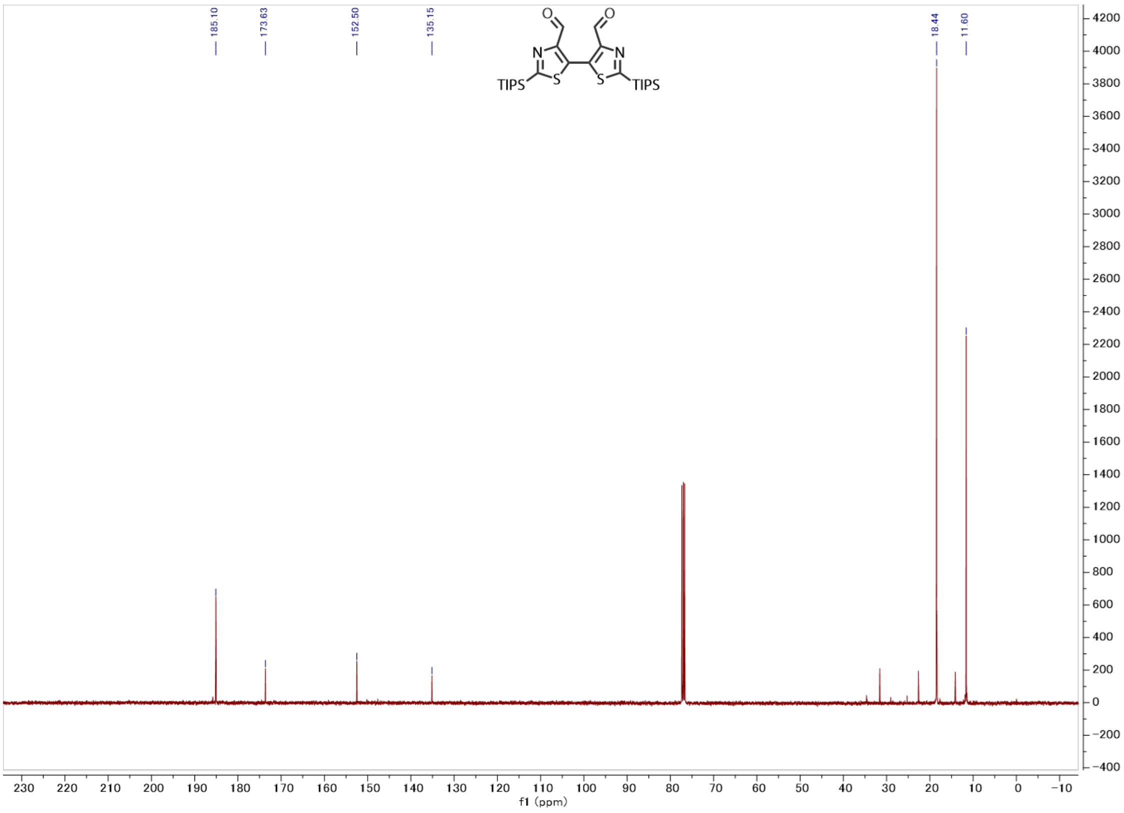

NMR (upper) and ^{13}C-NMR (lower) spectra of **2**

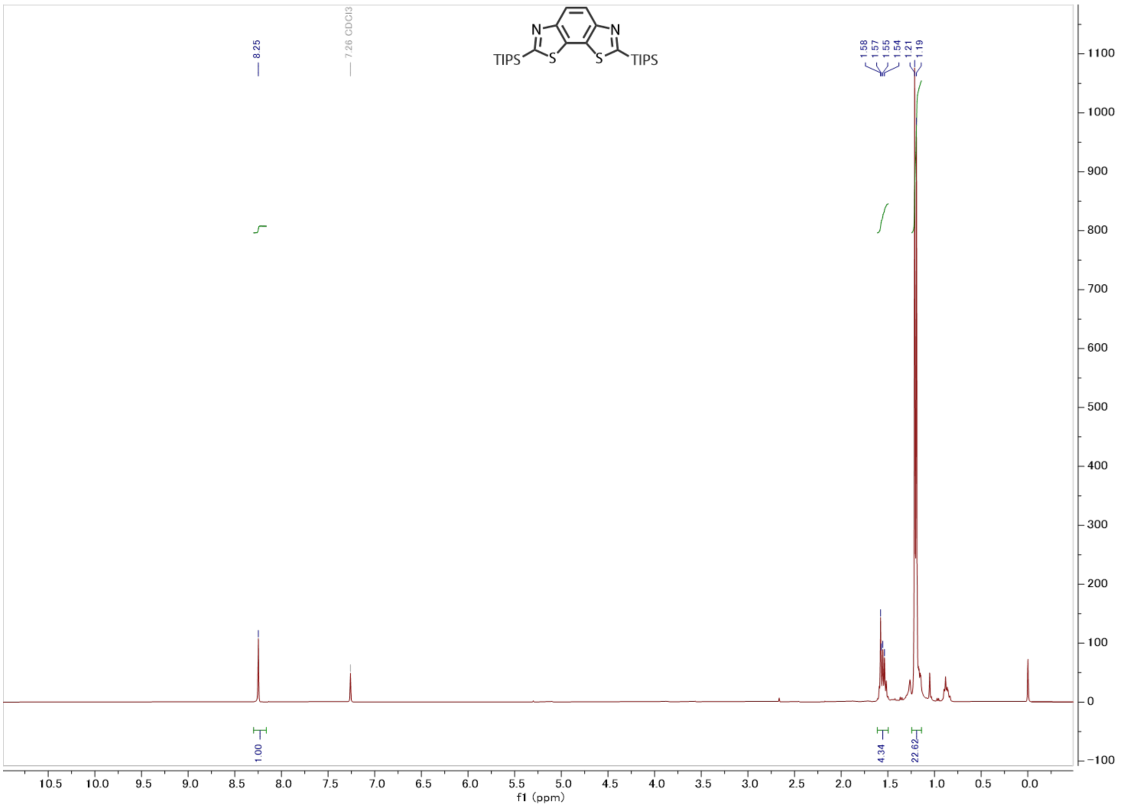

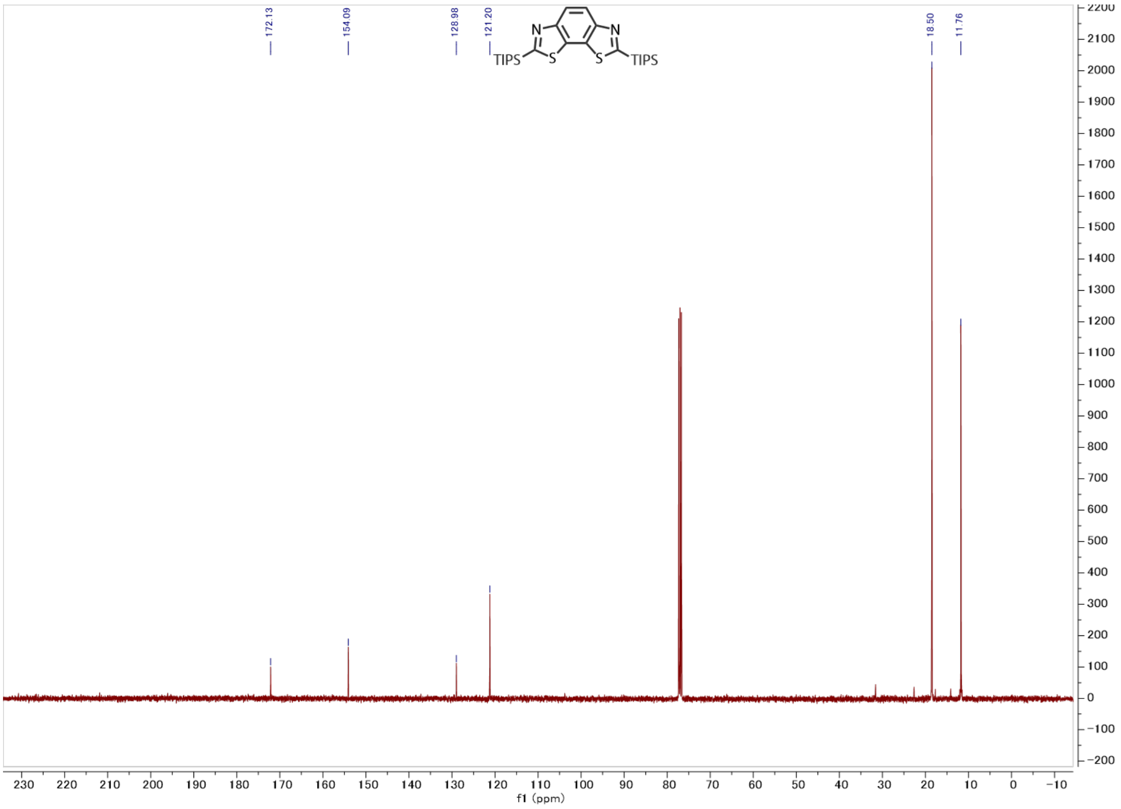

^{1}H-NMR (upper) and ^{13}C-NMR (lower) spectra of **3**

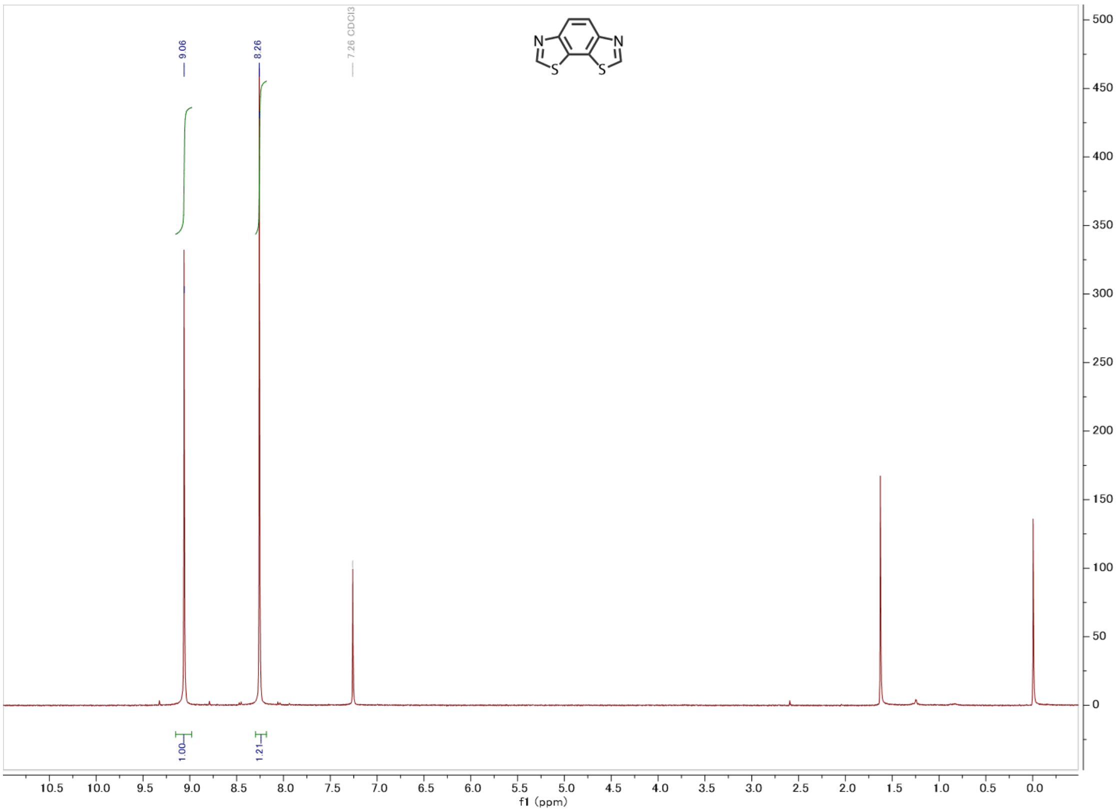

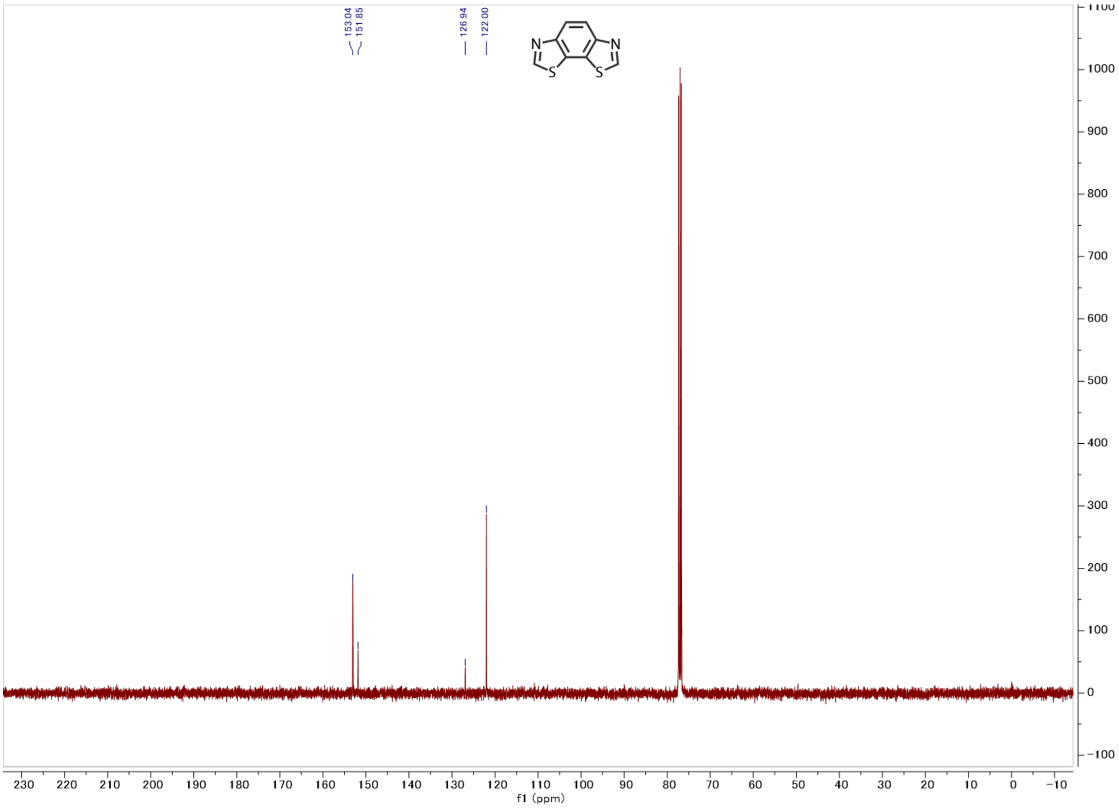

^{1}H-NMR (upper) and ^{13}C-NMR (lower) spectra of **4**

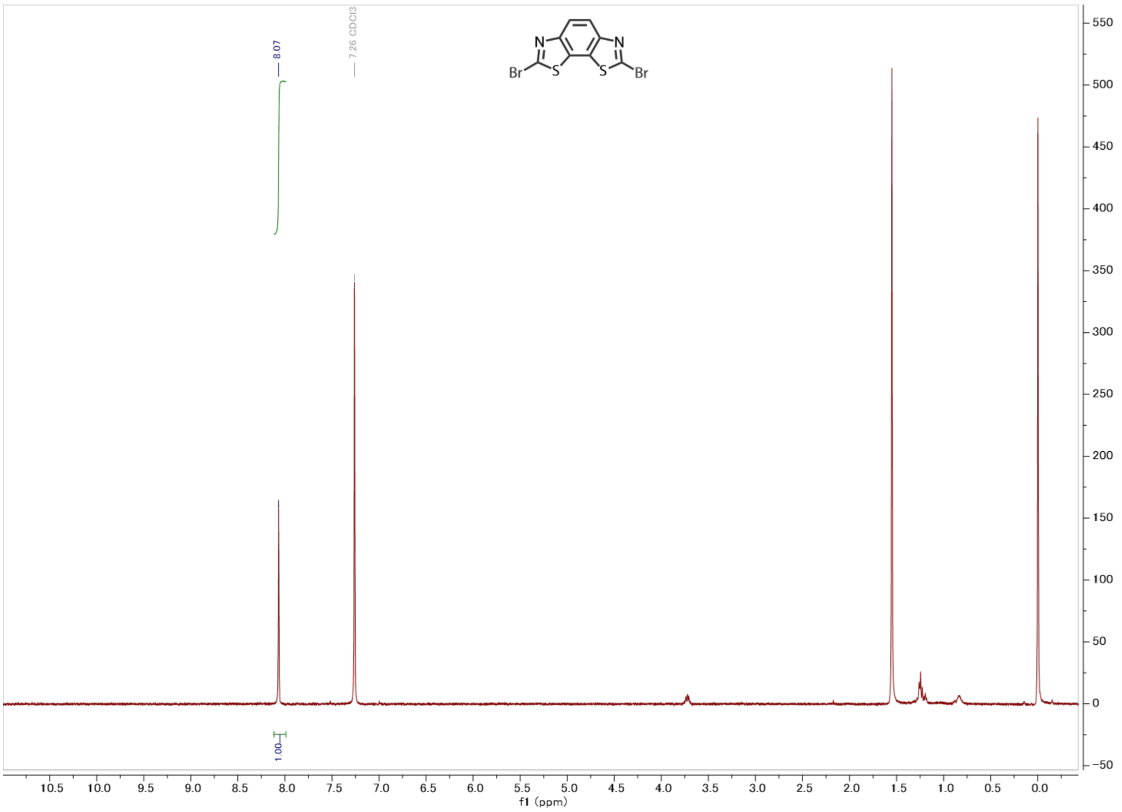

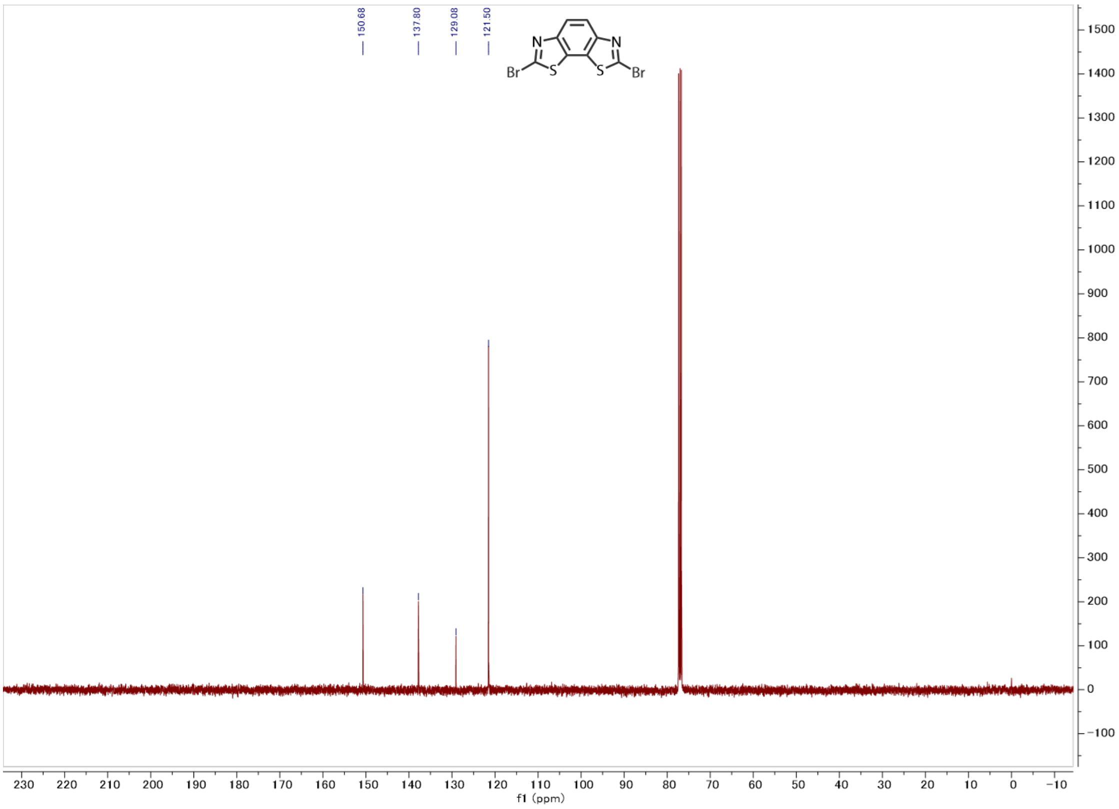

^{1}H-NMR (upper) and ^{13}C-NMR (lower) spectra of **5**

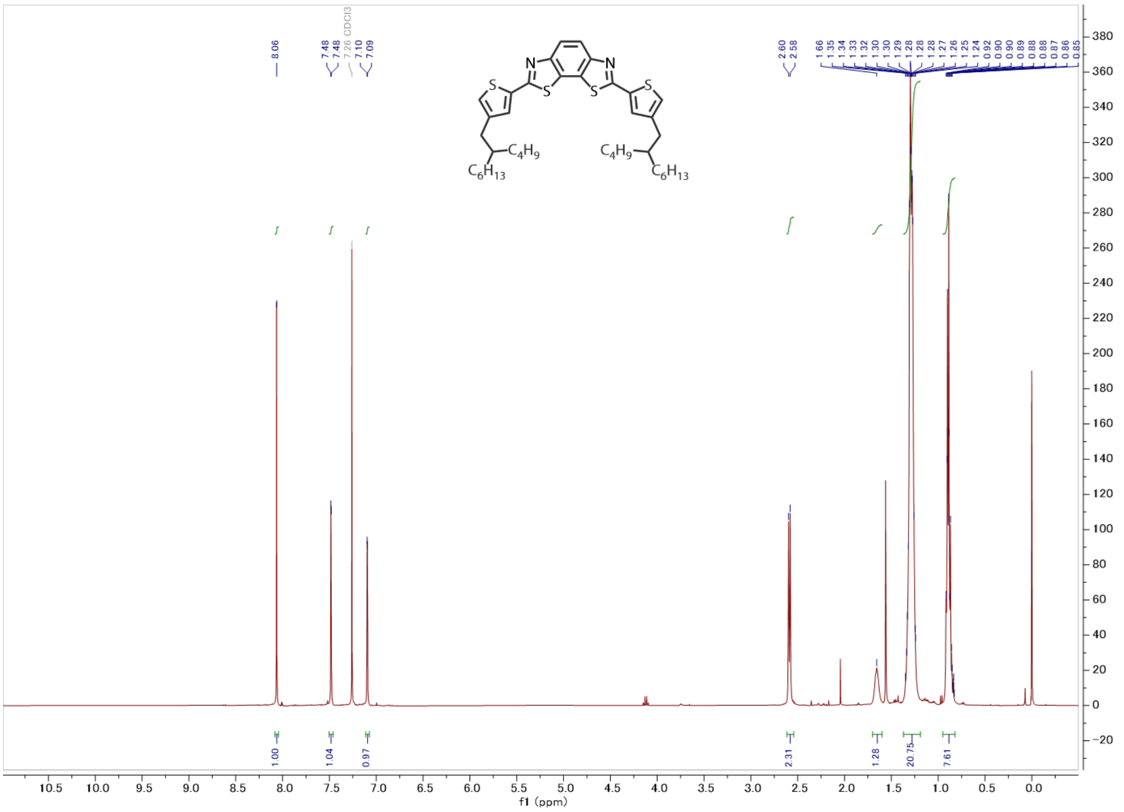

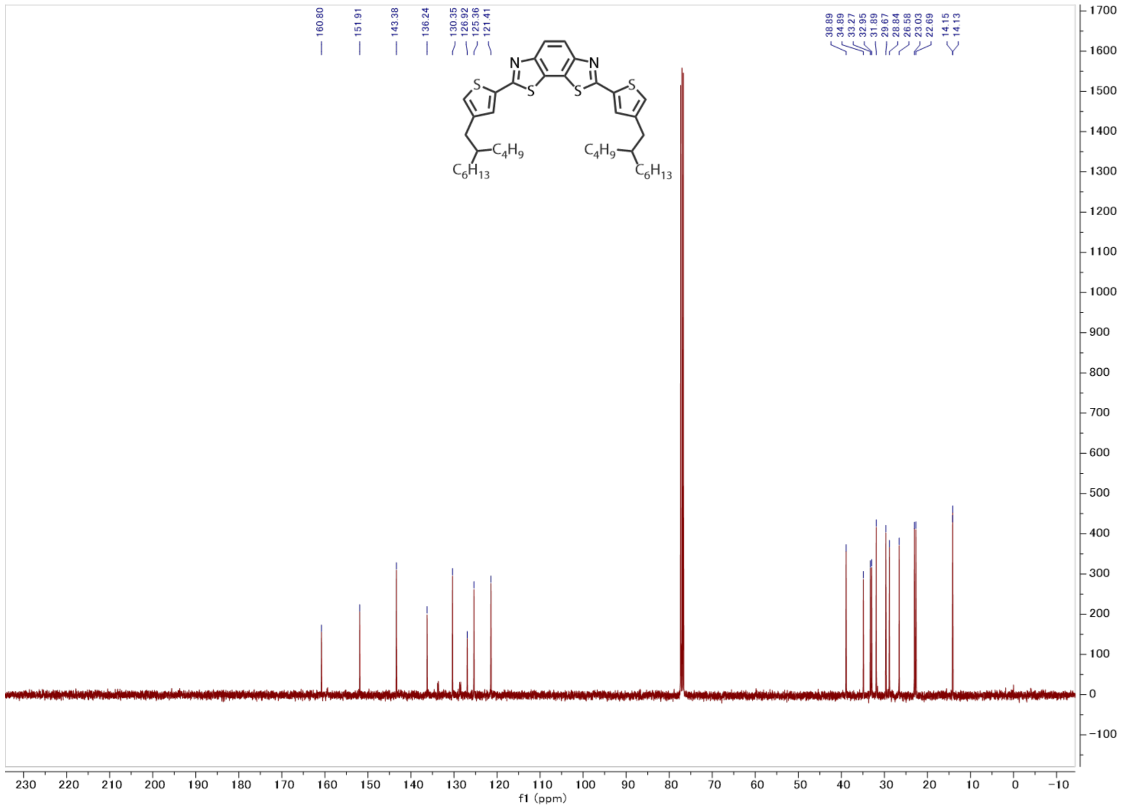

^{1}H-NMR (upper) and ^{13}C-NMR (lower) spectra of **6**

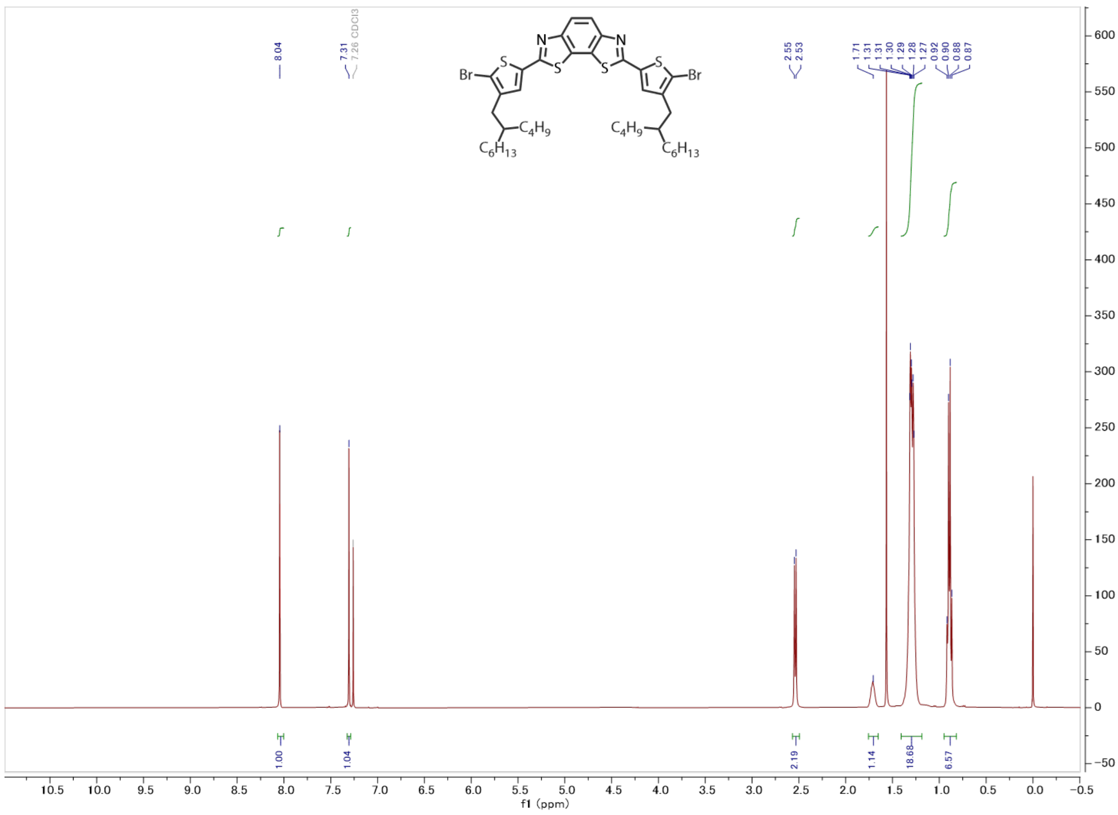

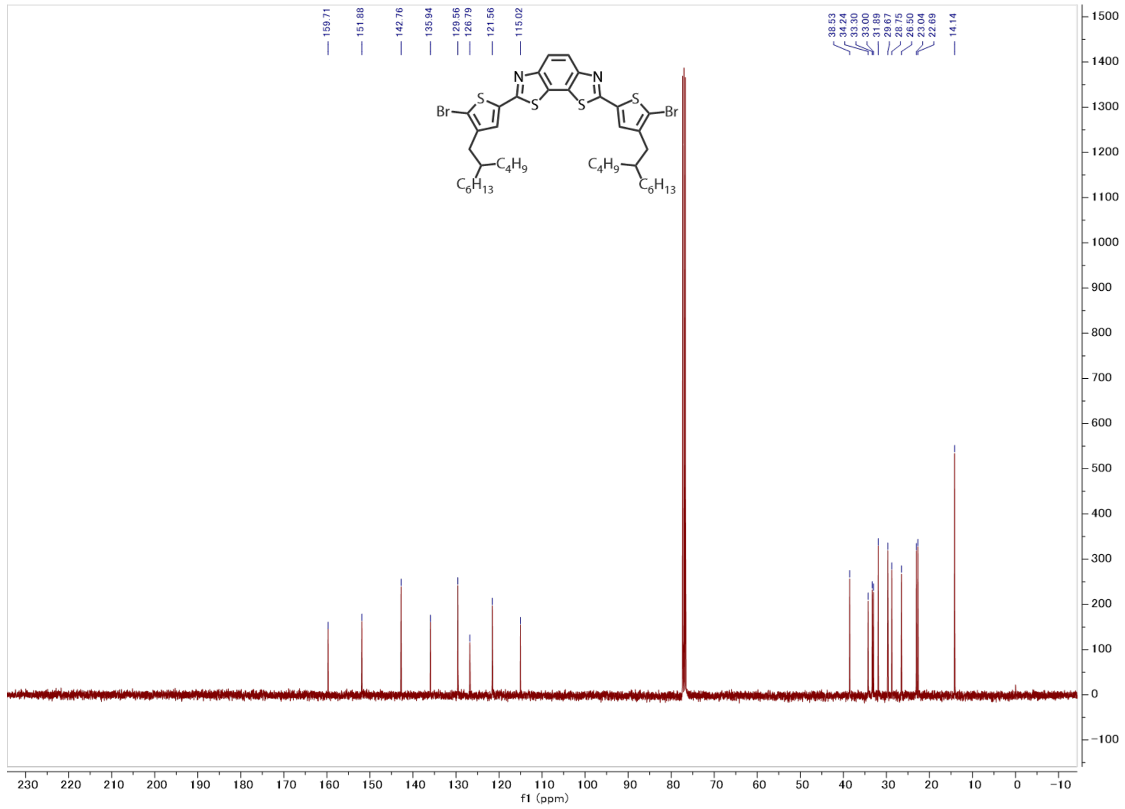

^{1}H-NMR (upper) and ^{13}C-NMR (lower) spectra of **7**

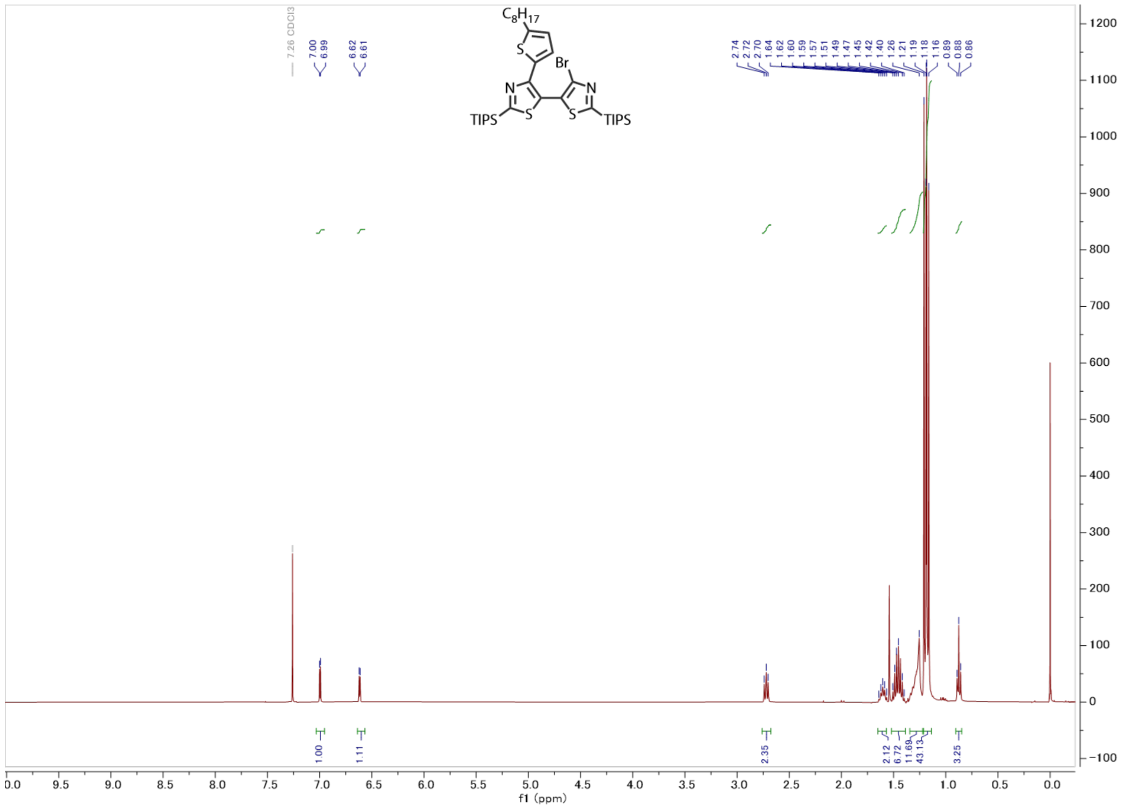

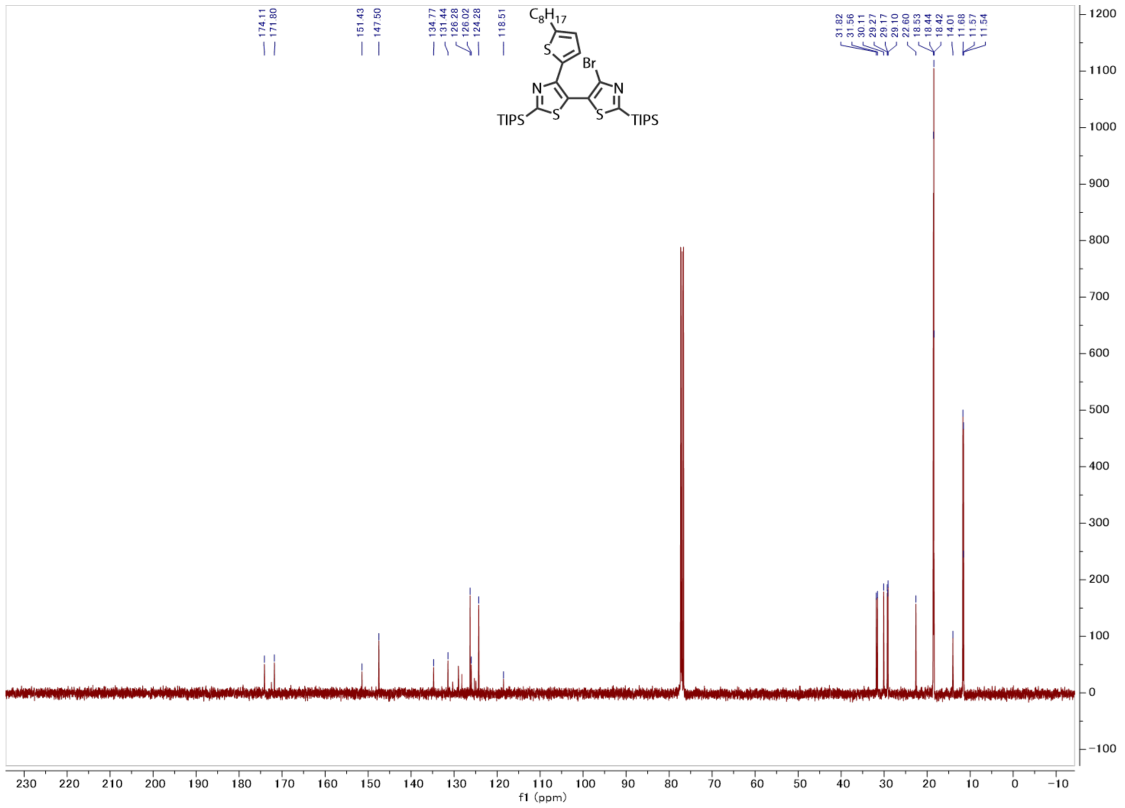

^{1}H-NMR (upper) and ^{13}C-NMR (lower) spectra of **8**

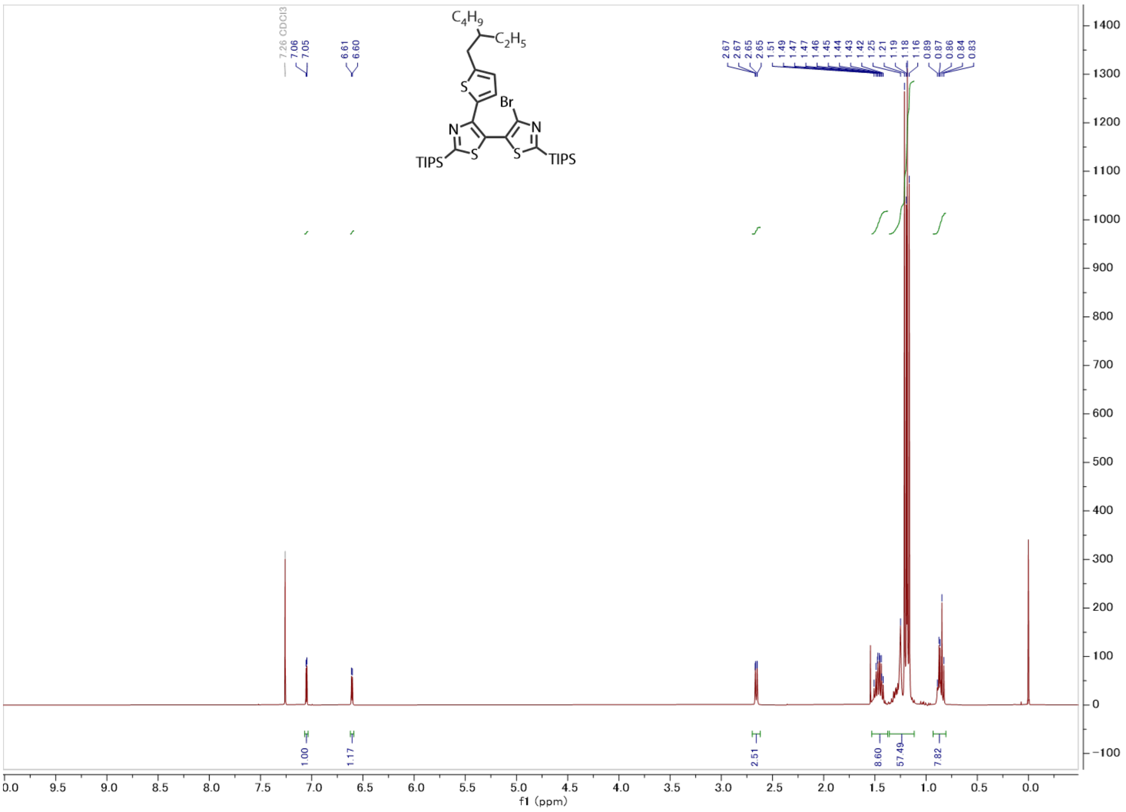

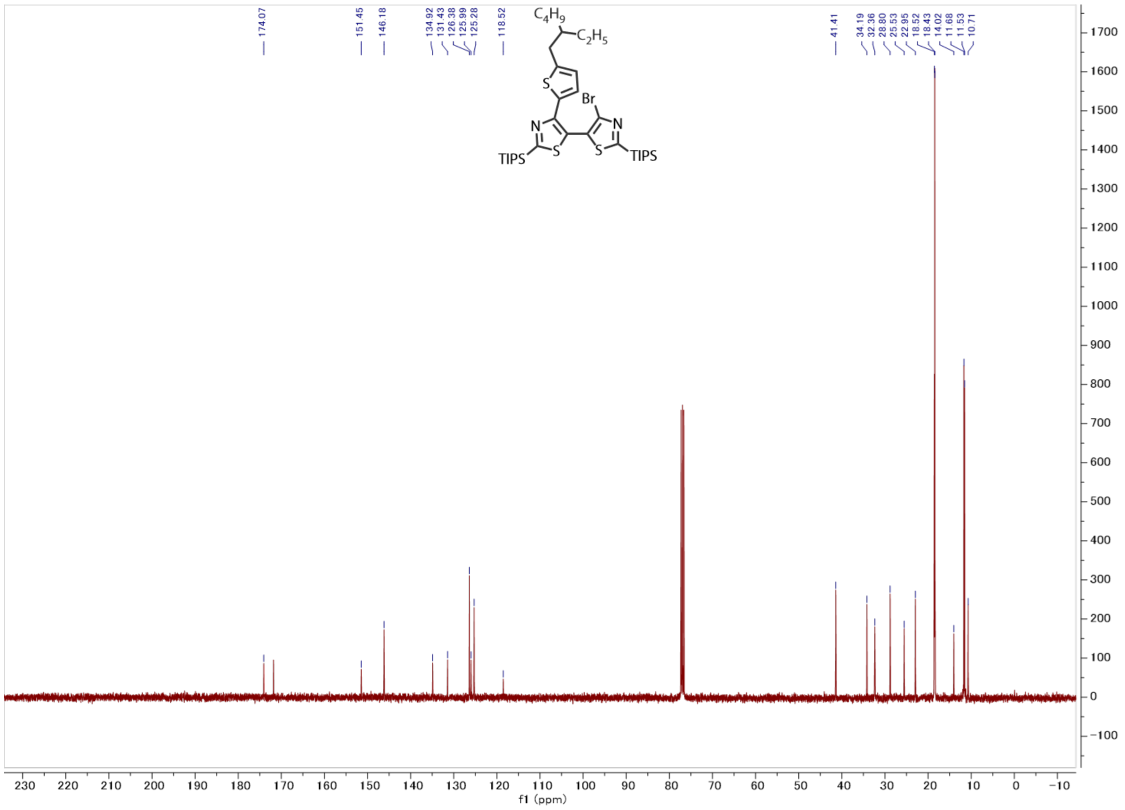

^{1}H-NMR (upper) and ^{13}C-NMR (lower) spectra of **9**

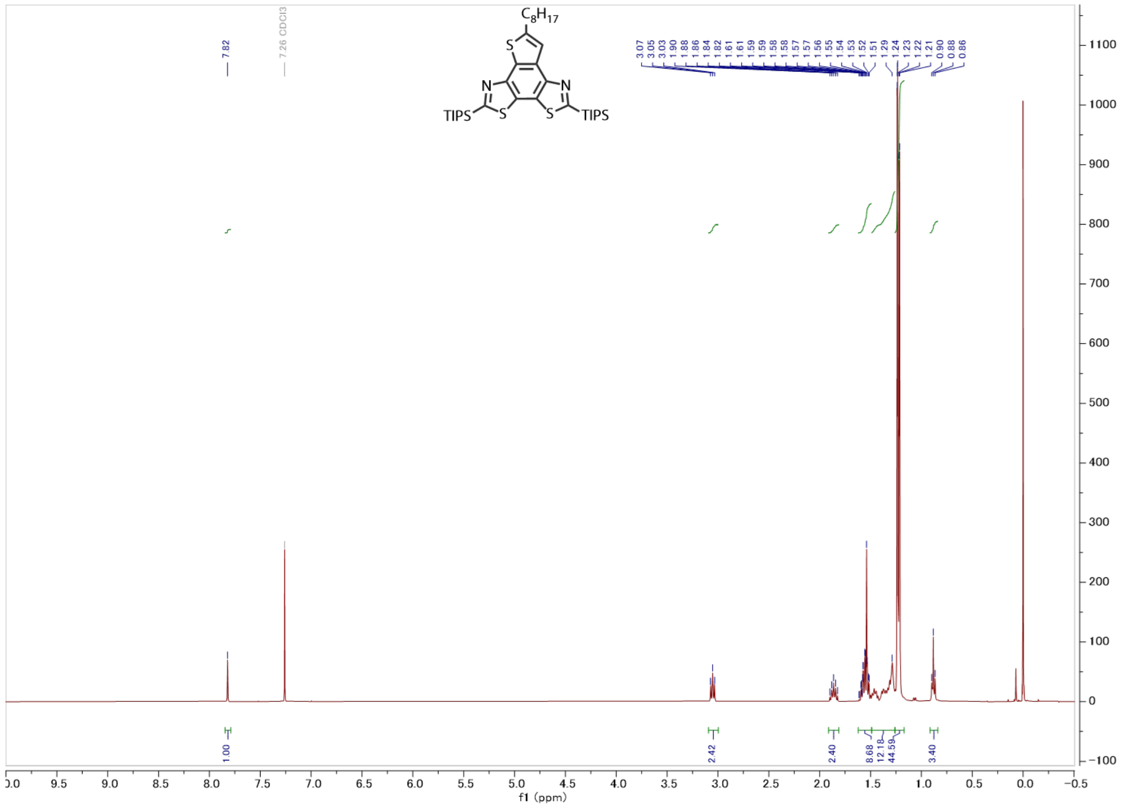

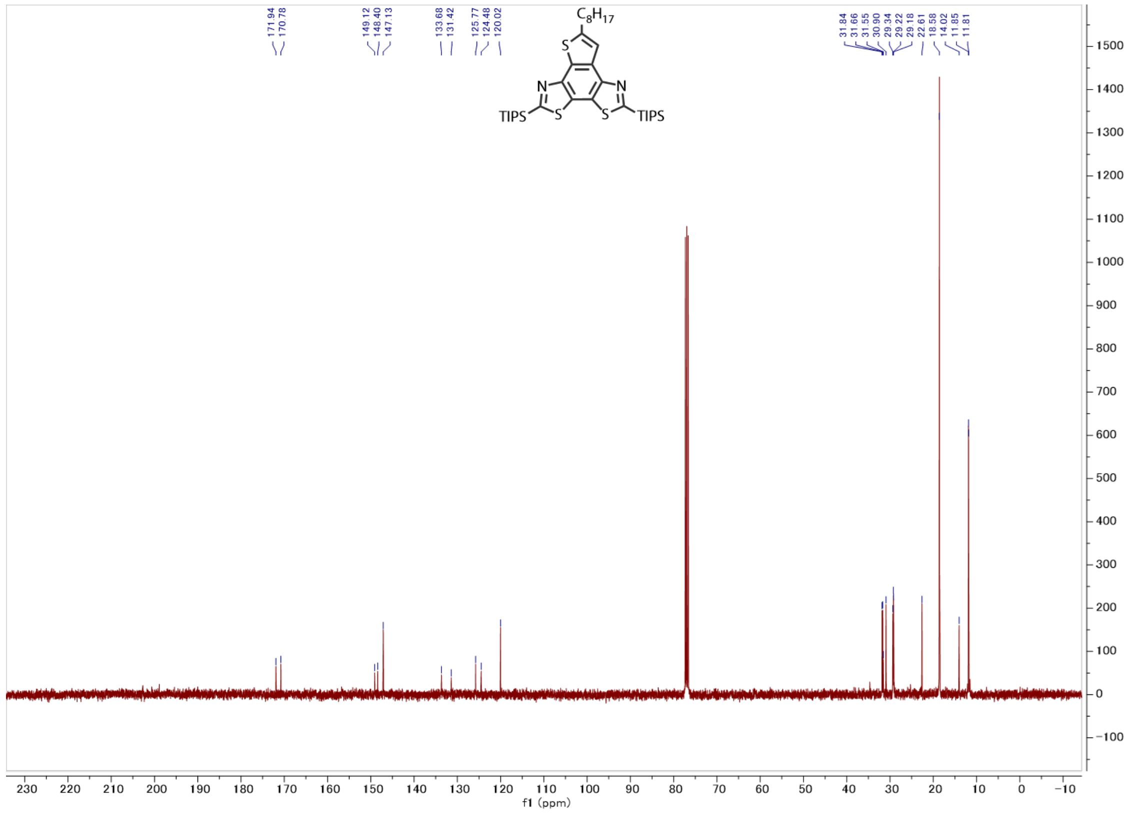

^{1}H-NMR (upper) and ^{13}C-NMR (lower) spectra of **10**

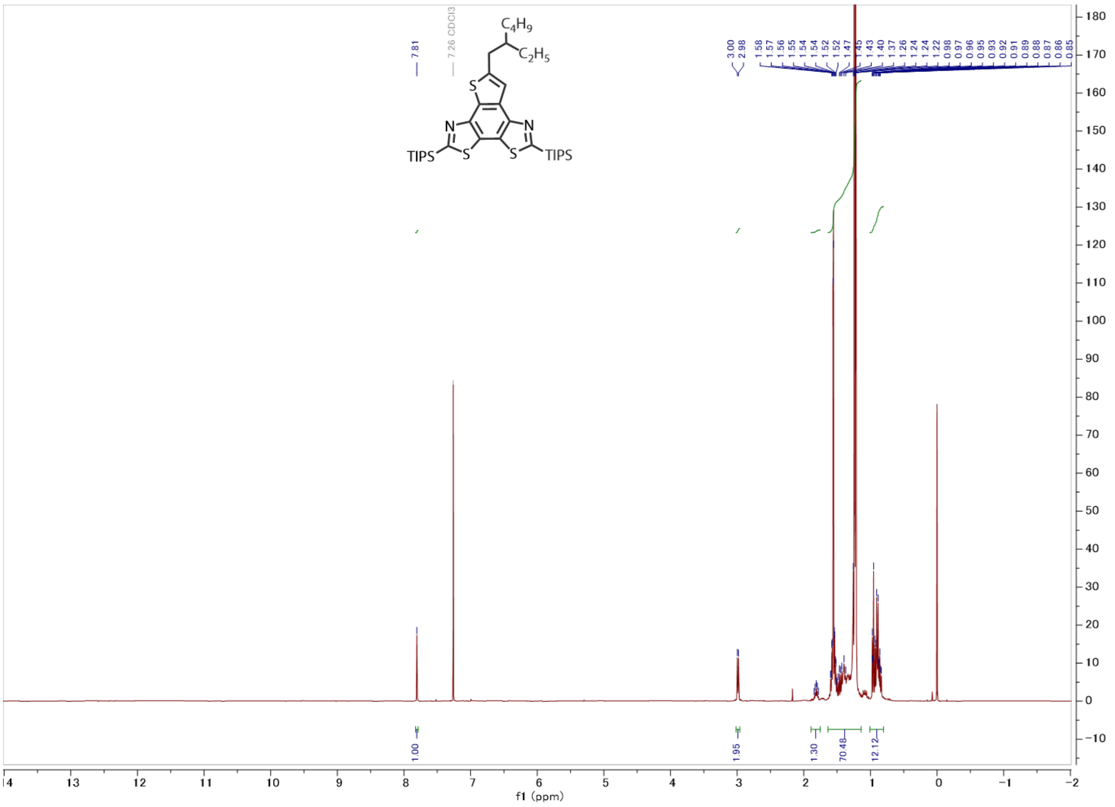

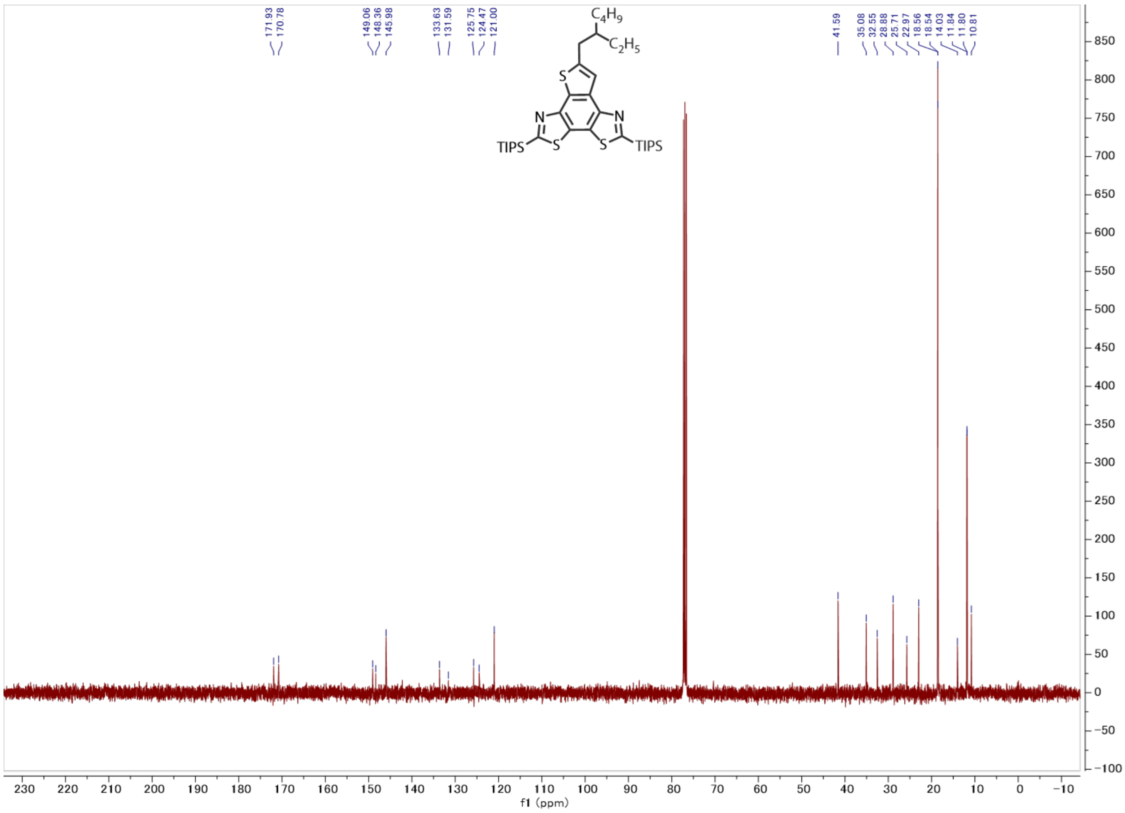

^{1}H-NMR (upper) and ^{13}C-NMR (lower) spectra of **11**

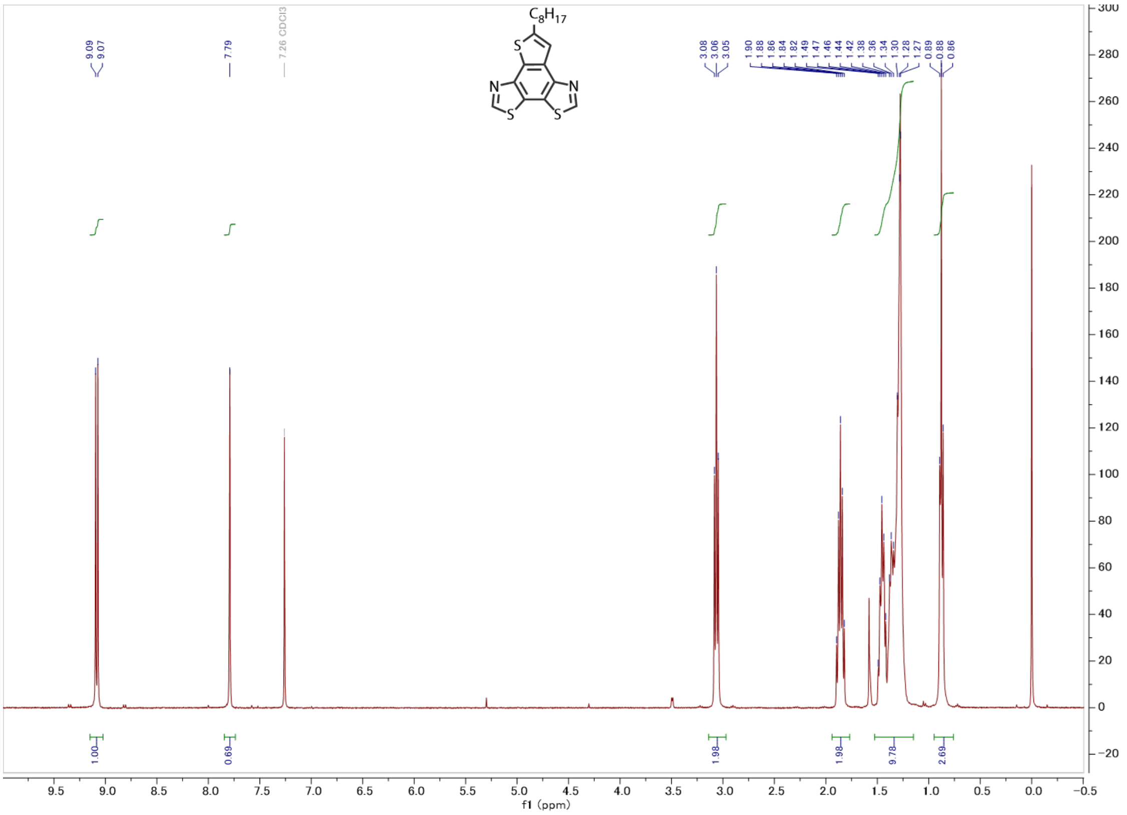

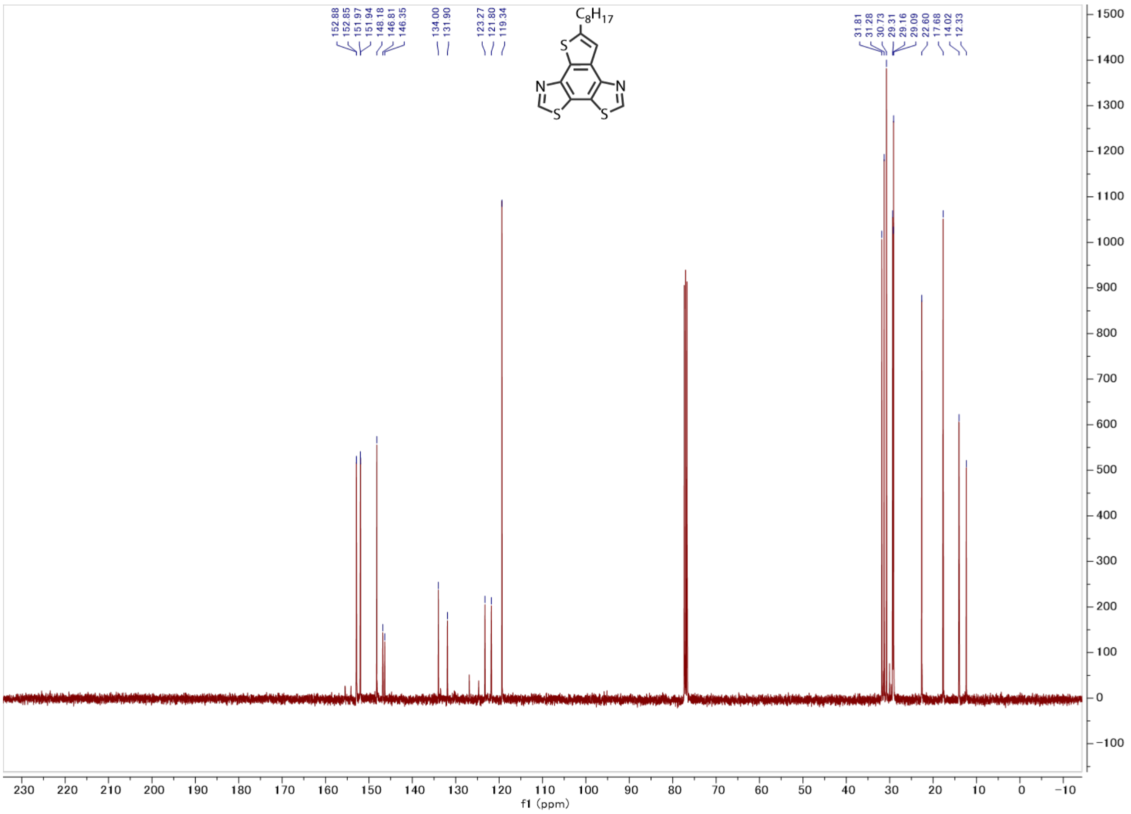

^{1}H-NMR (upper) and ^{13}C-NMR (lower) spectra of **12**

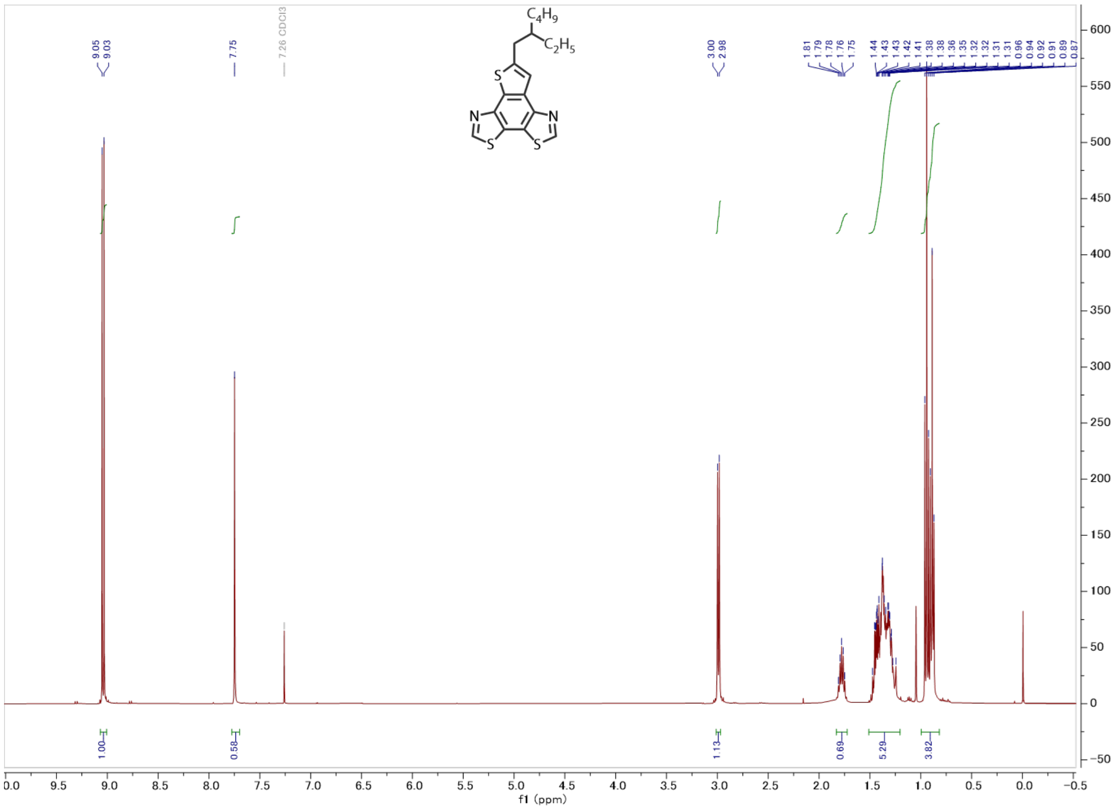

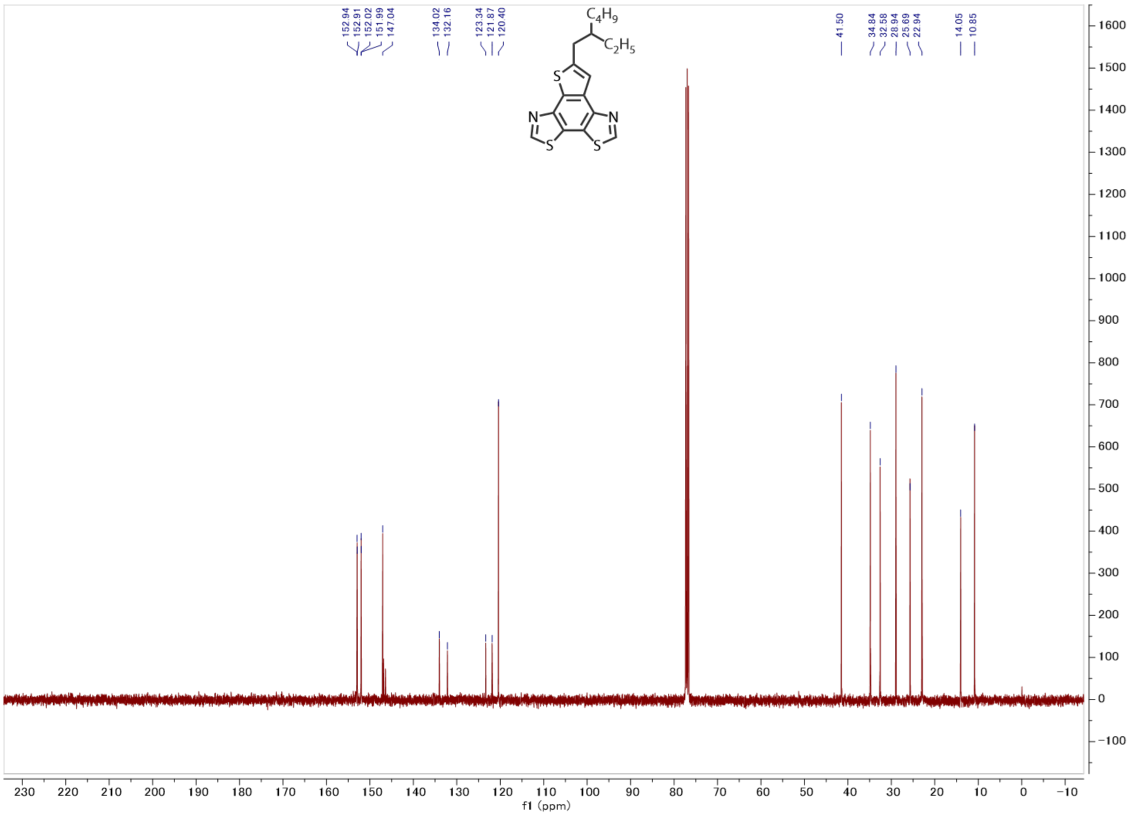

^{1}H-NMR (upper) and ^{13}C-NMR (lower) spectra of **13**

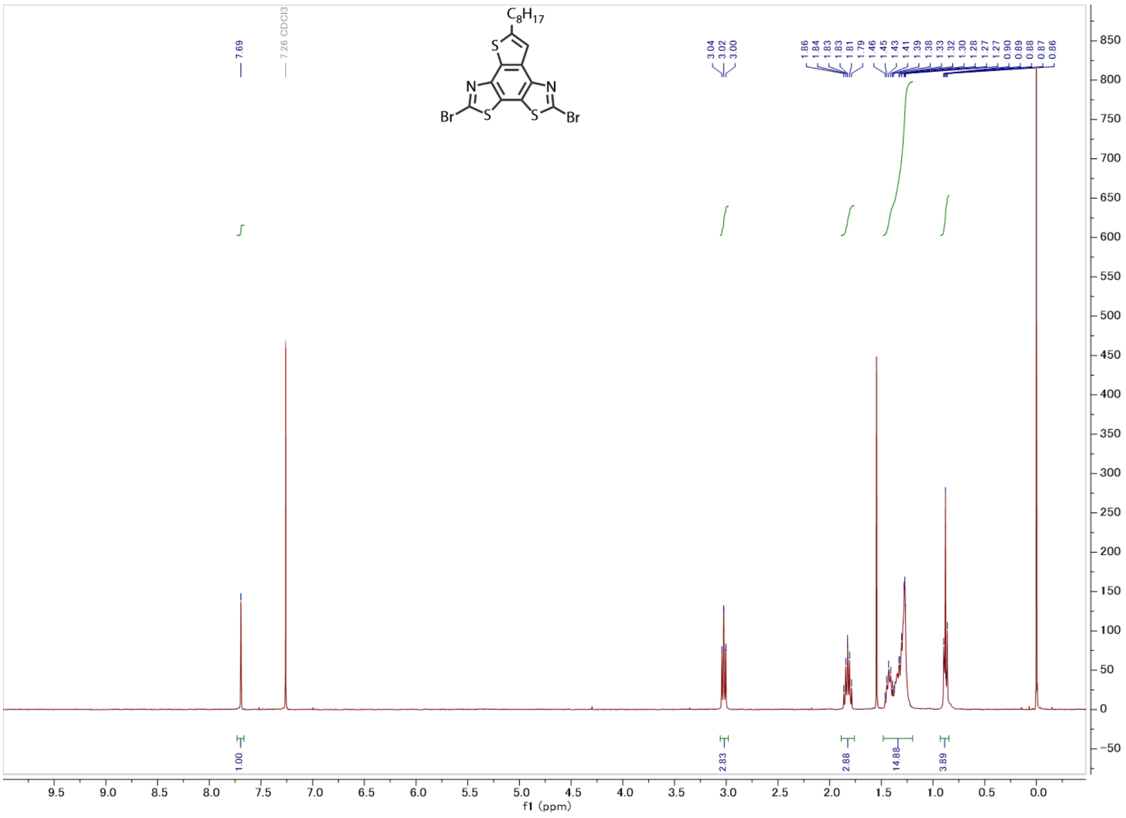

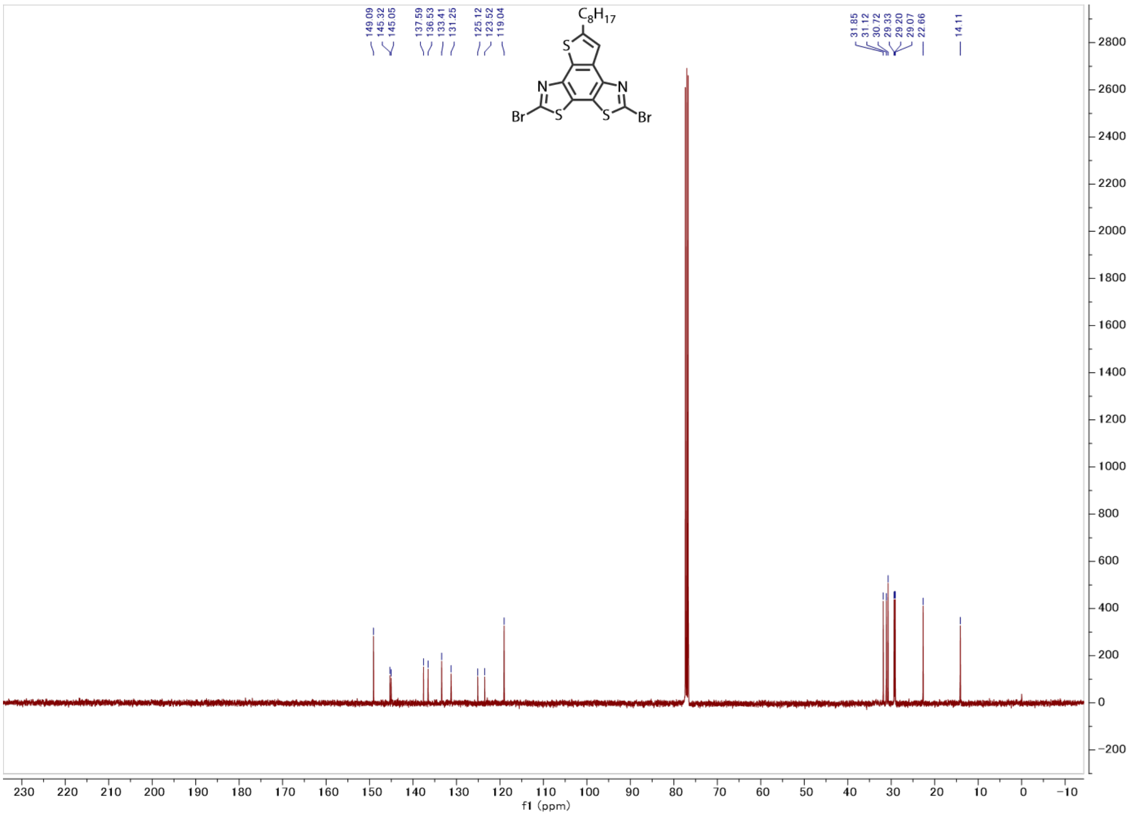

^{1}H-NMR (upper) and ^{13}C-NMR (lower) spectra of **14**

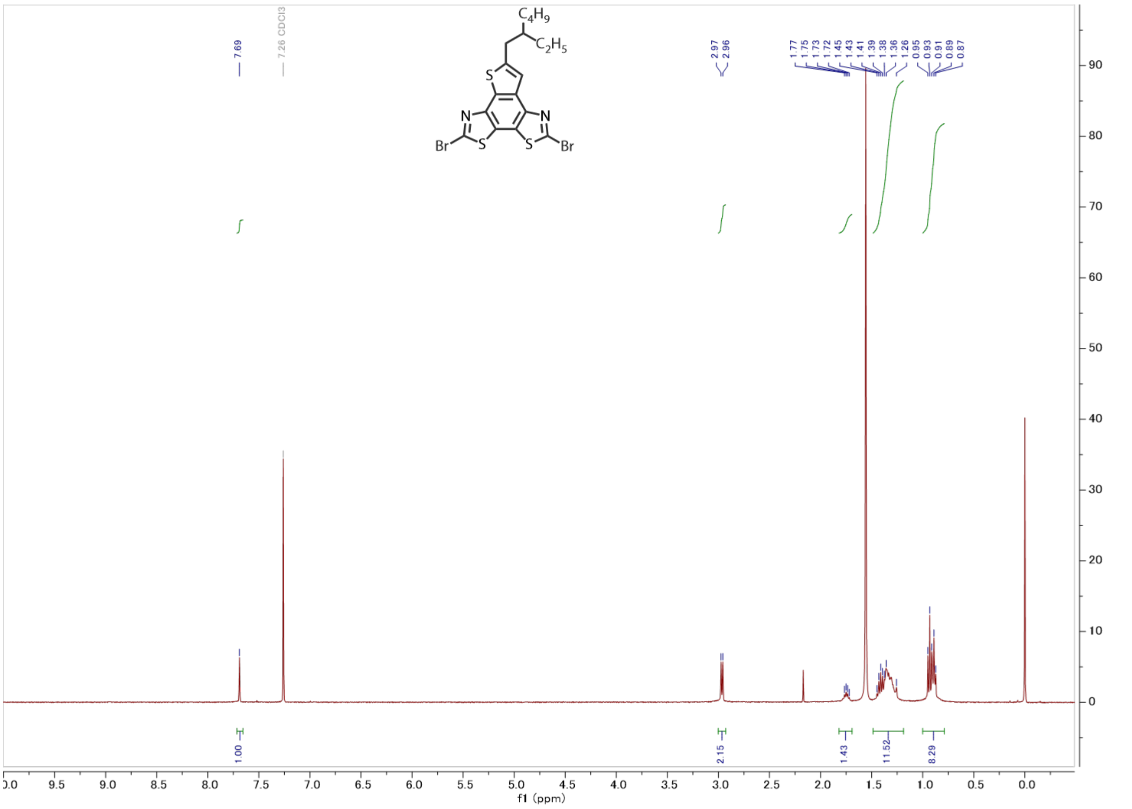

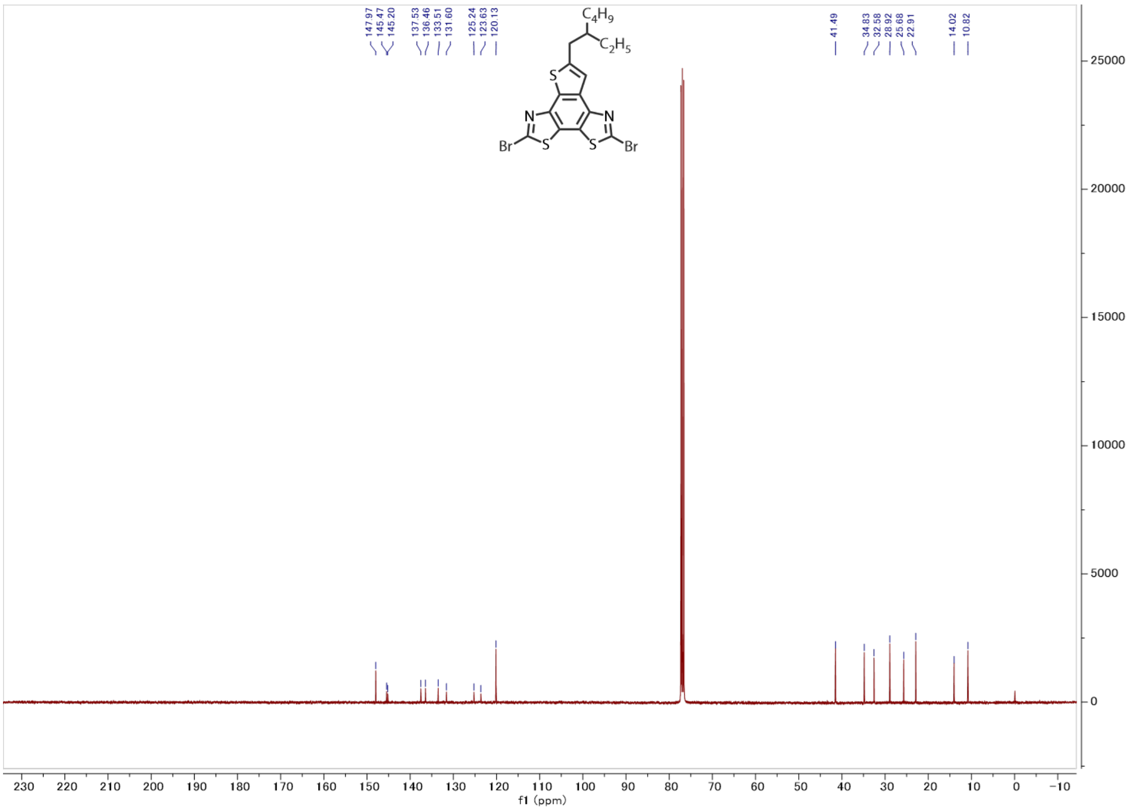

^{1}H-NMR (upper) and ^{13}C-NMR (lower) spectra of **15**

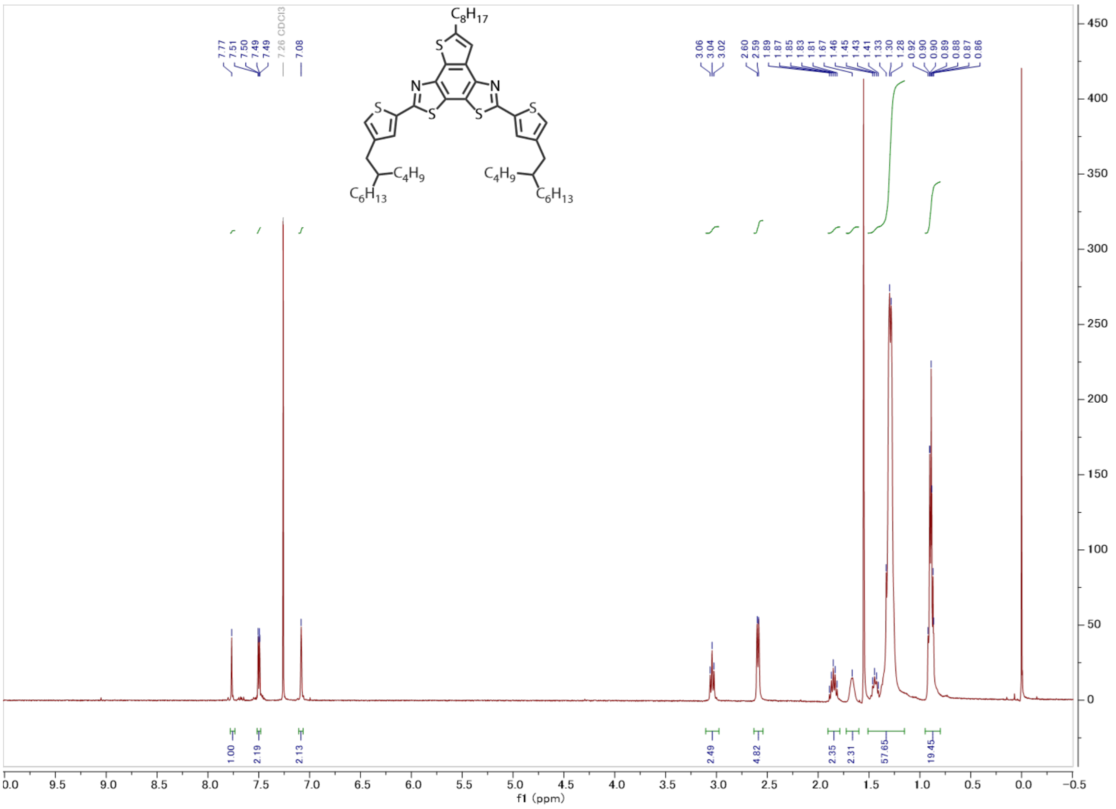

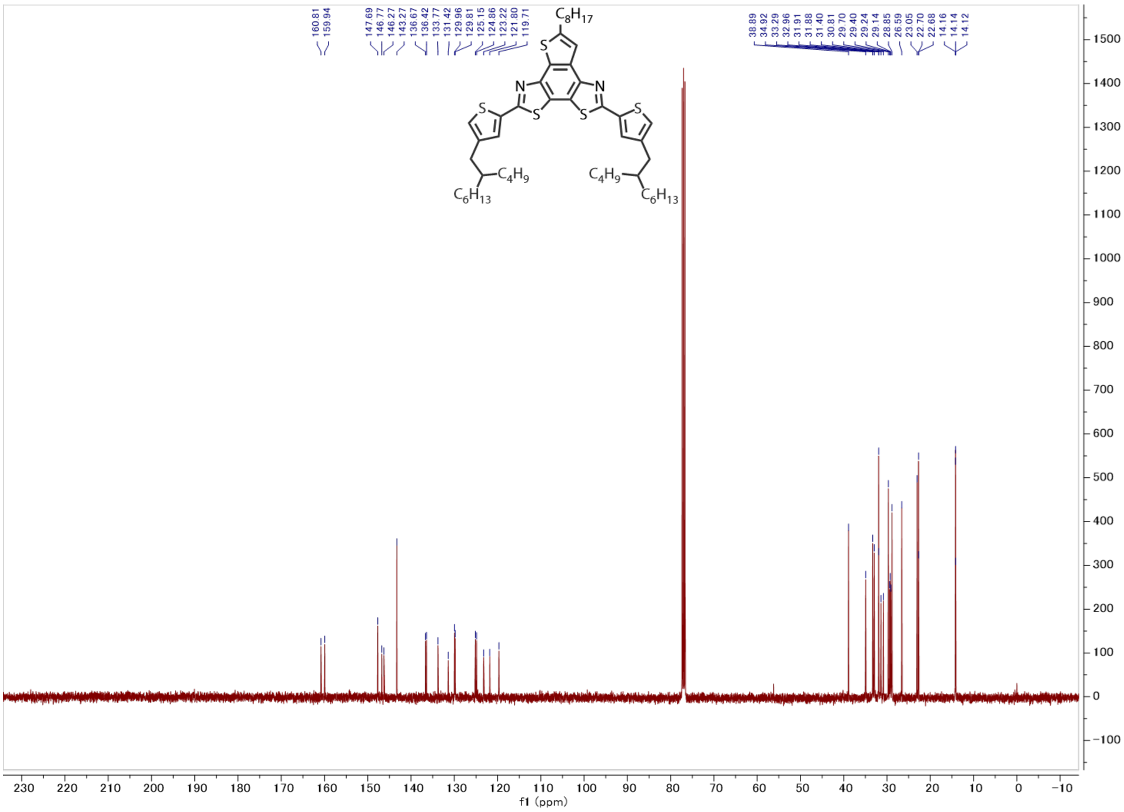

^{1}H-NMR (upper) and ^{13}C-NMR (lower) spectra of **16**

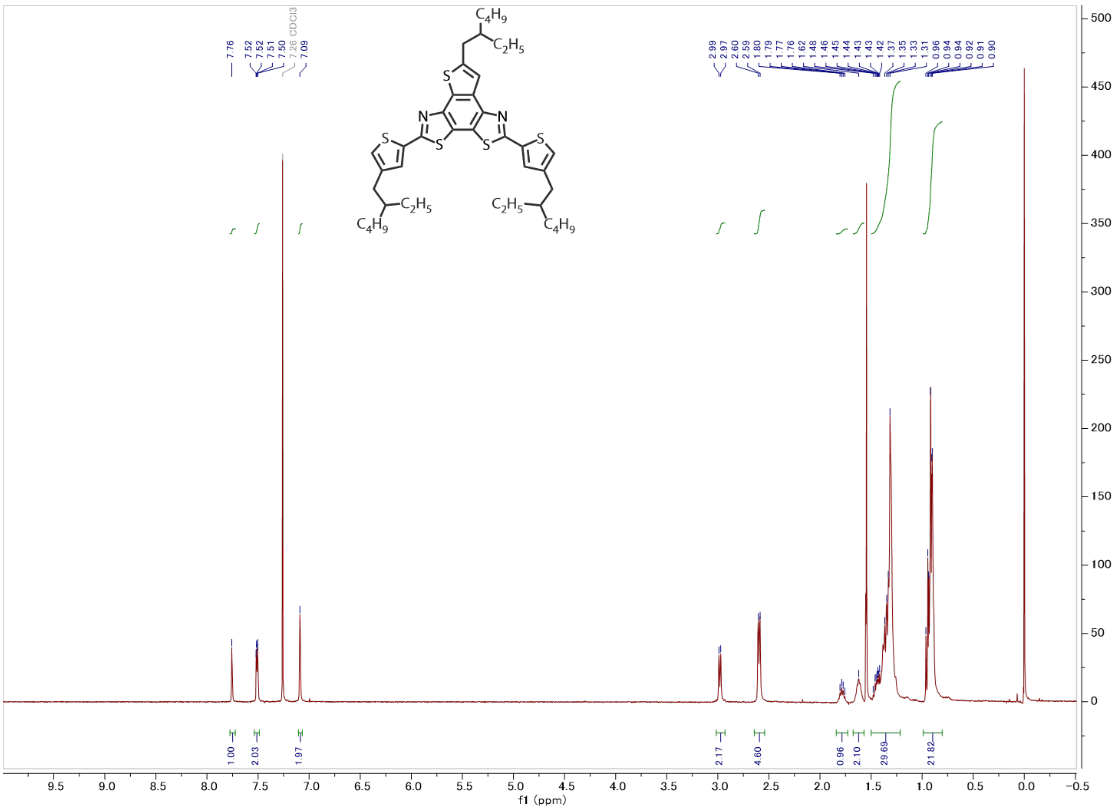

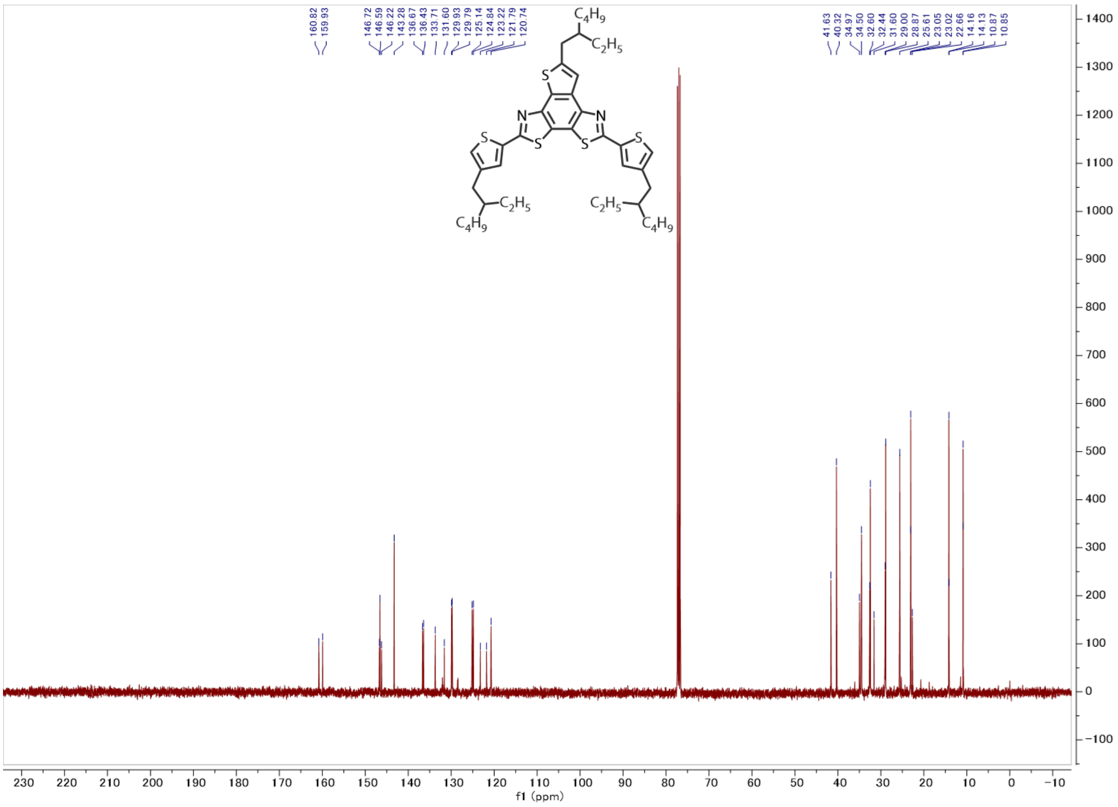

^{1}H-NMR (upper) and ^{13}C-NMR (lower) spectra of **17**

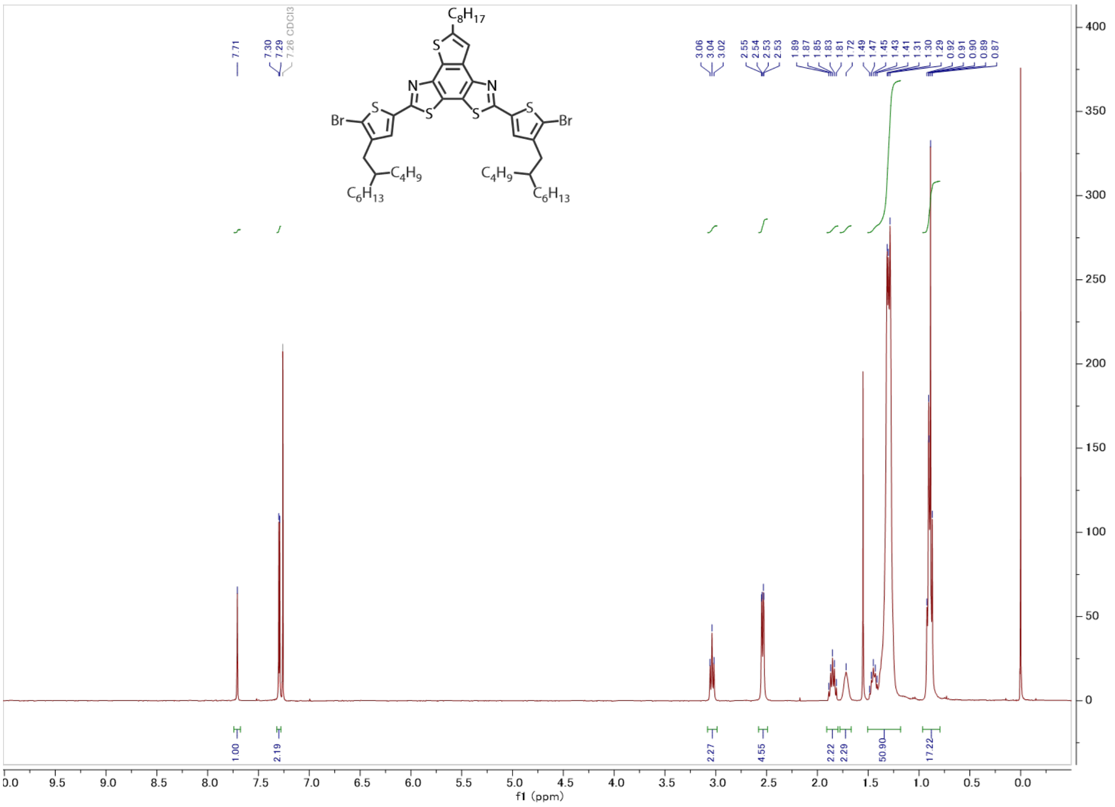

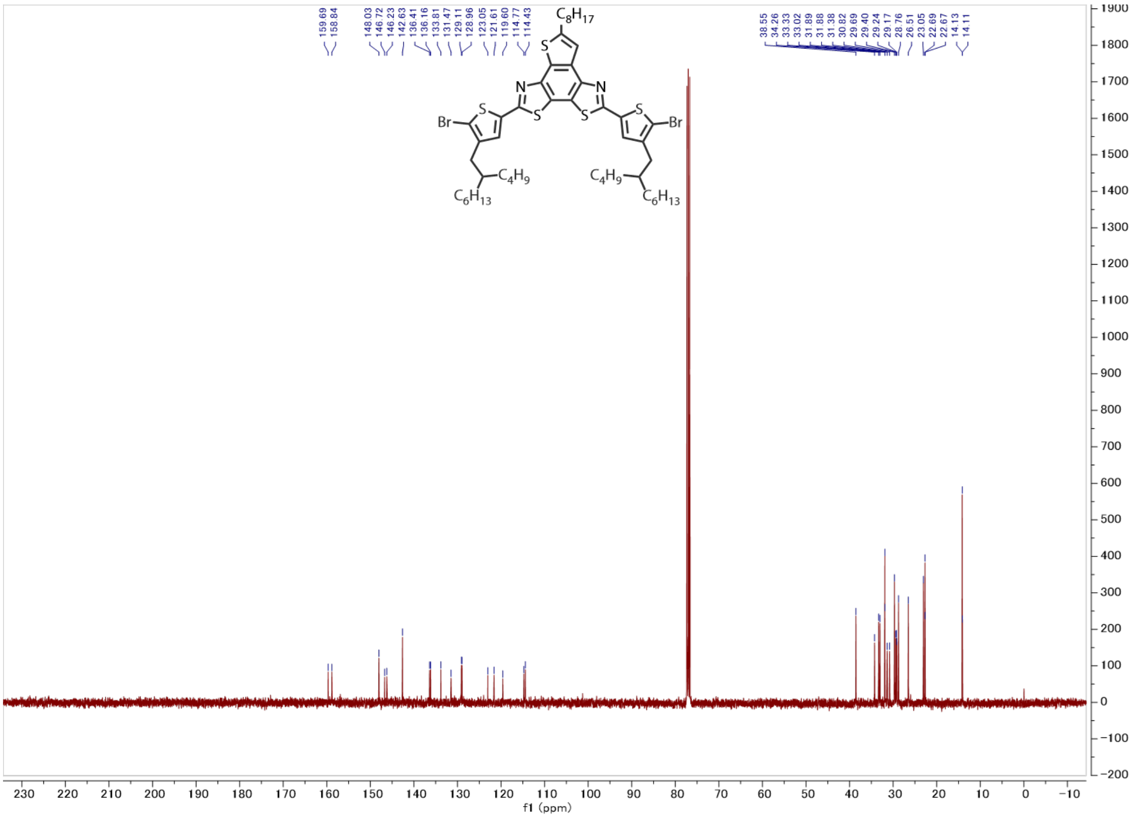

^{1}H-NMR (upper) and ^{13}C-NMR (lower) spectra of **18**

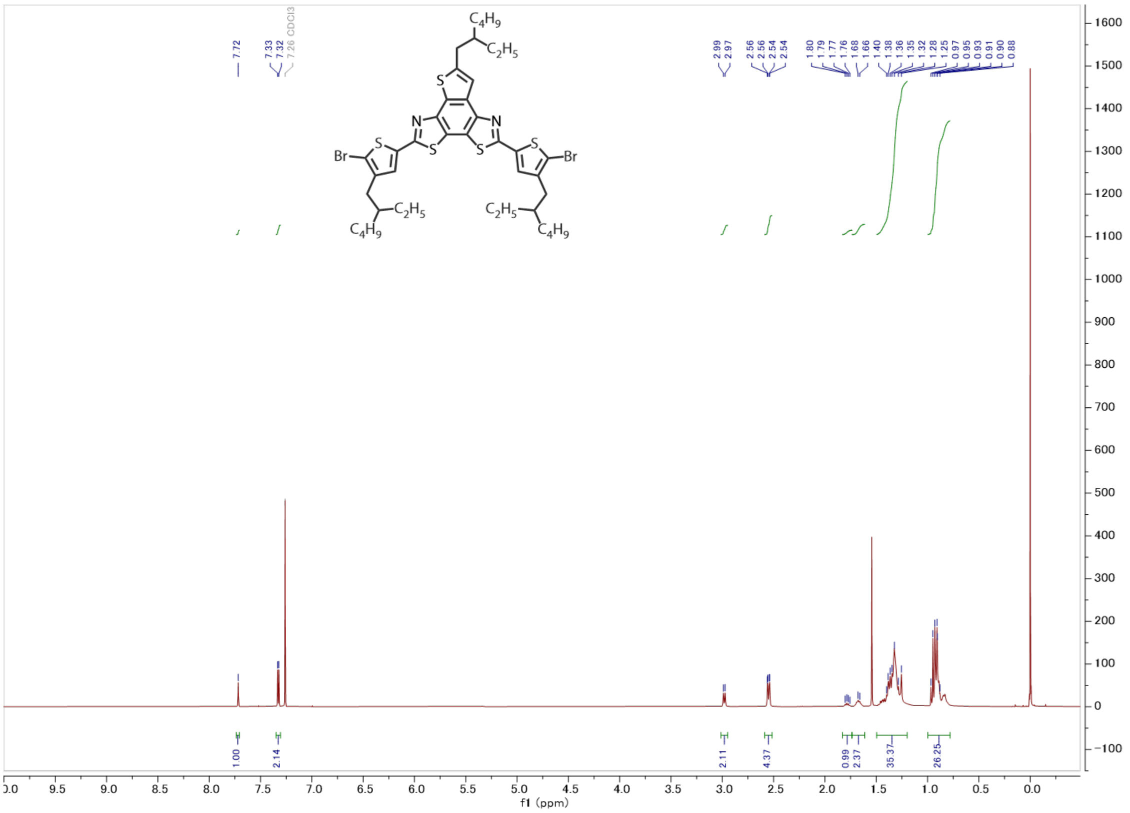

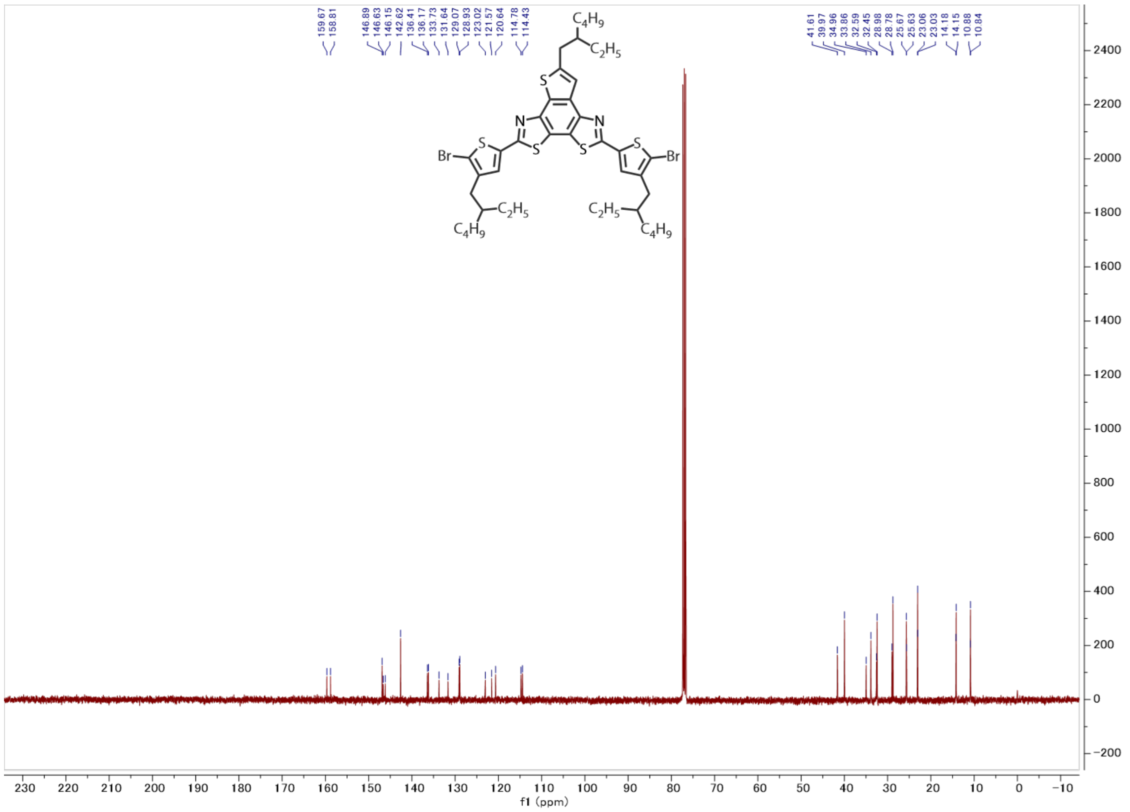

^{1}H-NMR (upper) and ^{13}C-NMR (lower) spectra of **19**

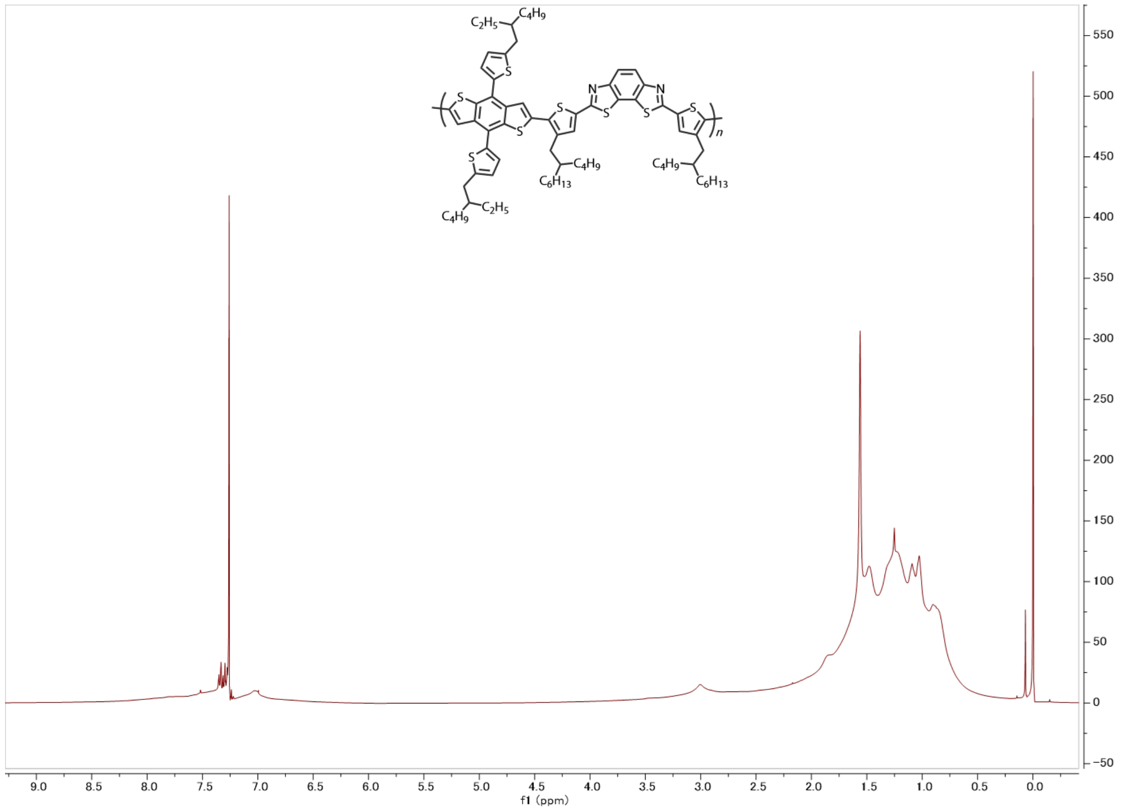

^{1}H-NMR spectrum of **PiNBTz1**

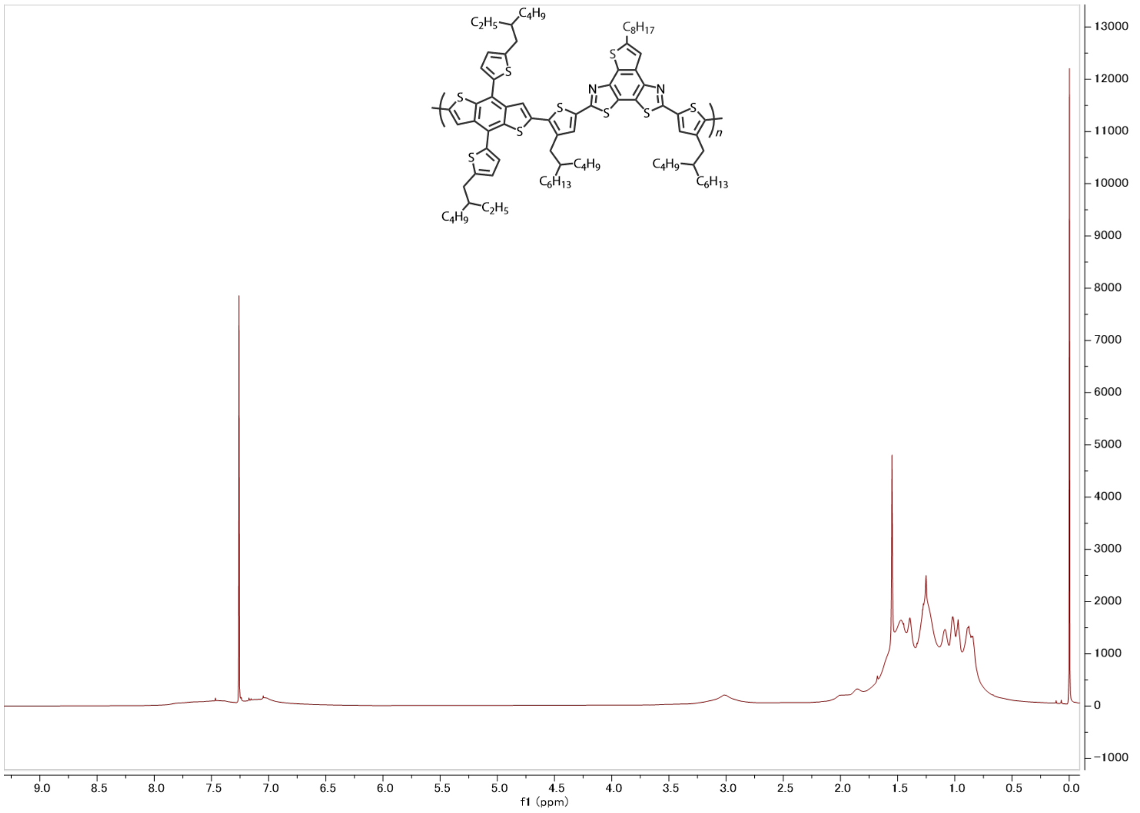

^{1}H-NMR spectrum of **PTBTz1**

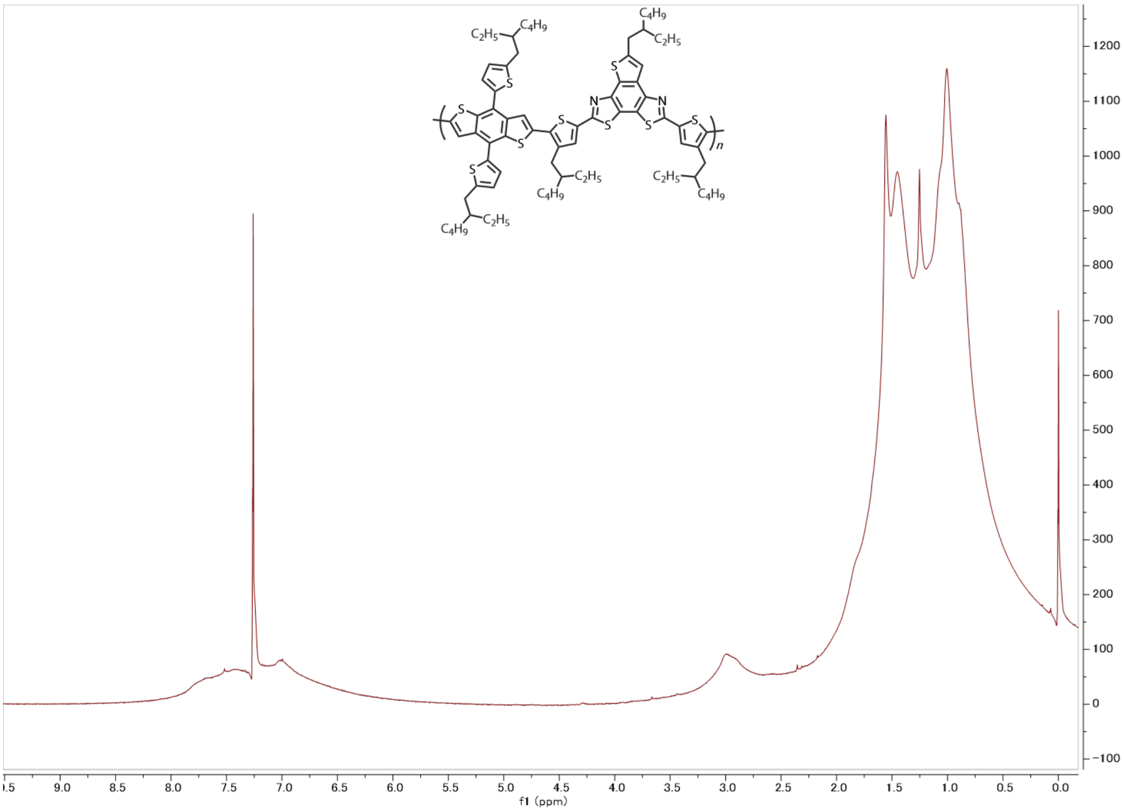

^{1}H-NMR spectrum of **PTBTz2**

Instrumentation

Differential scanning calorimetry analysis was carried out with an EXSTAR DSC7020 (SII Nanotechnology, Inc.) at a cooling and heating rate of 10 °C min^{-1}. Cyclic voltammetry was carried out with an ALS Electrochemical Analyzer Model 612D in acetonitrile containing tetrabutylammonium hexafluorophosphate (Bu_4NPF_6, 0.1 M) as the supporting electrolyte at a scan rate of 100 mV s^{-1}. The counter and working electrodes were made of Pt, and the reference electrode was Ag/Ag$^+$. The samples were fabricated directly on the working electrode by dipping into the solution of the materials. All potentials were calibrated with the standard ferrocene/ferrocenium redox couple (Fc/Fc$^+$: $E^{1/2}$ = +0.18 V measured under the identical conditions). HOMO and LUMO energy levels (E_{HOMO} and E_{LUMO}) were calculated with the following equations S1 and S2:

$$E_{\mathrm{H}}\ (\mathrm{eV}) = -4.62 - E_{\mathrm{ox}} \qquad \text{(S1)}$$

$$E_{\mathrm{L}}\ (\mathrm{eV}) = -4.62 + E_{\mathrm{red}} \qquad \text{(S2)}$$

where E_{ox} and E_{red} are the onset oxidation and reduction potential of cyclic voltammograms, respectively. UV–vis absorption spectra were measured using a Shimadzu UV-3600 spectrometer. Photoemission yield spectroscopy was conducted in N_2 using an AC-3 spectrometer (RIKEN KEIKI, Co. Ltd.). Photoluminescence spectra were measured with a fluorescence spectrometer (Horiba Jobin Yvon, NanoLog) equipped with a photomultiplier tube (Hamamatsu, R928P) and a liquid-nitrogen-cooled InGaAs near-infrared array detector (Horiba Jobin Yvon, Symphony II) under ambient atmosphere. The excitation wavelength was set at 560 and 800 nm to estimate the quenching efficiency of donor polymers and Y6, respectively. Tapping mode atomic force microscopy was carried out on a SPM-9700HT scanning probe microscope (Shimadzu Corp). Transmission electron microscopy (TEM) was conducted on JEM-2021 (JEOL).

Grazing incidence wide-angle X-ray diffraction (GIXD) measurements

GIXD measurements were performed with a HUBER multi-axis diffractometer installed in the beamline BL46XU at SPring-8. The X-ray beam from the undulator was monochromatized by a double-crystal Si(111) monochromator. The X-ray energy was 12.39 keV (λ = 1 Å), and the X-ray beam size was 40 μm (height) × 300 μm (width) at the sample position. The diffraction from the samples was detected by a two-dimensional (2D) X-ray photon counting pixel detector (PILATUS 300 K). The X-ray beam incidence angle was set to 0.12 °, and the camera length (sample-to-detector distance) was set to 174 mm. The neat films were prepared by spin-coating the material solution in chloroform on the PEDOT:PSS coated ITO glass with

the same conditions, where the substrate size was 1 cm × 1 cm. For the blend films, the photoactive layer on the OPV cells were directly used for the measurements. The measurements were performed in air at room temperature. The exposure time was 1 s, and no irradiation damage was observed on the samples. The coherence length (L) was estimated from the simplified Scherrer's equation, $L = 2\pi/\text{fwhm}$,[S8] where fwhm is the full-width at half-maximum of the lamellar and π–π stacking diffraction peaks. Note that fwhm was not corrected for the resolution function typically caused by the sample size.

Time-resolved microwave conductivity (TRMC) measurement

A resonant cavity was used to obtain a high degree of sensitivity in the conductivity measurement. The resonant frequency and microwave power were set at ca. 9.1 GHz and 3 mW, respectively, so that the electric field of the microwave was sufficiently small to not disturb the motion of charge carriers. The third harmonic generation (THG; 355nm) of a Nd:YAG laser (Continuum Inc., Surelite II, 5–8 ns pulse duration, 10 Hz) was used as an excitation source. The incident photon intensity was 9.1×10^{15} photons cm^{-2} pulse^{-1}. The photoconductivity transient $\Delta\sigma$ is converted to the product of the quantum efficiency φ and the sum of charge carrier mobilities $\Sigma\mu$, by $\varphi\Sigma\mu = \Delta\sigma(eI_0F_{\text{light}})^{-1}$, where e and F_{light} are the unit charge of a single electron and a correction (or filling) factor.

Fabrication and measurement of photovoltaic cells

ITO substrates were first pre-cleaned sequentially by sonicating in a detergent bath, de-ionized water, acetone, and isopropanol at room temperature, and in boiled isopropanol each for 10 min, and then baked at 120 °C for 10 min in air. The substrates were subjected to UV/ozone treatment at room temperature for 10 min. The PEDOT:PSS (Clevios P VP Al 4083) layer was deposited on the pre-cleaned ITO substrate by spin-coating at 5000 rpm, 30 s and was then baked at 130 °C for 10 min in air. The photoactive layer was deposited in a glove box (KIYON, KK-011AS-EXTRA) by spin coating a polymer/Y6 solution in chloroform.

Here, we describe the optimal fabrication conditions. For the **PNBTz1**/Y6 blend, 5 g L^{-1} of the chloroform solution containing 0.5 vol% of 1-chloronaphthalene (CN) with the polymer to Y6 ratio of 1:1.2 (w/w), where the concentration was based on the polymer weight, was prepared by stirring it at 50 °C until the materials completely dissolved. The solution was kept at 50 °C and spun at 4000 rpm for 20 s. For the **PiNBTz1**, **PTBTz1**, and **PTBTz2**/Y6 blends, 3.5 g L^{-1} of the chloroform solution containing 0.5 vol% of CN with the polymer to Y6 ratio of 1:1.2 (w/w), where the concentration was based on the polymer weight, was prepared by stirring

it at 50 °C until the materials completely dissolved. After cooling to rt, the solution was spun at 1000 rpm for 20 s. The PDINO layer was then deposited by spin-coating (3000 rpm for 30 s) from 2 g L^{-1} of the methanol solution. When PNDIT-F3N-Br was used instead of PDINO, the PNDIT-F3N-Br solution in methanol was spun (1000 rpm for 30 s). The thin films were transferred into a vacuum evaporator (ALS Technology, E-100J) connected to the glove box. The Ag layer (220 nm) was deposited by thermal evaporation through a shadow mask under ~10^{-5} Pa, where the photoactive area of the cells was 0.04 cm^2.

The *J–V* characteristics of the cells were measured using a Keithley 2400 source–measure unit in a glove box under the 1 sun (AM1.5G) condition using a solar simulator (SAN-EI Electric, XES-40S1). Light intensity was calibrated with a reference PV cell (KONICA MINOLTA AK-100 certified by National Institute of Advanced Industrial Science and Technology, Japan). EQE spectra were measured with a spectral response measurement system (SOMA OPTICS, S-9241). More than 10 different substrates (four photoactive areas each) were prepared and measured for the optimized cells. Photoactive layer thickness was measured with an ET4000 (Kosaka Laboratory, Ltd.), where the optimal photoactive layer thickness was around 100 nm.

Fabrication and measurement of hole- and electron-only devices

For hole-only devices, the pre-cleaned ITO substrates were coated with PEDOT:PSS by spin-coating (5000 rpm for 30 s, thickness of ~30 nm) in air. The polymer/Y6 (1:1.2 w/w) blend film was then fabricated by spin-coating in the glove box as described for the photovoltaic cells. MoO_x (7.5 nm) and Ag (200 nm) layers were deposited sequentially through a shadow mask in the vacuum evaporator. For electron-only devices, the pre-cleaned ITO substrates were coated with ZnO nanoparticle suspension (1200 rpm for 20 s) in air, and then the polymer/Y6 (1:1.2 w/w) blend film in the glove box as mentioned above. The ZnO layer was again deposited by spin-coating (6000 rpm for 20 s) in air, and then the Ag layer (200 nm) was deposited by vacuum evaporation.

The *J–V* characteristics were measured in the range of 0–7 V using a Keithley 2400 source–measure unit in the dark in the glovebox. The *J–V* characteristics were measured at room temperature. The mobility (μ) was calculated by fitting the *J–V* curves according to the space charge limited current model described by the following equation S3:

$$J = (9/8)\, \varepsilon_r \varepsilon_0 \mu (V^2/L^3) \qquad \text{(S3)}$$

where ε_r is the relative dielectric constant of the polymer, ε_0 is the permittivity of free space, $V = V_{appl} - V_{bi}$, where V_{appl} is the applied voltage to the device and V_{bi} is the built-in voltage due

to the difference in work function of the two electrodes, which was determined to be 0.1 V, and L is the polymer thickness. The dielectric constant ε_r is assumed to be 3, which is a typical value for semiconducting polymers.

Contact angle measurement and surface energy calculation.

Droplets of ultrapure water and glycerol were dripped onto the thin film polymers and Y6. According to Owens-Wendt method, surface energy γ could be divided into dispersive (γ^d) and polar (γ^p) components: $\gamma = \gamma^d + \gamma^p$.

The γ^d and γ^p can be calculated through the formula below based on the contact angles obtained by two different solvents,

$$(1 + \cos\theta)\gamma_L = 2\sqrt{(\gamma_S^d \times \gamma_L^d)} + 2\sqrt{(\gamma_S^p \times \gamma_L^p)}$$

where θ is the contact angle of a solvent, γ_L is the surface energy of the solvent, γ_S^d and γ_L^d refer to the dispersive surface energy of the material and the specific solvent, respectively, and γ_S^p and γ_L^p refer to the polar surface energy of the material and the specific solvent, respectively. Thus, the unknown value γ_S^d and γ_S^p can be solved by combining two equations obtained by contact angle measurement of two different solvents.

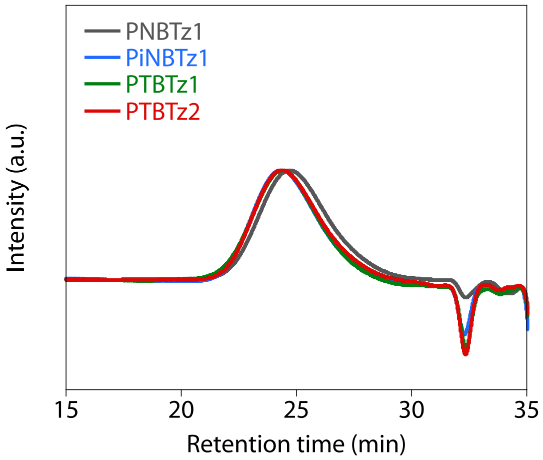

Figure S1. GPC charts of the polymers.

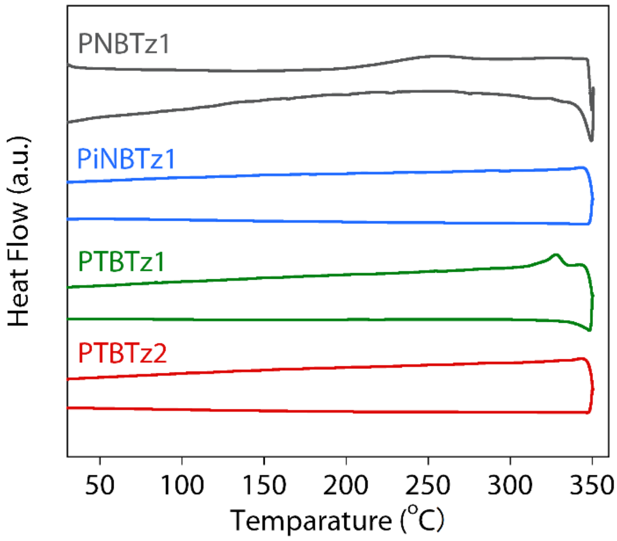

Figure S2. DSC curves of the polymers.

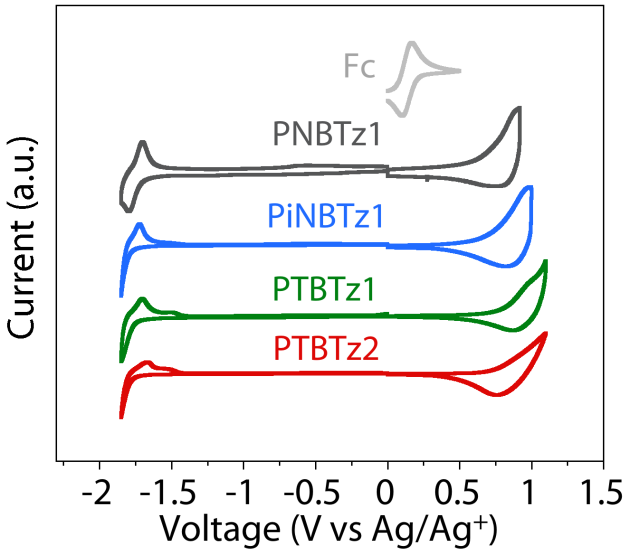

Figure S3. Cyclic voltammograms of the polymers.

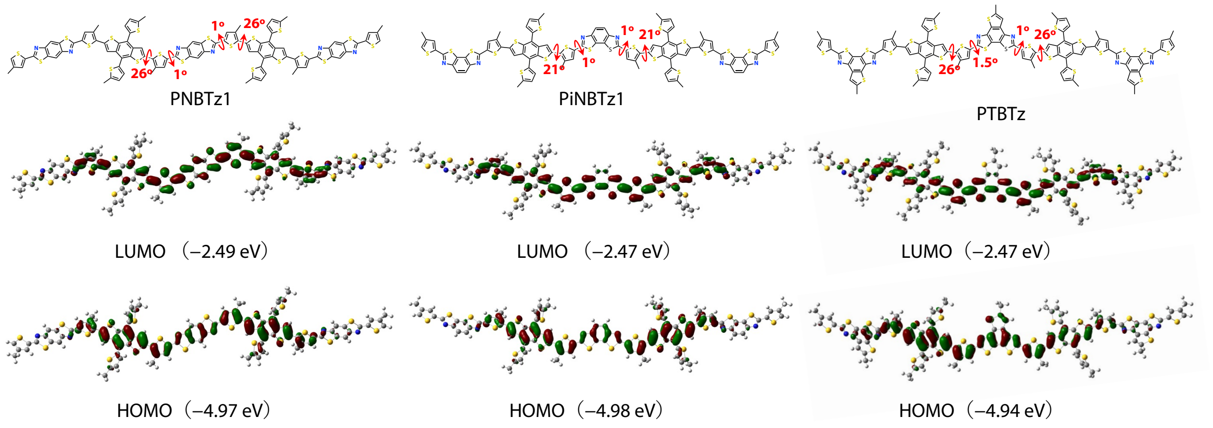

Figure S4. Optimized backbone geometry, HOMO and LUMO geometry, and HOMO and LUMO energy level for the model compounds of **PNBTz1**, **PiNBTz1**, and **PTBTz**s calculated by the DFT method at the B3LYP/6-31G(d) level. All the branched alkyl groups were replaced with the methyl groups to simplify the calculation.

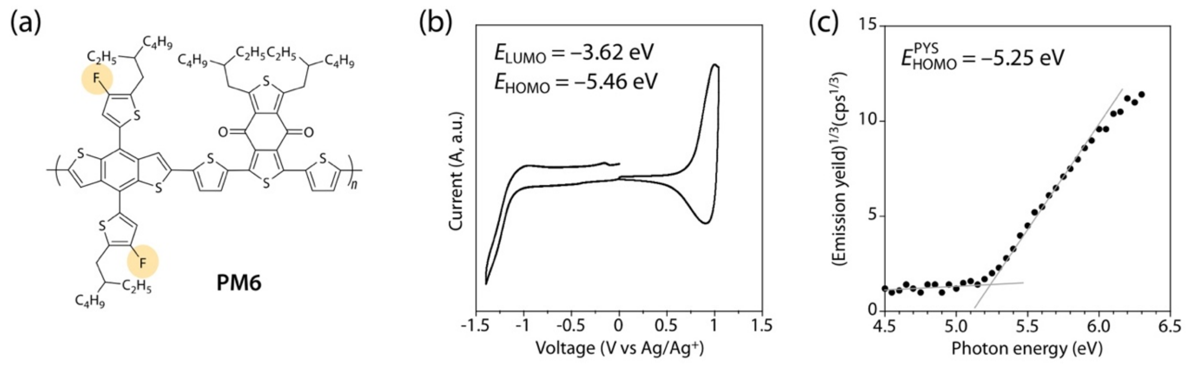

Figure S5. (a) Chemical structure, (b) cyclic voltammogram, and (c) photoemission yield spectrum of PM6.

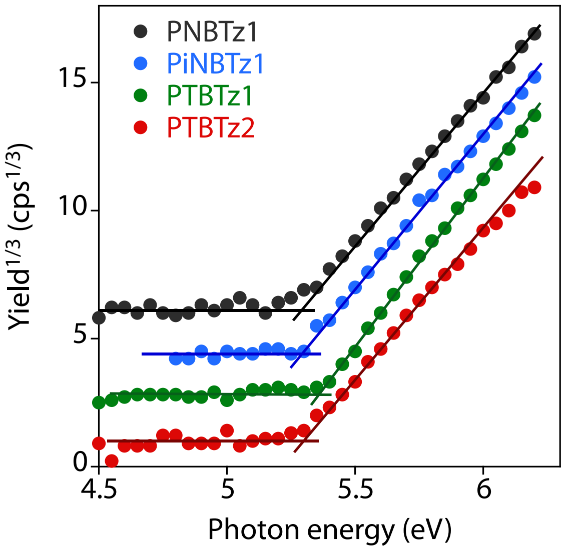

Figure S6. Photoemission yield spectra of the polymers.

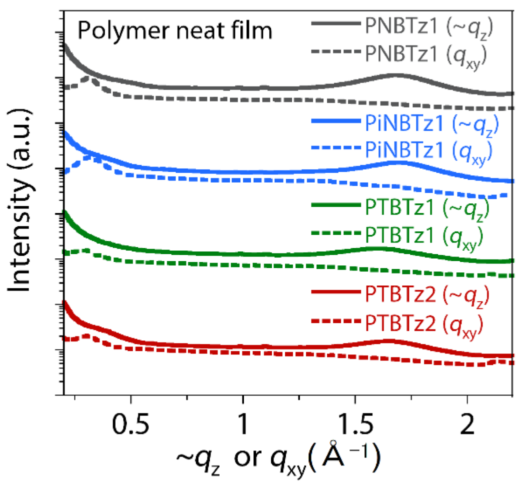

Figure S7. Cross-sectional profiles cut from the 2D GIXD patterns of the polymer neat films.

Table S1. Structural parameters extracted from the GIXD measurements.

film	*d*-spacing (Å)		Coherence length (Å)$^{a)}$
	lamella	π–π stacking	
**PNBTz1**	20.8	3.73	18
**PiNBTz1**	20.1	3.73	16
**PTBTz1**	22.1	3.88	16
**PTBTz2**	20.8	3.77	17

$^{a)}$The coherence length (L) for the π–π stacking order, which was estimated from the simplified Scherrer's equation, $L = 2\pi/\mathrm{fwhm}$, where fwhm is the full-width at half-maximum.

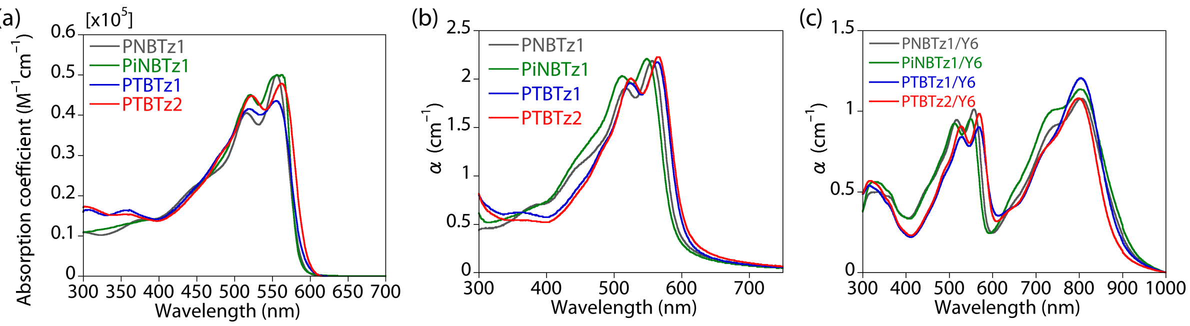

Figure S8. UV–Vis absorption spectra of (a) the polymer solutions, (b) polymer neat films, and (c) polymer/Y6 blend films with the absorption coefficient.

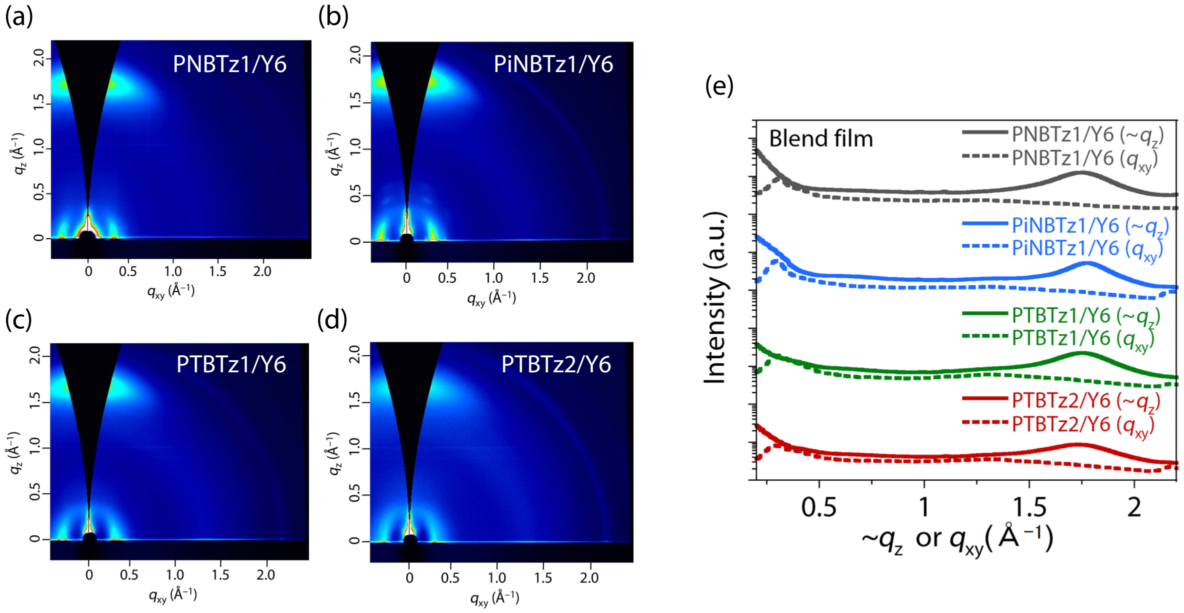

Figure S9. (a–d) 2D GIXD patterns and (e) their cross-sectional profiles of the blend films.

Table S2. Structural parameters extracted from the GIXD measurements.

Blend film	additive	*d*-spacing (Å)		Coherence length
		lamella	π–π stacking	(Å)[a]
**PNBTz1**/Y6	CN	20.6	3.59	18
**PiNBTz1**/Y6	CN	20.9	3.56	19
**PTBTz1**/Y6	CN	20.8	3.61	17
**PTBTz2**/Y6	CN	21.6	3.62	17

[a]The coherence length (L) for the π–π stacking order, which was estimated from the simplified Scherrer's equation, $L = 2\pi/\text{fwhm}$, where fwhm is the full-width at half-maximum.

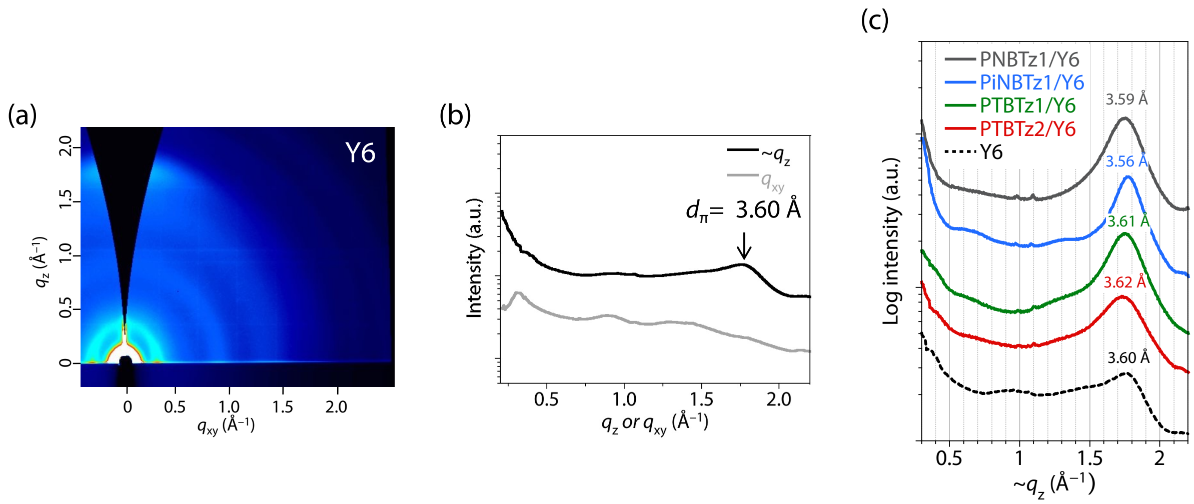

Figure S10. (a) 2D GIXD pattern and (b) line cut diffraction profiles of the Y6 neat film. (c) Comparison of the profiles along the quasi-q_z axis between Y6 neat film and polymer/Y6 blend films.

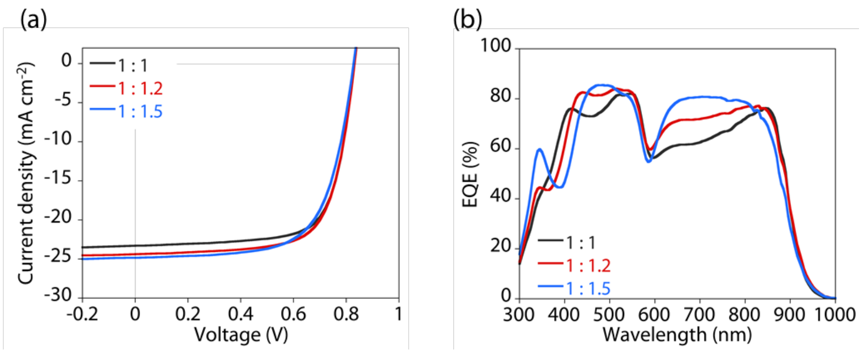

Figure S11. (a) *J–V* curves and (b) EQE spectra of **PiNBTz1**/Y6 cells with different p/n ratios.

Table S3. Photovoltaic properties of **PiNBTz1**/Y6 cells with different p/n ratios.

p/n ratio	J_{SC} (mA cm^{-2})	V_{OC} (V)	FF	PCE$_{max}$ (%)
1 : 1	23.3	0.83	0.72	13.8
1 : 1.2	24.4	0.83	0.70	14.2
1 : 1.5	24.8	0.83	0.67	13.7

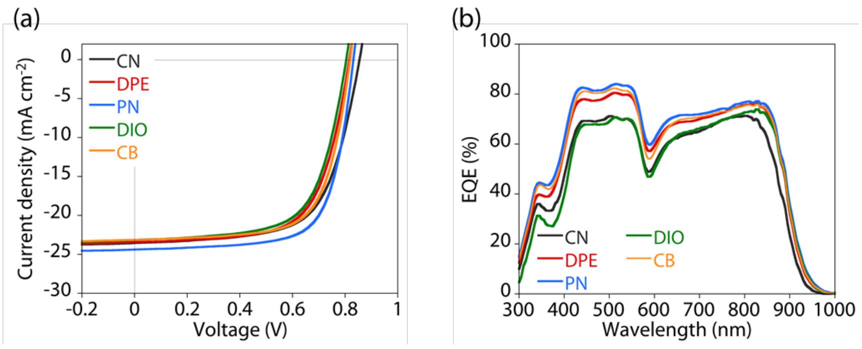

Figure S12. (a) *J–V* curves and (b) EQE spectra of **PiNBTz1**/Y6 cells with different additives.

Table S4. Photovoltaic properties of **PiNBTz1**/Y6 cells with different additives.

additive[a)]	J_{SC} (mA cm^{-2})	V_{OC} (V)	FF	PCE$_{max}$ (%)
CN	23.5	0.85	0.68	13.1
DPE	23.4	0.82	0.66	12.6
PN	24.4	0.83	0.70	14.2
DIO	23.1	0.80	0.66	12.2
CB	23.1	0.82	0.68	12.9

[a)]CN: 1-chloronaphthalene, DPE: diphenylether, PN: 1-phenylnaphthalene, DIO: 1,8-diiodooctane, CB: chlorobenzene.

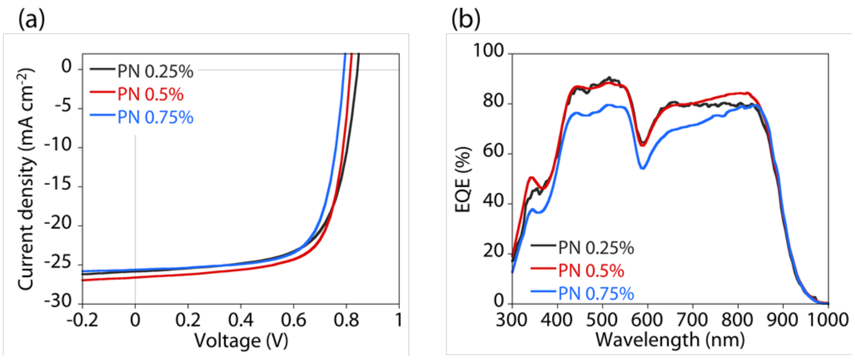

Figure S13. (a) *J*–*V* curves and (b) EQE spectra of **PiNBTz1**/Y6 cells with different volumes of PN.

Table S5. Photovoltaic properties of **PiNBTz1**/Y6 cells with different volumes of PN.

PN (vol%)	J_{SC} (mA cm^{-2})	V_{OC} (V)	FF	PCE$_{max}$ (%)
0.25	25.8	0.84	0.67	14.6
0.5	26.6	0.82	0.71	15.4
0.75	25.6	0.79	0.71	14.4

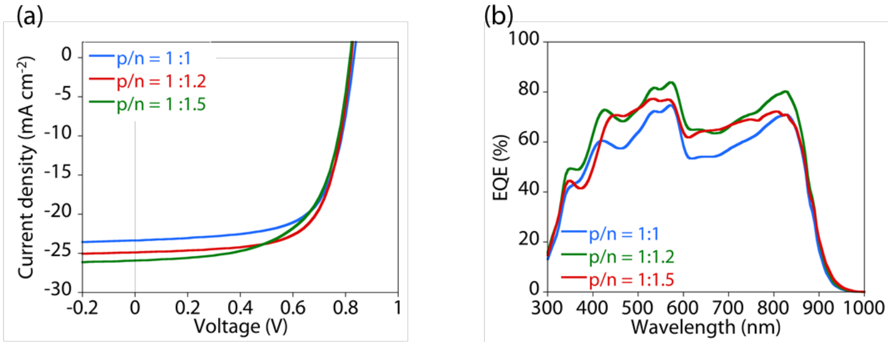

Figure S14. (a) *J–V* curves and (b) EQE spectra of **PTBTz2**/Y6 cells with different p/n ratios.

Table S6. Photovoltaic properties of **PTBTz2**/Y6 cells with different p/n ratios.

p/n ratio	J_{SC} (mA cm^{-2})	V_{OC} (V)	FF	PCE$_{max}$ (%)
1 : 1	23.3	0.83	0.67	13.0
1 : 1.2	24.8	0.82	0.68	14.0
1 : 1.5	25.9	0.81	`0.62	13.2

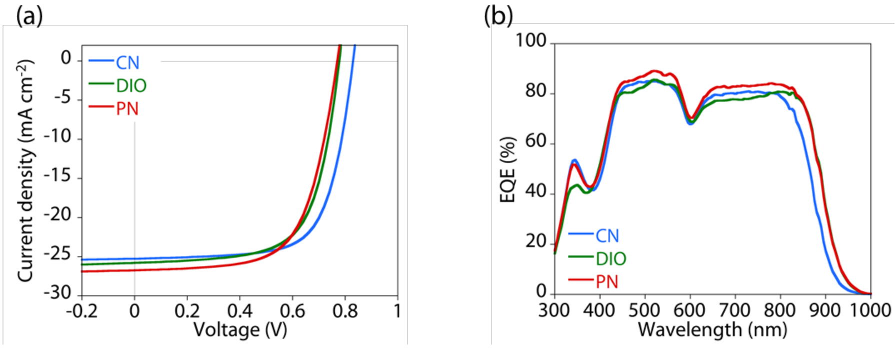

Figure S15. (a) *J–V* curves and (b) EQE spectra of **PTBTz2**/Y6 cells with different additives.

Table S7. Photovoltaic properties of **PTBTz2**/Y6 cells with different additives.

additive	J_{SC} (mA cm^{-2})	V_{OC} (V)	FF	PCE$_{max}$ (%)
CN	25.2	0.83	0.69	14.5
DIO	25.7	0.78	0.67	13.3
PN	26.7	0.77	0.65	13.3

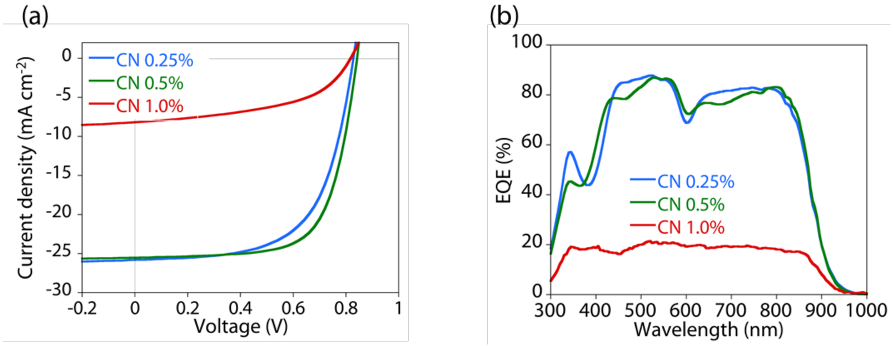

Figure S16. (a) *J–V* curves and (b) EQE spectra of PTBTz2/Y6 cells with different volumes of CN.

Table S8. Photovoltaic properties of PTBTz2/Y6 cells with different volumes of CN.

CN (vol%)	J_{SC} (mA cm^{-2})	V_{OC} (V)	FF	PCE_{max} (%)
0.25	25.7	0.83	0.62	13.3
0.5	25.5	0.84	0.69	14.7
0.75	8.2	0.82	0.50	3.3

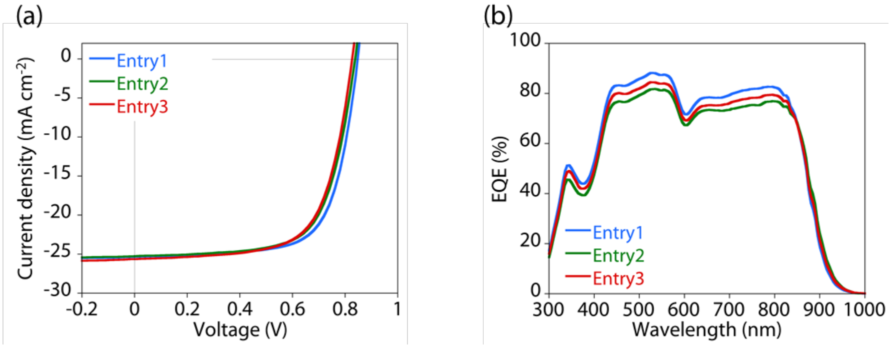

Figure S17. (a) *J–V* curves and (b) EQE spectra of PTBTz2/Y6 cells with or without thermal annealing (TA) and solvent annealing (SA).

Table S9. Photovoltaic properties of PTBTz2/Y6 cells with or without TA and SA.

	TA[a)]	SA	J_{SC} (mA cm^{-2})	V_{OC} (V)	FF	PCE_{max} (%)
Entry1	W	wo	25.3	0.84	0.69	14.9
Entry2	w/o	5 min	25.2	0.83	0.67	14.3
Entry3	w/o	15 min	25.6	0.82	0.67	14.1

[a)] 90 °C, 5 min. [b] chloroform was used.

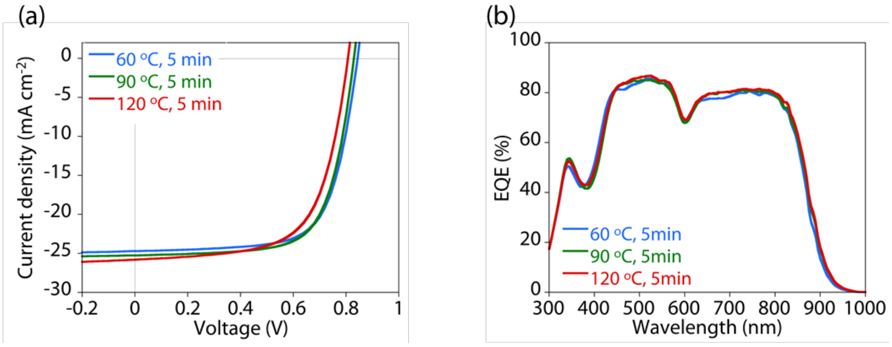

Figure S18. (a) *J–V* curves and (b) EQE spectra of PTBTz2/Y6 cells with different TA temperatures.

Table S10. Photovoltaic properties of PTBTz2/Y6 cells with different TA temperatures.

TA	J_{SC} (mA cm^{-2})	V_{OC} (V)	FF	PCE_{max} (%)
60 °C, 5 min	24.7	0.84	0.69	14.4
90 °C, 5 min	25.2	0.83	0.69	14.5
120 °C, 5 min	25.7	0.81	0.65	13.5

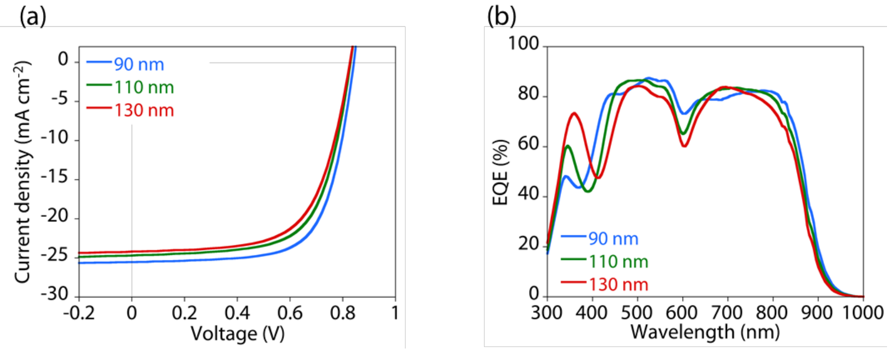

Figure S19. (a) *J–V* curves and (b) EQE spectra of PTBTz2/Y6 cells with different thicknesses.

Table S11. Photovoltaic properties of PTBTz2/Y6 cells with different thicknesses.

thickness (nm)	J_{SC} (mA cm^{-2})	V_{OC} (V)	FF	PCE_{max} (%)
90	25.5	0.84	0.69	14.7
110	24.6	0.83	0.66	13.5
130	24.1	0.83	0.64	12.8

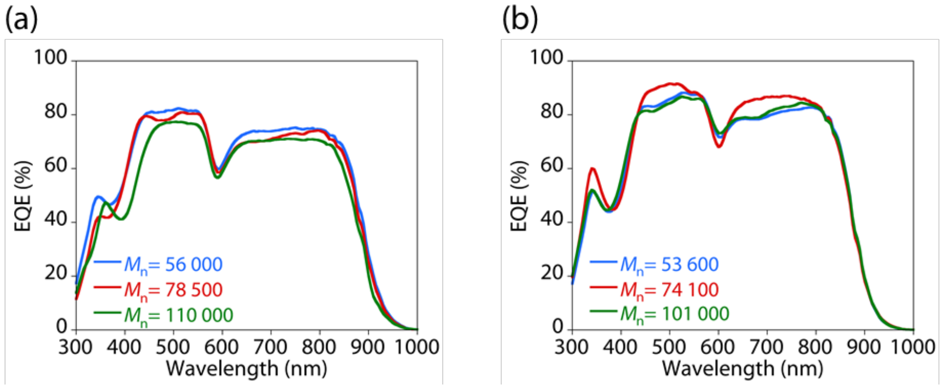

Figure S20. EQE spectra of the (a) **PNBTz1**/Y6, (b) **PTBTz2**/Y6 cells for the polymers with different molecular weights.

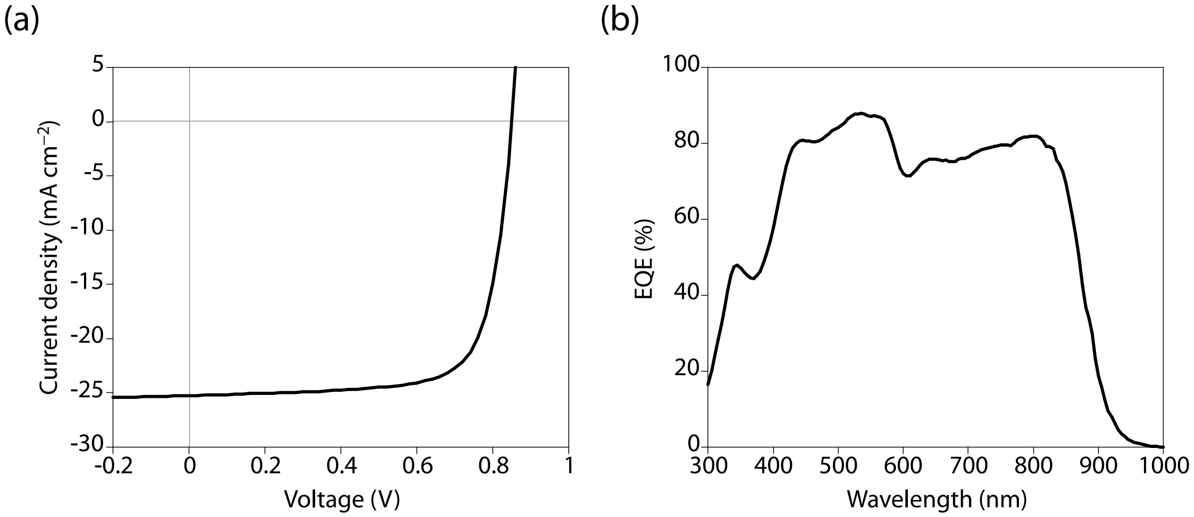

Figure S21. (a) *J–V* curve and (b) EQE spectrum of the PTBTz2/Y6 cell with PNDIT-F3N-Br as the electron transporting layer (ITO/PEDOT:PSS/active layer/PNDIT-F3N-Br/Ag).

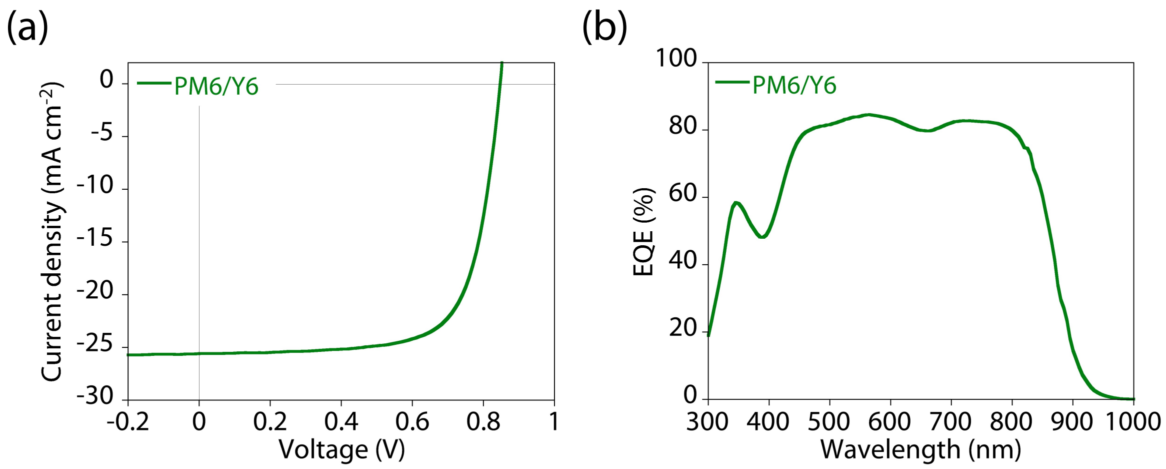

Figure S22. (a) *J*–*V* curve and (b) EQE spectrum of a PM6/Y6 cell.

Table S12. Photovoltaic properties of PM6/Y6 cells.

Active layer	J_{SC} (mA cm^{-2})	V_{OC} (V)	FF	PCE$_{max}$ (%)
PM6/Y6	25.4	0.84	0.72	15.5

Table S13. List of the halogen-free polymers and their OPV performances.

Polymer	E_{HOMO} (eV)	Acceptor	J_{SC} (mA cm^{-2})	V_{OC} (V)	FF	PCE (%)	Ref.
PNBTz1	–5.44	Y6	24.0	0.85	0.72	14.7	This work
PiNBTz1	–5.44	Y6	23.6	0.87	0.66	13.6	This work
PTBTz1	–5.53	Y6	23.6	0.85	0.53	10.6	This work
PTBTz2	–5.45	Y6	25.2	0.85	0.75	15.9	This work
PBDB-T	–5.21	Y6	19.3	0.69	0.65	8.6	[S9]
PBDT-H	–5.35	Y6	24.9	0.74	0.63	11.6	[S10]
PBDTTO	–5.60	Y6	27.0	0.83	0.59	13.3	[S11]
PMT49	–5.35	Y6	25.0	0.81	0.70	14.2	[S12]
PNTB1	–5.42	Y6	24.6	0.83	0.72	14.6	[S13]
L1	–5.45	Y6	24.5	0.83	0.72	14.6	[S14]
D16	–5.48	Y6	25.4	0.85	0.75	16.2	[S14]
TPD-3	–5.14	Y6	26.7	0.83	0.69	15.2	[S15]
PB1	–5.49	BTP-eC9	13.1	0.855	0.48	5.3	[S16]
PB2	–5.48	BTP-eC9	26.2	0.863	0.78	17.7	[S16]
TfBT-4T	–5.33	Y14	25.4	0.83	0.72	15.2	[S17]
PTTz-3HD	–5.36	Y6-BO	27.3	0.77	0.77	16.1	[S18]
PTTz-4HD	–5.43	Y6-BO	26.5	0.80	0.71	15.0	[S18]

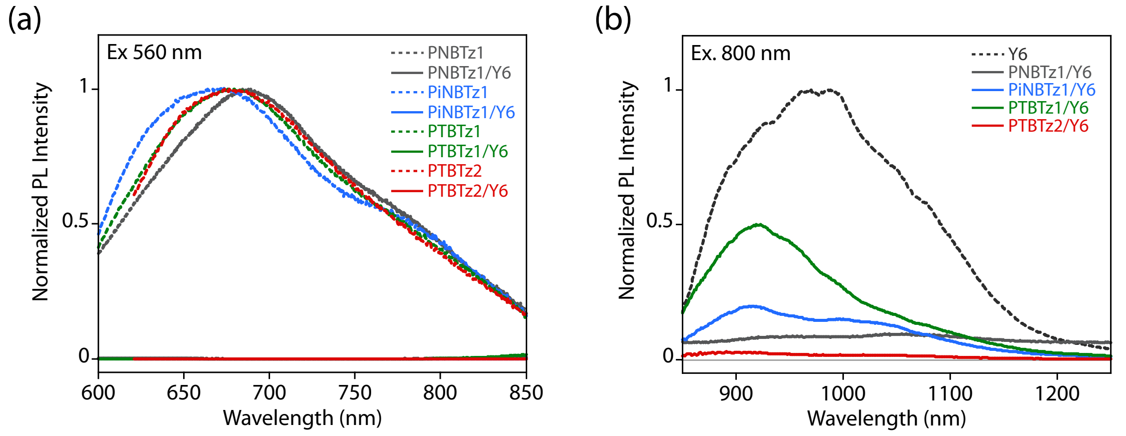

Figure S23. Photoluminescence spectra of the polymers and polymer/Y6 blend films fabricated with CN: (a) **PNBTz1**, (b) **PiNBTz1**, (c) **PTBTz1**, and (d) **PTBTz2**.

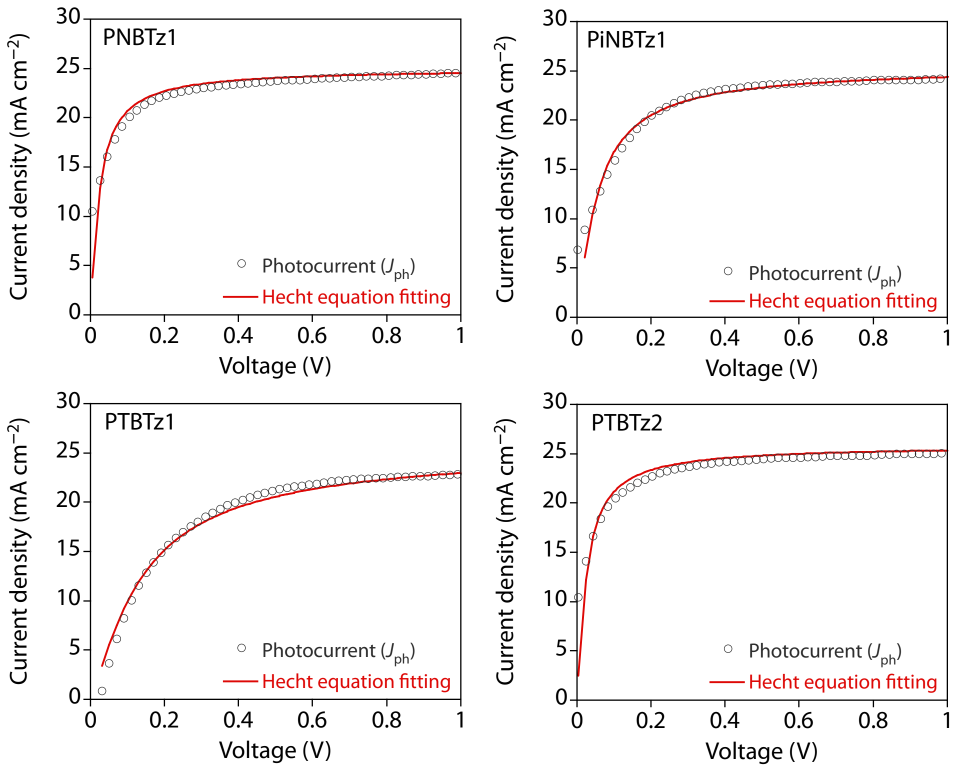

Figure S24. Dependence of the photocurrent (J_{ph}) on the effective voltage (V_{eff}) for the polymer/Y6 cells fabricated with CN.

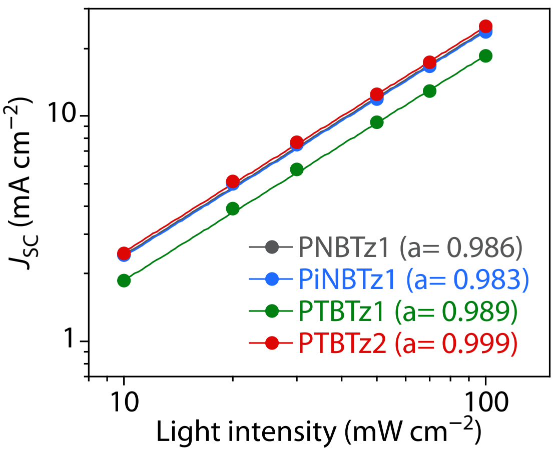

Figure S25. Light intensity dependence of the J_{SC} for the polymer/Y6 cells fabricated with CN.

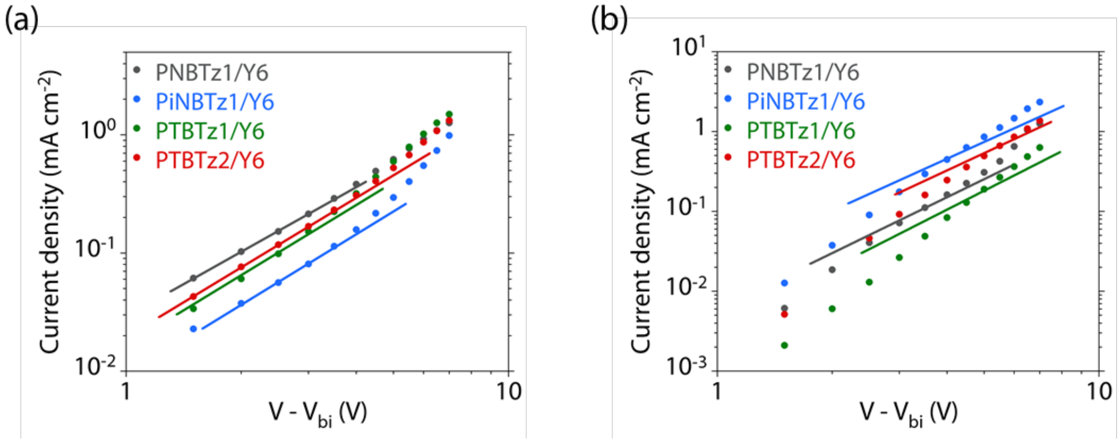

Figure S26. *J–V* curves of the (a) hole-only and (b) electron-only devices based on the polymer/Y6 blend films fabricated by the optimized conditions.

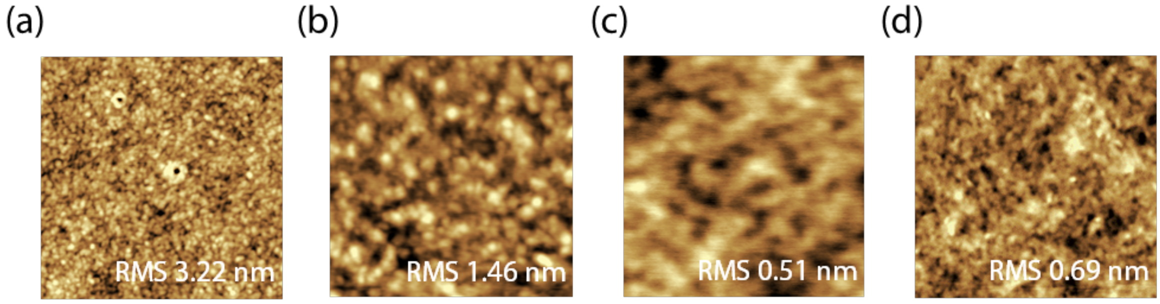

Figure S27. AFM topographic images of the (a) **PNBTz1**/Y6, (b) **PiNBTz1**/Y6, (c) **PTBTz1**/Y6, and (d) **PTBTz2**/Y6 blend films fabricated using CN as additive.

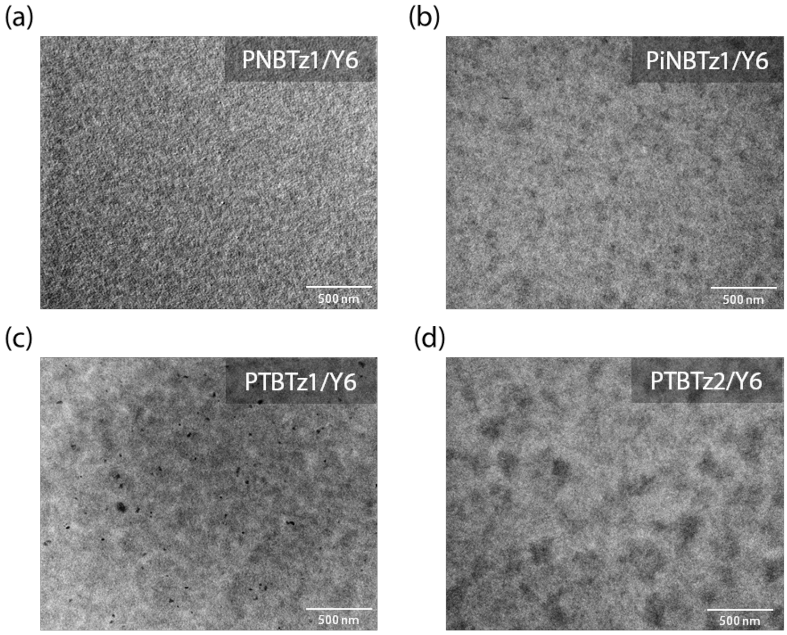

Figure S28. TEM images of (a) **PNBTz1**/Y6, (b) **PiNBTz1**/Y6, (c) **PTBTz1**/Y6, and (d) **PTBTz2**/Y6 blend films fabricated using CN as additive.

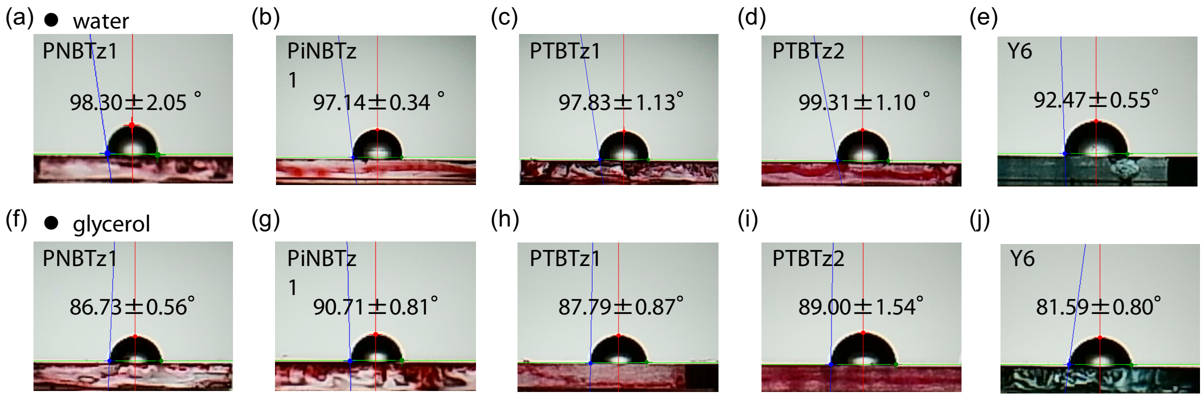

Figure S29. Contact angle of water on (a) PNBTz1, (b) PiNBTz, (c) PTBTz1, (d) PTBTz2, and (e) Y6 films. Contact angle of glycerol on (f) PNBTz1, (g) PiNBTz, (h) PTBTz1, (i) PTBTz2, and (j) Y6 films.

Table S14. Contact angle of water and glycerol for the neat films and the surface energy parameters.

Material	Contact Angle		γ_d (mN m^{-1})^c	γ_p (mN m^{-1})^d	γ (mN m^{-1})^e
	θ_{water} (°)^a	$\theta_{glycerol}$ (°)^b			
PNBTz1	98.30±2.05	86.73±0.56	15.54	7.45	22.99
PiNBTz1	97.14±0.34	90.71±0.81	9.97	11.07	21.04
PTBTz1	97.83±1.13	87.79±0.87	13.77	8.50	22.26
PTBTz2	99.31±1.10	89.00±1.54	13.90	7.80	21.71
Y6	92.47±0.55	81.59±0.80	15.47	10.01	25.48

[a] Contact angle for ultrapure water drops on thin film. [b] Contact angle for glycerol drops on thin film. [c] Dispersive components and [d] polar components calculated by the Owens-Wendt methods. [e] Surface energy γ was estimated by the following equation : $\gamma = \gamma_d + \gamma_p$

References

[S1] Shi, Y.; Guo, H.; Qin, M.; Wang, Y.; Zhao, J.; Sun, H.; Wang, H.; Wang, Y.; Zhou, X.; Facchetti, A.; Lu, X.; Zhou, M.; Guo, X., Imide-Functionalized Thiazole-Based Polymer Semiconductors: Synthesis, Structure–Property Correlations, Charge Carrier Polarity, and Thin-Film Transistor Performance. *Chem. Mater.* **2018**, *30*, 7988–8001.

[S2] Biniek, L.; Fall, S.; Chochos, C. L.; Anokhin, D. V.; Ivanov, D. A.; Leclerc, N.; Lévêque, P.; Heiser, T., Impact of the Alkyl Side Chains on the Optoelectronic Properties of a Series of Photovoltaic Low-Band-Gap Copolymers. *Macromolecules* **2010**, *43*, 9779–9786.

[S3] Gu, P.-Y.; Wang, Z.; Liu, G.; Yao, H.; Wang, Z.; Li, Y.; Zhu, J.; Li, S.; Zhang, Q., Synthesis, Full Characterization, and Field Effect Transistor Behavior of a Stable Pyrene-Fused N-Heteroacene with Twelve Linearly Annulated Six-Membered Rings. *Chem. Mater.* **2017**, *29*, 4172–4175.

[S4] Hrobárik, P.; Hrobáriková, V.; Semak, V.; Kasák, P.; Rakovský, E.; Polyzos, I.; Fakis, M.; Persephonis, P., Quadrupolar Benzobisthiazole-Cored Arylamines as Highly Efficient Two-Photon Absorbing Fluorophores. *Org. Lett.* **2014**, *16*, 6358–6361.

[S5] Čibová, A.; Martinická, A.; Magdolen, P.; Zahradník, P.; Cigáň, M.; Uherek, M.; Chorvát, D., Dicationic and Monocationic Benzobisthiazolium Salts as Potential NLO Chromophores. *Dyes Pigm.* **2018**, *149*, 597–611.

[S6] Xing, Q.; Ma, Y.; Xie, H.; Xiao, F.; Zhang, F.; Deng, G.-J., Iron-Promoted Three-Component 2-Substituted Benzothiazole Formation via Nitroarene ortho-C–H Sulfuration with Elemental Sulfur. *J. Org. Chem.* **2019**, *84*, 1238–1246.

[S7] Shi, Y.; Guo, H.; Qin, M.; Wang, Y.; Zhao, J.; Sun, H.; Wang, H.; Wang, Y.; Zhou, X.; Facchetti, A.; Lu, X.; Zhou, M.; Guo, X., Imide-Functionalized Thiazole-Based Polymer Semiconductors: Synthesis, Structure–Property Correlations, Charge Carrier Polarity, and Thin-Film Transistor Performance. *Chem. Mater.* **2018**, *30*, 7988–8001.

[S8] Roe, R.-J., *Methods of X-Ray and Neutron Scattering in Polymer Science*. Oxford University Press **2010**.

[S9] Wang, Y.; Zhuang, C.; Fang, Y.; Kim, H. D.; Yu, H.; Wang, B.; Ohkita, H., Improvement of Exciton Collection and Light-Harvesting Range in Ternary Blend Polymer Solar Cells Based on Two Non-Fullerene Acceptors. *Nanomaterials* **2020**, *10*, 241.

[S10] Park, J. S.; Kim, G.-U.; Lee, D.; Lee, S.; Ma, B.; Cho, S.; Kim, B. J., Importance of Optimal Crystallinity and Hole Mobility of BDT-Based Polymer Donor for Simultaneous

Enhancements of Voc, Jsc, and FF in Efficient Nonfullerene Organic Solar Cells. *Adv. Funct. Mater.* **2020**, *30*, 2005787.

[S11] He, K.; Kumar, P.; Yuan, Y.; Zhang, Z.; Li, X.; Liu, H.; Wang, J.; Li, Y., A Wide Bandgap Polymer Donor Composed of Benzodithiophene and Oxime-Substituted Thiophene for High-Performance Organic Solar Cells. *ACS Appl. Mater. Interfaces* **2021**, *13*, 26441–26450.

[S12] Zhang, C.-H.; Lin, F.; Huang, W.; Xin, J.; Wang, J.; Lin, Z.; Ma, W.; Yang, T.; Xia, J.; Liang, Y., Methyl functionalization on conjugated side chains for polymer solar cells processed from non-chlorinated solvents. *J. Mater. Chem. C* **2020**, *8*, 11532–11539.

[S13] Ning, H.; Zhang, G.; Chen, H.; Wang, Z.; Ni, S.; Lu, F.; Liu, F.; Dang, L.; Liu, J.; He, F.; Wu, Q., Naphthalenothiophene Imide-Based Polymer Donor for High-Performance Polymer Solar Cells. *Chem. Mater.* **2021**, *33*, 1976–1982.

[S14] Xiong, J.; Jin, K.; Jiang, Y.; Qin, J.; Wang, T.; Liu, J.; Liu, Q.; Peng, H.; Li, X.; Sun, A.; Meng, X.; Zhang, L.; Liu, L.; Li, W.; Fang, Z.; Jia, X.; Xiao, Z.; Feng, Y.; Zhang, X.; Sun, K.; Yang, S.; Shi, S.; Ding, L., Thiolactone copolymer donor gifts organic solar cells a 16.72% efficiency. *Sci. Bull.* **2019**, *64*, 1573–1576.

[S15] Wu, J.; Liao, C.-Y.; Chen, Y.; Jacobberger, R. M.; Huang, W.; Zheng, D.; Tsai, K.-W.; Li, W.-L.; Lu, Z.; Huang, Y.; Wasielewski, M. R.; Chang, Y.-M.; Marks, T. J.; Facchetti, A., To Fluorinate or Not to Fluorinate in Organic Solar Cells: Achieving a Higher PCE of 15.2% when the Donor Polymer is Halogen-Free. *Adv. Energy Mater.* **2021**, *11*, 2102648.

[S16] Zhang, T.; An, C.; Cui, Y.; Zhang, J.; Bi, P.; Yang, C.; Zhang, S.; Hou, J., A Universal Nonhalogenated Polymer Donor for High-Performance Organic Photovoltaic Cells. *Adv. Mater.* **2022**, *34*, 2105803.

[S17] Zhang, Z.; Pan, F.; Luo, M.; Yuan, D.; Liu, H.; Liu, Q.; Wu, Z.; Zhang, L.; Chen, J., A dithienobenzothiadiazole-quaterthiophene wide bandgap polymer enables non-fullerene based polymer solar cells with over 15% efficiency. *Polymer* **2021**, *233*, 124193.

[S18] Yin, B.; Chen, Z.; Pang, S.; Yuan, X.; Liu, Z.; Duan, C.; Huang, F.; Cao, Y., The Renaissance of Oligothiophene-Based Donor–Acceptor Polymers in Organic Solar Cells. *Adv. Energy Mater.* **2022**, *12*, 2104050.
